# Endophilin A2 controls touch and mechanical allodynia via kinesin-mediated Piezo2 trafficking

**DOI:** 10.1186/s40779-024-00520-z

**Published:** 2024-03-12

**Authors:** Man-Xiu Xie, Ren-Chun Lai, Yi-Bin Xiao, Xi Zhang, Xian-Ying Cao, Xiao-Yu Tian, An-Nan Chen, Zi-Yi Chen, Yan Cao, Xiao Li, Xiao-Long Zhang

**Affiliations:** 1 Department of Anesthesiology, Sun Yat-Sen University Cancer Center, State Key Laboratory of Oncology in South China, Guangdong Provincial Clinical Research Center for Cancer, Guangzhou, 510060 China; 2 Medical Research Institute, Guangdong Provincial People’s Hospital (Guangdong Academy of Medical Sciences), Southern Medical University, Guangzhou, 510080 China; 3 Pain Research Center and Department of Physiology, Zhongshan School of Medicine of Sun Yat-Sen University, Guangzhou, 510080 China; 4 Engineering Technology Research Center for Elderly Health Management in Hainan Province, Haikou, 571137 China; 5 College of Food Science and Technology, Hainan University, Haikou, 570228 China

**Keywords:** Endophilin A2, Touch, Mechanical allodynia, Piezo2, KIF5B

## Abstract

**Background:**

Tactile and mechanical pain are crucial to our interaction with the environment, yet the underpinning molecular mechanism is still elusive. Endophilin A2 (EndoA2) is an evolutionarily conserved protein that is documented in the endocytosis pathway. However, the role of EndoA2 in the regulation of mechanical sensitivity and its underlying mechanisms are currently unclear.

**Methods:**

Male and female C57BL/6 mice (8–12 weeks) and male cynomolgus monkeys (7–10 years old) were used in our experiments. Nerve injury-, inflammatory-, and chemotherapy-induced pathological pain models were established for this study. Behavioral tests of touch, mechanical pain, heat pain, and cold pain were performed in mice and nonhuman primates. Western blotting, immunostaining, co-immunoprecipitation, proximity ligation and patch-clamp recordings were performed to gain insight into the mechanisms.

**Results:**

The results showed that EndoA2 was primarily distributed in neurofilament-200-positive (NF200^+^) medium-to-large diameter dorsal root ganglion (DRG) neurons of mice and humans. Loss of EndoA2 in mouse NF200^+^ DRG neurons selectively impaired the tactile and mechanical allodynia. Furthermore, EndoA2 interacted with the mechanically sensitive ion channel Piezo2 and promoted the membrane trafficking of Piezo2 in DRG neurons. Moreover, as an adaptor protein, EndoA2 also bound to kinesin family member 5B (KIF5B), which was involved in the EndoA2-mediated membrane trafficking process of Piezo2. Loss of EndoA2 in mouse DRG neurons damaged Piezo2-mediated rapidly adapting mechanically activated currents, and re-expression of EndoA2 rescued the MA currents. In addition, interference with EndoA2 also suppressed touch sensitivity and mechanical hypersensitivity in nonhuman primates.

**Conclusions:**

Our data reveal that the KIF5B/EndoA2/Piezo2 complex is essential for Piezo2 trafficking and for sustaining transmission of touch and mechanical hypersensitivity signals. EndoA2 regulates touch and mechanical allodynia via kinesin-mediated Piezo2 trafficking in sensory neurons. Our findings identify a potential new target for the treatment of mechanical pain.

**Supplementary Information:**

The online version contains supplementary material available at 10.1186/s40779-024-00520-z.

## Background

The senses of tactile and mechanical pain are critical to our interactions with the environment. Innocuous and noxious mechanical stimuli are sensed by sensory neurons called low-threshold mechanoreceptors (LTMRs) and nociceptors, respectively [1–3]. Clinical observations show that pain can be evoked by innocuous low-threshold mechanical stimulation in patients with inflammation or nerve injury, and this is called mechanical allodynia [4, 5]. Mechanical allodynia is the leading symptom in clinical pain and manifests mainly in two forms: punctate allodynia is evoked by slight pressure on the skin (e.g., von Frey filament), and dynamic allodynia is elicited by gentle moving stimulation (e.g., brush stroke) [6–9]. Mouse pain models simulating punctate and dynamic allodynia symptoms have been used to study these abnormal pain pathways [10, 11]. However, little is known about the molecular mechanisms that mediate sensitized mechanical pain at the periphery.

Endophilin A2 (EndoA2) is an evolutionarily conserved protein that is widely expressed in neural and nonneural tissues and is involved in the regulation of various physiological and pathological processes [12, 13]. A previous study showed that EndoA2 promotes plasma membrane repair and parasitic invasion by regulating clathrin-independent endocytosis [14]. In smooth muscle cells, EndoA2 reduces the expression of the calcium-activated chloride channel TMEM16A by regulating its ubiquitination and autophagy and participates in hypertension-induced vascular remodeling [15]. Knockout of *EndoA2* can inhibit endothelial cell migration and reduce pathological angiogenesis by decreasing the internalization of vascular endothelial growth factor receptor (VEGFR) [16]. EndoA2, expressed in the central nervous system, participates in endocytosis of synaptic vesicle membranes to regulate synaptic transmission that may be related to neurological defects, including epilepsy and neurodegeneration [17, 18]. However, the distribution pattern of EndoA2 in the peripheral nervous system and its role in mechanosensation remain uncertain.

Piezo2 is a mechanically activated (MA) nonselective cation channel that is highly expressed in human and mouse sensory neurons to detect mechanical force signals related to touch and mechanical pain [19, 20]. Studies have shown that mutant Piezo2 in sensory neurons can significantly inhibit tactile sensitivity, punctate allodynia and dynamic allodynia in mice [19, 20]. Piezo2 is known to be a mechanically sensitive ion channel expressed in cell membranes, but the molecular mechanism of Piezo2 membrane trafficking has not been elucidated. Research has provided evidence of the interaction between EndoA2 and the chloride channel 3 (ClC-3), leading to the regulation of ClC-3 membrane trafficking in vascular smooth muscle cells [21]. However, it remains unclear whether EndoA2 is involved in regulating the membrane trafficking of Piezo2 in sensory neurons.

Kinesin superfamily proteins (KIFs) are cytoskeletal motor proteins that move along microtubules in cells [22]. Kinesin proteins can convert the chemical energy stored in ATP into mechanical kinetic energy and provide power for the trafficking of cargo proteins in various cell types [22]. Our previous study has shown that KIF3A is involved in the regulation of sodium channel Nav1.6 membrane trafficking in dorsal root ganglion (DRG) neurons, and it promotes the occurrence and development of neuropathic pain induced by the chemotherapy drug oxaliplatin [23]. Also the kinesin protein KIF17 can mediate the membrane trafficking of the heat-sensitive transient receptor potential M3 (TRPM3) cation channels in sensory neurons, and then it participates in the regulation of heat nociception [24]. Peer research has demonstrated that overexpressing KIF5B promotes the axonal distribution of Nav1.8 in sensory neurons [25]. Subcellular fractionation from the striatum shows that deletion of KIF5B reduces the amount of dopamine D2 receptor in synaptic plasma membranes [26]. Suppressing the function of KIF5B significantly reduces the axonal targeting and forward trafficking of Kv3.1 channels [27], and overexpression of KIF5B increases the surface levels of N-methyl-D-aspartate receptor (NMDAR) and decreases neuronal susceptibility to NMDA-induced excitotoxicity [28]. However, whether the motor protein kinesin participates in the membrane trafficking of Piezo2 and the specific role of EndoA2 in this process are currently unclear.

## Methods

### Animals/mice

C57BL/6 mice were obtained from the Institute of Experimental Animals of Sun Yat-sen University. *EndoA2*^*fl/fl*^, *AvCreERT2*, *Nefh-Cre*, *Trpv1-Cre* and *Piezo2*^*fl/fl*^ mice were purchased from Cyagen Biosciences Inc., China. A total of 739 mice were used in our experiments. All animals were housed in individually ventilated cages (no more than 5 mice per cage) in a temperature-controlled [(24 ± 1) °C] and humidity-controlled (50–60%) room under a 12-h/12-h light/dark cycle. The mice had ad libitum access to sterile water and standard laboratory chow. All animal experimental procedures were approved by the Research Ethics Committee of Sun Yat-sen University Cancer Center (L102012018009J) and were carried out in accordance with the guidelines of the National Institutes of Health on animal care and ethics [29]. Unless otherwise noted, all experiments were conducted in adult (8–12 weeks old) mice. Animals were randomly assigned to each experimental group. Both males and females were included in each group in a sex-matched manner. The data from both sexes were combined and used relatively equally throughout this study, as no sex differences were observed. The complete sample sizes and sex are summarized in Additional file 1: Table S1.

### Mice pain models

To produce inflammatory pain, complete Freund’s adjuvant (CFA, 20 µl) was injected into the plantar surface of the hindpaw following a previously described method [30]. To produce spinal nerve ligation (SNL) neuropathic pain, the mice were anesthetized, and the left L_5_ spinal nerve was isolated adjacent to the vertebral column and tightly ligated with 4–0 silk sutures as previously described [24]. The L_5_ spinal nerves of sham-operated mice were identically exposed but not ligated. To produce chemotherapy pain, vincristine (VCR) was injected at a 0.1 mg/kg dose for 7 consecutive days in mice, according to previous work [31]. In our research, we utilized 133 mice for the CFA model, 112 mice for the SNL model, and 58 mice for the VCR model.

### Behavioural tests in mice

The mice were habituated to the environment for at least 2 d before testing. All the behavioural tests were performed in a blinded manner. The mice with different genotypes or treatments were conducted multiple behavioral tests over a span of 4 d in the following order: von Frey, dynamic and tail clip (Day 1); cotton, back tape and foot tape (Day 2); Hargraves, cooling and pinprick (Day 3); and tail-flick (Day 4). The interval between different tests was at least 2 h. Following behavioral tests, different cohorts of mice were used to perform pain models, molecular biology and electrophysiology experiments (Additional file 2: Fig. S1a).

#### Von Frey test

The von Frey test was performed as previously described [32, 33]. The mice were placed in plastic chambers on a mesh floor. Von Frey filaments with increasing grades of force were applied to the hindpaws of the mice. Each filament was applied 5 times during the test. The lowest filament force that elicited paw withdrawal more than 3 times during the test was defined as the mechanical threshold.

#### Dynamic test

The dynamic test was evaluated in accordance with previously provided instructions [6]. Mice were acclimated in von Frey chambers. The lateral side of the hindpaw was gently stroked with a 5/0 brush from heel to toe. Responses were scored as follows: 0 = no response; 1 = very short, fast movement/lifting of the paw; 2 = sustained lifting of the paw for more than 2 s toward the body or strong lateral lifting above the body level; and 3 = flinching, licking, or flicking of the affected paw. The average score of 3 trials per mouse at least 3 min interval was reported as the allodynia score.

#### Tail clip test

According to our previous work [24], a small alligator clip (force, 700 × g) was applied 1 cm from the base of the tail. The latency to attack/bite the clip was measured with a stopwatch. Upon attack, the clip was removed, and the animals were returned to their cages. A cutoff of 30 s was applied to prevent tissue damage.

#### Cotton test

The cotton test was assessed as described earlier [19]. Mice were acclimated in von Frey chambers. A cotton swab from a Q-tip was manually pulled such that it was puffed out to 3 times its size. A paw withdrawal motion in response to a stroke of the swab underneath the mouse paw was scored. Five sweeps were performed with at least 10 s between each. The number of withdrawals out of 5 trials was counted and reported as the percentage of withdrawal for each mouse.

#### Back tape test

The back tape test was conducted following a previous description [20]. The mice were conditioned to a round plexiglass container. A 1-inch piece of laboratory tape was gently applied to the bottom center of the mouse’s back. The mice were observed for 5 min, and the total number of responses to the tape was recorded. Biting or grabbing the tape or making an obvious “wet dog shake” movement to remove the tape from the back was considered a response.

#### Foot tape test

The foot tape experiment was performed using a method described previously [34]. Mice were placed in a cylindrical container with a diameter of 15 cm and a circular piece of tape with a diameter of 0.2 cm was placed on the sole of the foot. The mice were allowed to move freely and the time was recorded from affixing the tape to the time when the mice found it, up to 5 min. Timing was ended when mice began licking, flapping, or staring at the sole of the foot with the piece of tape. Each mouse was tested individually.

#### Hargreaves test

Thermal hypersensitivity was measured using a plantar test according to the method described by Hargreaves [35]. Briefly, a radiant heat source beneath a glass floor was aimed at the fat pad on the plantar surface of the hindpaw. The latency to withdraw the hindpaw was measured.

#### Cooling test

The mice were habituated to a plastic chamber on a mesh floor. A drop (10–20 μl) of acetone was applied to the hindpaws of the mice. The duration of flinching or licking behaviors within 1 min was measured following a previous description [7].

#### Pinprick test

The pinprick test was performed as described previously [20]. The mice were acclimated to a von Frey chamber for 30 min, and 27G needles were applied to the glabrous skin of the hindpaw, taking care not to pierce the skin. Each mouse was tested 10 times at an interval of 1 min. Paw withdrawal, shaking, or licking was scored as a response, and the percentage of responses to the total number of tests was recorded.

#### Tail-flick test

According to our previous method [24], the mice were gently restrained inside a cloth/cardboard pocket with their tails outside the pocket. The distal half of the tail was immersed in a 48, 50 or 52 °C water bath, and the latency to vigorous withdrawal of the tail from the water was measured. Each animal underwent all 3 temperature tests (48, 50, and 52 °C), with 3 replicates per temperature. There was a minimum interval of 10 min between each replicate. The average of the 3 replicates was used as the withdrawal latency for the mice. The cutoff times of 48, 50, and 52 °C were 20, 15, or 10 s, respectively.

#### Rotarod test

According to our previous method [24], the mice were tested on a rotarod with a velocity that increased from 4 to 40 rpm within 5 min. The mice were pretrained for 2 d for adaptation. Then, the amount of time that each mouse spent on the rotarod before it fell off was recorded.

#### Conditional place aversion (CPA) assay

The CPA test was performed as previously described [6]. The CPA device consists of two chambers, a dark chamber (A) and a light chamber (B), with a metal mesh floor. A black (facing A) and white plastic (facing B) wall with a rectangular hole in the bottom center was inserted into the center of the two chambers. The amount of time a mouse spent in chamber A was recorded. On Day 1, each mouse was placed in the bright compartment (B) and allowed to explore freely between chambers A and B for 15 min (pretest). Under the device’s design, most naïve mice initially showed a preference for the dark chamber. The mice were trained for 4 d. On Days 2 and 4, the hole in the central wall was blocked, and the mouse was placed in chamber B for 20 min. On Days 3 and 5, the hole in the central wall was blocked, and the mouse was placed in chamber A. Then, the injured hindpaw was stimulated with punctate (0.4 g), dynamic (paintbrush from heel to toe), and Hargreaves (cutoff 10 s) stimuli for a continuous duration of 20 min. The interval between punctate and dynamic stimuli was approximately 2 s, while the interval for Hargreaves stimuli was approximately 40 s. On Day 6, the hole in the central wall was opened. First, the mice were placed in a bright compartment and allowed to explore the whole device freely for 15 min to test the compartment preference of the mice (posttest). The aversion score was measured as the difference in time spent in the dark compartment during pretest versus during posttest: aversion score (s) = (pretest time in dark chamber)–(posttest time in dark chamber).

### Cell culture and transfection

Mouse L_4_–L_6_ DRGs were freed from their connective tissue sheaths and broken into pieces with a pair of sclerotic scissors in DMEM/F12 (Gibco, USA) at a low temperature, following a previously described method [24]. Following enzymatic and mechanical dissociation, the DRG neurons were plated on glass coverslips coated with poly-L-lysine (Sigma, USA) in a humidified atmosphere (5% CO_2_, 37 °C). HEK293 and *Piezo1* knockout (P1KO) HEK293 cells (GP9088, obtained from Beijing Jing Cheng Biotechnology Co., Ltd., China) were cultured in DMEM with 10% fetal bovine serum as a previous method [36]. The Piezo2 plasmid (#81,073) cloned from mouse DRG [37] and myc-KIF5B plasmid [38] (#127,617) were obtained from Addgene (USA). Full-length EndoA2-His and EndoA2-His (aa 1–308, aa 1–254) plasmids were obtained from Synbio Technologies (China). The plasmids were coexpressed in HEK293 cells. The cells were transfected with 1–2 μg plasmid using Lipofectamine 3000 reagent (Invitrogen, USA). The cells were used for subsequent experiments 24–48 h after transfection.

### Electrophysiological recordings

Patch-clamp recordings were performed using HEKA EPC-10 and Patchmaster v2 × 71 software as previously described [37, 39, 40]. Mechanically induced currents were recorded in small diameter (< 20 μm) and large diameter (> 30 μm) sensory neurons with glass pipettes (3–5 MΩ resistance) fabricated from borosilicate glass capillaries using a Sutter P-97 puller (Sutter Instruments, Novato, CA, USA). The external solution contained (in mmol/L): 127 NaCl, 3 KCl, 1 MgCl_2_, 10 HEPES, 2.5 CaCl_2_ and 10 glucose (pH adjusted to 7.3 with NaOH). The internal solution contained (in mmol/L): 133 CsCl, 10 HEPES, 5 EGTA, 1 CaCl_2_, 1 MgCl_2_, 4 MgATP and 0.4 Na_2_GTP (pH adjusted to 7.3 with CsOH). The membrane holding potential was − 80 mV. Mechanical stimulation was applied to neurons or cells using a fire-polished glass pipette (tip diameter 3–5 μm) positioned at an angle of 60°, and the probe displacement was advanced in increments of 1 μm.

### Extraction of plasma membrane proteins

For membrane protein preparation, samples were homogenized on ice with a plasma membrane protein extraction kit (Invent Biotechnologies, USA, SM-005). The efficiency of membrane protein extraction has been demonstrated in our previous work [41]. The detailed protocol was as follows: DRG tissues (L_4_–L_6_) were lysed with buffer A. The filter cartridge was capped and centrifuged at 16,000 × *g* for 30 s. The filter was discarded, and the pellet was resuspended and centrifuged at 700 × *g* for 1 min (the pellet contained the intact nuclei). The supernatant was transferred to a new tube and centrifuged for 10–30 min at 16,000 × *g*. The supernatant (the cytosolic fraction) was removed, and the pellet (the total membrane protein fraction including organelles and plasma membranes) was saved. The total membrane protein fraction was resuspended in buffer B and centrifuged at 7800 × *g* for 5 min. The pellet contained the organelle membrane proteins (in our study, cytoplasmic protein comprised the cytosolic fraction and organelle membrane fraction). The supernatant was carefully transferred to a fresh 2.0 ml microcentrifuge tube, and 1.6 ml ice-cold PBS was added. The sample was mixed a few times by inverting and centrifuged at 16,000 × *g* for 15–30 min. The supernatant was discarded, the pellet (isolated plasma membrane proteins) was saved, and the BCA method was used to determine the protein concentration. Protein samples of different fractions were denatured and prepared for immunoblotting.

### Surface protein biotinylation

Plasma membrane protein expression was detected by cell surface biotinylation using a Cell Surface Protein Isolation Kit (Pierce, USA, 89,881) according to the manufacturer’s instructions and a previously described method [24]. Briefly, cells were washed with PBS and biotinylated with Sulfo-NHS-SS-Biotin in PBS for 30 min at 4 °C. After quenching, the cells were lysed, and labeled proteins were isolated by incubation with NeutrAvidin Agarose beads for 60 min at room temperature (RT). After washing, the proteins were eluted by heating the beads for 5 min at 95 °C and prepared for immunoblotting.

### Western blotting

Lumbar spinal dorsal horn, dorsal root, L_4_–L_6_ DRGs, sciatic nerve tissues, cultured DRG neurons, and cultured HEK293 cells were lysed and homogenized in cold RIPA buffer. The protein samples were separated via gel electrophoresis (SDS‒PAGE) and transferred onto PVDF membranes. The membranes were placed in blocking buffer for 1 h at RT and incubated with primary antibodies against EndoA2 (rabbit, 1:1000, Bioss, China, bs-19747R; mouse, 1:200, Santa Cruz, USA, sc-365704), Piezo2 (rabbit, 1:1000, Novus, USA, NBP1-78,624; rabbit, 1:200, Alomone, Israel, APC-090), TACAN/Tmem120a (rabbit, 1:1000, Bioss, China, bs-19952R), Acid sensing ion channel 2 (ASIC2, rabbit, 1:1000, Bioss, China, bs-4915R), Acid sensing ion channel 3 (ASIC3, rabbit, 1:1000, Abcam, USA, ab190638), KIF5B (rabbit, 1:2000, Abcam, USA, ab167429), KIF5A (rabbit, 1:1000, Abcam, USA, ab5628), KIF17 (rabbit, 1:1000, Bioss, China, bs-3527R), KIF3A (goat, 1:100, Santa Cruz, USA, sc-18745), KIF3B (rabbit, 1:100, Santa Cruz, USA, sc-50456), transferrin receptor (TfR; mouse, 1:1000, Thermo Fisher Scientific, USA, 13-6800), β-actin (mouse, 1:2000, Affinity, China, T0022), β-tubulin (mouse, 1:2000, Arigo, China, ARG62347), GAPDH (mouse, 1:2000, Affinity, China, T0004), His-tag (mouse, 1:500, Santa Cruz, USA, sc-8036; rabbit, 1:1000, Cell Signaling Technology, USA, 12,698) or myc-tag (rabbit, 1:2000, Bioss, China, bs-23166R) overnight at 4 °C. Next, the membranes were incubated with an HRP-conjugated secondary antibody (1:10,000). Enhanced chemiluminescence (ECL) solution (Millipore, USA) was used to detect the immunocomplexes. Each band was quantified with computer-assisted imaging analysis software (Tanon Gis, China).

### Coimmunoprecipitation (Co-IP)

The Co-IP test was evaluated in accordance with previously provided instructions [24]. Transfected HEK293 cells or DRG tissues were lysed in cold Co-IP RIPA buffer. The lysates were centrifuged, and 5% of each supernatant was taken for the input sample. The remaining supernatants were incubated with 5–10 μg EndoA2 (mouse, Santa Cruz, USA, sc-365704), Piezo2 (rabbit, 1:200, Alomone, Israel, APC-090) or His (mouse, Santa Cruz, USA, sc-8036) antibody at 4 °C overnight and then with protein A/G beads (GE Healthcare, UK) at 4 °C for 4 h. The immunoprecipitated samples were denatured and prepared for immunoblotting. Immunoprecipitation was performed with antibodies against Piezo2 (rabbit, 1:1000, Novus, USA, NBP1-78,624; rabbit, 1:200, Alomone, Israel, APC-090), TACAN/Tmem120a (rabbit, 1:1000, Bioss, China, bs-19952R), ASIC2 (rabbit, 1:1000, Bioss, China, bs-4915R), ASIC3 (rabbit, 1:1000, Abcam, USA, ab190638), KIF5B (rabbit, 1:2000, Abcam, USA, ab167429), KIF5A (rabbit, 1:1000, Abcam, USA, ab5628), KIF17 (rabbit, 1:1000, Bioss, China, bs-3527R), KIF3A (goat, 1:100, Santa Cruz, USA, sc-18745), KIF3B (rabbit, 1:1000, Bioss, China, bs-17085R), His-tag (rabbit, 1:1000, Cell Signaling Technology, USA, 12,698) or myc-tag (rabbit, 1:2000, Bioss, China, bs-23166R). The precipitant was washed, denatured and prepared for immunoblotting.

### Proximity ligation assay (PLA)

PLA was performed using Duolink reagents (Sigma-Aldrich, USA, DUO92101) on cultured DRG neurons from L_4_–L_6_ of mice [42]. The isolated DRG neurons were cultured according to the above method. After 2 d of culture, the cells were fixed with 4% paraformaldehyde (PFA) at RT for 15 min and permeabilized with 0.1% Triton X-100 for 30 min. PLA was performed according to the Duolink PLA Protocol of Sigma‒Aldrich to examine the interactions between proteins. Briefly, cells on coverslips were blocked with Duolink blocking solution and incubated with a mixture of two primary antibodies (anti-EndoA2, mouse, 1:50, Santa Cruz, USA, sc-365704 and anti-Piezo2, rabbit, 1:200, Alomone, Israel, APC-090; anti-EndoA2, mouse, 1:50, Santa Cruz, USA, sc-365704 and anti-KIF5B, rabbit, 1:200, Abcam, USA, ab167429; anti-Piezo2, rabbit, 1:200, Alomone, Israel, APC-090 and anti-KIF5B, mouse, 1:50, Santa Cruz, USA, sc-133184) overnight at 4 °C. After incubation, the coverslips were washed with wash buffer A and then incubated with secondary antibodies (anti-mouse MINUS probe and anti-rabbit PLUS probe) for 1 h at 37 °C. Coverslips were then washed with wash buffer A and incubated with PLA ligase in ligation buffer for 30 min at 37 °C. After incubation, coverslips were washed with wash buffer A, incubated with polymerase in amplification buffer for 100 min at 37 °C, and then washed with wash buffer B for 1 min. Coverslips were mounted in mounting medium containing DAPI, and images were captured with a microscope. Negative control testing was conducted by omitting the primary antibody.

### Quantitative real-time polymerase chain reaction (qPCR)

Total RNA was extracted from the mouse L_4_–L_6_ DRGs with Trizol reagent (Invitrogen, USA). Reverse transcription was performed with M-MLV reverse transcriptase (Promega, USA) according to the manufacturer’s protocol. Primer sequences of *endophilin A1-A3, endophilin B1, endophilin B2* and *GAPDH* for PCRs are listed in Additional file 1: Table S2. Quantitative real-time PCR was performed with SYBR Green qPCR SuperMix (Invitrogen, USA) and an ABI PRISM7500 Sequence Detection System. The reactions were set up based on the manufacturer’s protocol. The PCR conditions were incubation at 95 °C for 3 min followed by 40 cycles of thermal cycling (10 s at 95 °C, 20 s at 58 °C, and 10 s at 72 °C). The relative expression ratio of mRNA was quantified via the $2^{-{{{\triangle}{\triangle}}\text{Ct}}}$ method.

### Immunohistochemistry

Mice were perfused with 4% PFA. The lumbar spinal dorsal cord, L_4_–L_6_ DRGs, dorsal root and sciatic nerve of mice were dissected and postfixed in 4% PFA for 1 h. Human thoracic segment DRGs, dorsal roots and sciatic nerves were collected from 5 patient donors at Sun Yat-sen University Cancer Center, and informed written consent from all participants or next of kin was obtained prior to the research and approved by the Research Ethics Committee of Sun Yat-sen University Cancer Center (SL-G2021-099-02). Each donor was undergoing surgery for disease treatment wherein the DRGs, dorsal roots and sciatic nerves were discarded as the standard of care. The DRGs, dorsal roots and sciatic nerves were immediately postfixed in 4% PFA overnight. Next, the tissues were dehydrated in 30% sucrose and embedded for cryostat sectioning. The cryostat sections were incubated with primary antibodies against EndoA2 (rabbit, 1:200, Bioss, China, bs-1974R; mouse, 1:50, Santa Cruz, USA, sc-365704), Piezo2 (rabbit, 1:200, Novus, USA, NBP1-78,624; rabbit, 1:200, Alomone, Israel, APC-090), IB4 (1:50, Sigma, USA, L2895), calcitonin gene-related peptide (CGRP; mouse, 1:200, Abcam, USA, ab81887), neurofilament-200 (NF200; mouse, 1:200, Sigma, USA, N0142), KIF5B (rabbit, 1:200, Abcam, USA, ab167429; mouse, 1:50, Santa Cruz, USA, sc-133184) or Flag (rabbit, 1:200, Cell Signaling Technology, USA, 14,793) at 4 °C overnight and then incubated with secondary antibodies (1:400) for 1 h at RT. Colocalization of antibodies from the same source was performed as follows: tissue sections were blocked, and primary antibody was added to the first target protein overnight at 4 °C. The cells were washed with PBS 3–5 times, and then the corresponding secondary antibody was added and incubated for 1 h. The mixture of primary and secondary antibodies of the second target protein was prepared and incubated on a shaker for 1 h. Then, an appropriate amount of IgG was added for 40 min. A mixture of primary and secondary antibodies was added for the second target protein. The cells were incubated for 1 h, washed with PBS 3–5 times, and then photographed under a fluorescence microscope. Three-dimensional superresolution images were captured using a three-dimensional structured illumination microscope with the N-SIM System (Nikon, Japan), and images were postprocessed with Nikon NIS-Elements software. To confirm the specificity of the Piezo2 antibody, blocking experiments were conducted in DRG sections using a mixture of anti-Piezo2 antibody and immunizing blocking peptide (10 times the molar concentration of the antibody, Alomone, Israel, BLP-PC090 and Novus, USA, NBP1-78624PEP) based on an immunizing peptide blocking protocol (https://www.abcam.com/protocols/blocking-with-immunizing-peptide-protocol-peptide-competition).

### Quantification of immunostained membrane Piezo2

DRG neurons from L_4_–L_6_ of mice were cultured for 2 d. Following fixation, DRG coverslips were treated with primary antibodies against Piezo2 (rabbit, 1:200, Novus, USA, NBP1-78,624) and NF200 (mouse, 1:200, Sigma, USA, N0142) and the appropriate secondary antibodies. Coverslips were then imaged using a confocal microscope. Using ImageJ software, profile plots of each cell were made spanning the cell diameter for Piezo2 immunoreactivity, and a background reading was also taken. An average of the signal intensity was then taken for the portion of the plot relating to the membrane and that relating to the cytoplasm (avoiding the nucleus). Only signal intensities greater than background were used. The ratio of membrane to cytoplasm was calculated, and those cells with a ratio greater than 1.5 times were defined as Pizeo2 membrane positive.

### Microinjection of adeno-associated virus (AAV) into the DRG

According to a previous study [43], the backs of the mice were shaved. The mice were anesthetized with isoflurane that was delivered in oxygen (4% for induction and 1.5–2.0% for maintenance) and an incision was made approximately 3 cm into the skin on the left side of the dorsal midline from the upper iliac crest. The superficial muscle fascia was incised, and the paravertebral muscles were separated by sharp and blunt dissection to expose the vertebrae of the fourth lumbar vertebra (L_4_) to L_5_. A retractor was used to maintain exposure. A rongeur was used to slightly enlarge the intervertebral foramen to expose the distal third of the L_4_ and L_5_ DRGs. While exposure performed this way initially produces local bleeding, this stopped promptly in all animals without any specific intervention. The glass microcapillary was inserted directly into the DRGs (L_4_ and L_5_). A unilateral injection (left side) was performed to minimize the time and tissue damage related to the operation. The AAV (serotype 5, genome copies: 2.0 × 10^12^ vg/ml) of rAAV-Syn-DIO-EndoA2-2A-enhanced green fluorescent protein (EGFP), rAAV-Syn-DIO-EndoA2-SH3-domain-Flag, rAAV-U6-KIF5B-shRNA-Syn-EGFP (sequence: GCATATGGACAAACATCATCT), rAAV-Syn-KIF5B-Flag and rAAV-U6-EndoA2-shRNA-Syn-mcherry (sequence: GCCTTGACTTTGACTACAAGA) were obtained from BrainVTA Biotechnology (China), and AAV solution (2 µl) was injected into the DRG through a glass micropipette. After microinjection, the inserted glass microcapillary was removed from the DRG, the skin was sutured with 3–0 silk, and the mice were kept under a heated light until recovery. Three weeks later, these mice were used for all experiments.

### Tamoxifen (TAM) injection

The TAM injection test was conducted following a previous description [19]. Fifteen milligrams of TAM (Sigma, USA) were dissolved in 1 ml of 100% corn oil and made fresh daily before use. The mice received 150 mg/kg TAM intraperitoneal injection (i.p.) for 5 d. Each mouse was weighed before the injection to standardize weight differences. Cotton, back tape, punctate, dynamic, tail clip, pinprick, Hargreaves, tail flick and cooling behavioral assays were performed on mice 7–21 d after TAM injections, and all mice that were tested for mechanical sensitivity were also tested for thermal sensitivity to control for any issues of general health. Before TAM injections, *EndoA2*^*fl/fl*^ × *AvCreERT2* mice were healthy and viable with no differences compared to WT littermate controls in various somatosensory assays.

### Antibody validation

For anti-EndoA2 see PMID: 30,061,681, 31,138,815 and was also validated in this paper; For anti-Piezo2 see PMID: 34,335,288, 35,198,872 and was also validated in this paper; For anti-TACAN/Tmem120a see PMID: 35,353,345; For anti-ASIC2 see PMID: 31,903,118; For anti-ASIC3 see PMID: 33,874,962; For anti-KIF5B, anti-KIF5A, anti-KIF17, anti-KIF3A, anti-KIF3B, anti-TfR, anti-β-actin, anti-β-tubulin, anti-GAPDH, anti-His-tag, anti-Myc-tag, anti-CGRP, isolectin B4 (IB4)-FITC, anti-NF200, anti-Flag see our previous publications PMID: 33,658,516, 31,446,225, 29,588,412, 33,433,144 and 35,353,345. The validation data for all antibodies can be found on the manufacturer's website.

### Animals/monkeys

Four adult male cynomolgus monkeys (Macaca fascicularis, 7.4–9.9 kg, 7–10 years old) were obtained from Guangzhou Xiangguan Biotechnology Co. Ltd. (Guangzhou, China) and maintained in a nonhuman primates experimental facility accredited by the Association for Assessment and Accreditation of Laboratory Animal Care International. Animals were individually housed in temperature- and humidity-controlled rooms maintained in a 12-h/12-h light/dark cycle. All monkeys had been previously acclimated before the behavioral assay. All animal care and experimental procedures were conducted in accordance with the Guide for the Care and Use of Laboratory Animals standards adopted by the National Institutes of Health (NIH) and approved by the Laboratory Animal Ethics Committee of the Institute of Zoology, Guangdong Academy of Sciences (G2Z20220710).

### Behavioural tests in monkeys

To explore the translational potential of our research, we conducted experiments involving monkeys due to their close phylogenetic relationship with humans. The behavioral tests in monkeys were carried out as previously outlined [42, 44]. The monkeys acclimated to the restraint chair for 1 h a day for a week before the behavioral tests. Behavioral tests were performed by an experimenter who was blinded to the drug being administered. EndoA2 siRNA infusion is achieved by intrathecal injection (i.t.). We first validated siRNA with monkey-derived Vero cells and then methylated and cholesterol-modified siRNA for in vivo i.t. (400 µl, sequence: AACCAGATTGACGAGAACT). Further behavioral tests were performed at 2 d, 4 d, and 10 d after siRNA injection. CFA (500 µl) was injected into the plantar surface of one hindpaw of the monkeys to produce inflammatory pain.

#### Von Frey test

Monkeys were adapted to a monkey restraint chair for 1 h with their feet kept free. Von Frey filaments with progressively increasing force were applied to the hind paw of monkeys. During the test, each filament was applied to the monkey’s hind paws 5 times, with a minimum interval of 10 min. The minimum filament force that caused paw withdrawal more than 3 times during the test was defined as the mechanical threshold. For the CFA-injected monkeys, the von Frey test was performed on the 2nd and 4th days after injection.

#### Dynamic mechanical test

Monkeys were adapted to a monkey restraint chair for 1 h. The outside of the rear paw was gently stroked from heel to toe with a 5/0 brush. The criterion for the response score was as follows: 0 = no response; 1 = very short, fast movement/lifting of the paw; 2 = sustained lifting of the paw lasting for more than 2 s or strong lifting of the paw; and 3 = flinching or flicking of the affected paw. Each monkey underwent 3 tests, with a minimum interval of 10 min between each test. The average score from the 3 tests was defined as the dynamic score.

#### Tail-flick test

The hair on the lower third of the tail was shaved, and the monkeys were adapted to the monkey cage for 2 d. The monkey was placed in a restraint chair with its tail outside the chair. The lower third of the tail was immersed in a water bath at 46 °C and 50 °C in turn (10 min apart, 3 times for each temperature) and the skin of the tail was dried every time. Next, the time to vigorous extraction of the tail from the water was measured.

#### Cotton test

First, cotton was pulled at the top of a medical cotton swab to 3 times its original size. The center of the monkey’s feet was gently touched from the bottom up with cotton, and the reaction of their feet was observed. A paw withdrawal motion in response to a stroke of the swab underneath the monkey paw was scored. Five sweeps were performed with at least 10 s between each. The number of withdrawals out of 5 trials was counted and reported as the percentage of withdrawal for each monkey.

#### Foot tape test

Before the experiment, the monkeys were trained to adapt to being grasped by their feet. In the experiment, a baffle was used to block the monkey’s view, and while grasping the monkey’s feet, transparent tape was randomly attached to the sole of one of the monkey’s feet. The transparent tape used in the experiment was cut to a size of 3 cm × 3 cm. The time was recorded from releasing the monkey’s foot to their discovery of the tape, with an upper limit of 5 min. The longer it takes for the monkeys to find the tape, the less sensitive they are to touch.

### Statistical analysis

The gray values of the western blotting bands were quantified by Tanon Gis software. The gray value of the images and colocalization were quantified by ImageJ 1.51j8 software. All data are expressed as the mean ± SEM. Statistical analyses were performed using two-tailed independent Student’s* t* test, One-way ANOVA followed by Tukey’s multiple comparisons test, and Two-way ANOVA followed by Bonferroni's multiple comparisons test, all conducted through GraphPad Prism 6 software. The threshold for statistical significance was *P* < 0.05.

## Results

### EndoA2 is mainly expressed in NF200-positive (NF200^+^) medium-to-large diameter sensory neurons

**Fig. 1:**
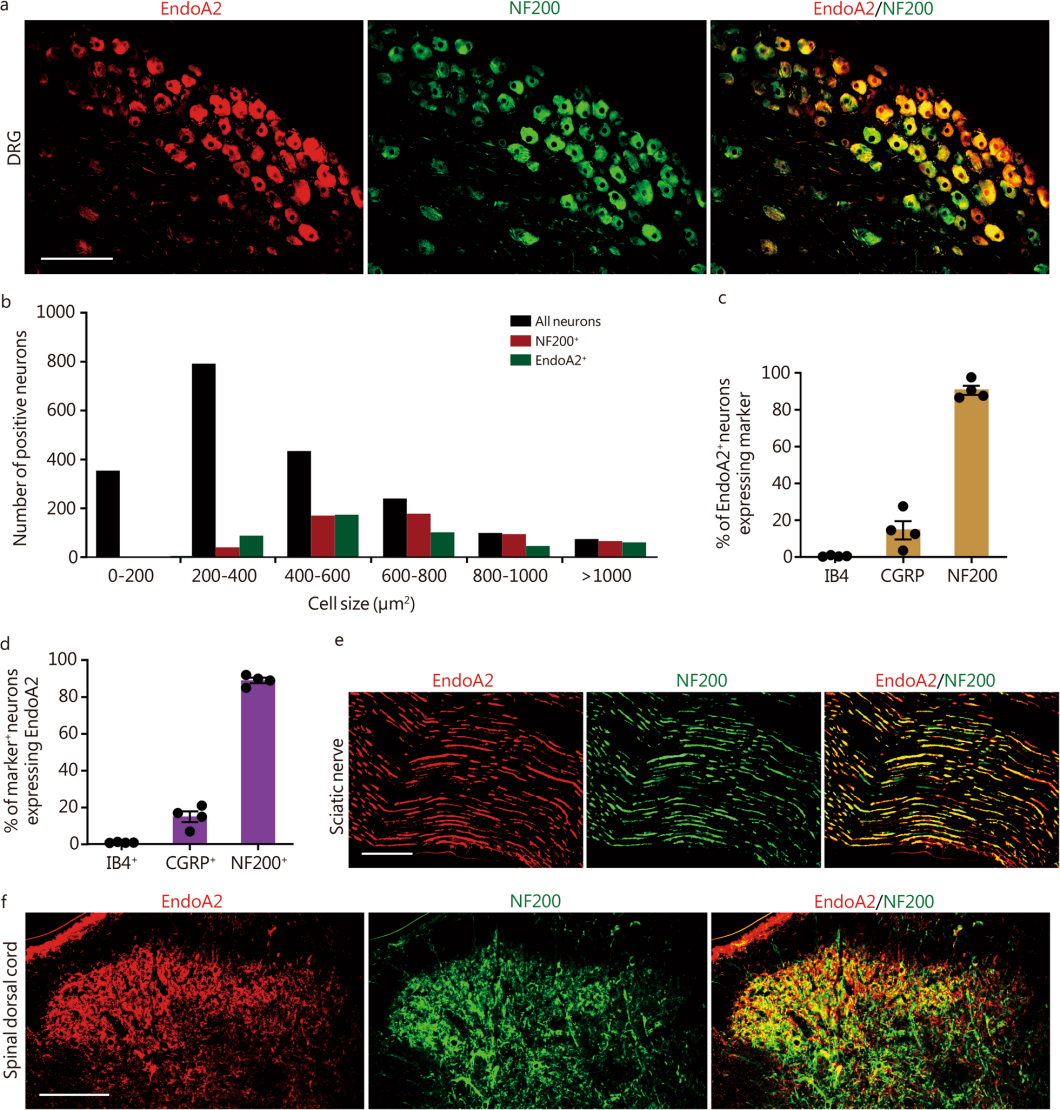
EndoA2 is mainly expressed in NF200-positive (NF200^+^) medium-to-large diameter sensory neurons. **a** Colocalization of EndoA2 with NF200 in DRG sections. Scale bar = 200 μm. **b** Size frequency distribution of EndoA2-positive (EndoA2^+^), NF200^+^ and total neurons in the DRG sections. A total of 1968 neurons from 4 mice were analyzed. **c** The percentage of EndoA2^+^ DRG neurons expressing IB4, CGRP and NF200. *n* = 4 mice per group. **d** The percentage of IB4^+^, CGRP^+^, and NF200^+^ DRG neurons expressing EndoA2. *n* = 4 mice per group. Double immunostaining of EndoA2 with NF200 in the sciatic nerve (**e**) and lumbar spinal dorsal cord (**f**) of mice. Scale bar = 100 μm (**e**) and 200 μm (**f**). EndoA2 endophilin A2, NF200 neurofilament-200, DRG dorsal root ganglion, IB4 isolectin B4, CGRP calcitonin gene-related peptide

Endophilins are a family of evolutionarily conserved proteins that are well documented in the endocytosis pathway [45]. To determine whether DRGs (L_4_–L_6_) express different endophilins, we first used qPCR to analyze 5 endophilins in DRG tissues. The results showed that all endophilin mRNAs were present in the DRGs of mice, and the expression abundance of *EndoA2* was the highest (Additional file 2: Fig. S1b). Importantly, the mRNA of *EndoA2* was increased in the L_4_–L_6_ DRGs of neuropathic pain mice induced by SNL (a neuropathic pain model) (Additional file 2: Fig. S1c). Then, we detected the expression profile of EndoA2 in the sensory nervous systems of mice by immunoblotting and found that EndoA2 was abundant in the L_4_–L_6_ DRGs (Additional file 2: Fig. S1d). EndoA2 was also present in the lumbar spinal dorsal cord, the dorsal root and the sciatic nerve (Additional file 2: Fig. S1d), suggesting that EndoA2 synthesized in the somata of DRG neurons is transported to their central and peripheral terminals. Immunostaining revealed that EndoA2 was mainly present in NF200^+^ DRG neurons (Fig. 1a), while a small amount was present in IB4-positive (IB4^+^) and CGRP-positive (CGRP^+^) DRG neurons (Additional file 2: Fig. S1e). Size frequency analysis revealed that overlapping distribution patterns of EndoA2-positive (EndoA2^+^) and NF200^+^ neurons were both present in medium-to-large neurons (Fig. 1b). Colocalization analysis showed that 91%, 15% and 1% of EndoA2^+^ neurons contained NF200, CGRP and IB4, respectively (Fig. 1c). Additionally, 89% of NF200^+^ neurons, 15% of CGRP^+^ neurons, and 1% of IB4^+^ neurons expressed EndoA2 (Fig. 1d). EndoA2 was also expressed in NF200^+^ A fibers rather than IB4^+^ or CGRP^+^ C fibers in the sciatic nerve and lumbar spinal dorsal cord (Fig. 1e, f; Additional file 2: Fig. S1f). Thus, EndoA2 is primarily expressed in NF200^+^ medium-to-large diameter sensory neurons.

### Loss of EndoA2 in sensory neurons inhibits touch and mechanical allodynia

**Fig. 2:**
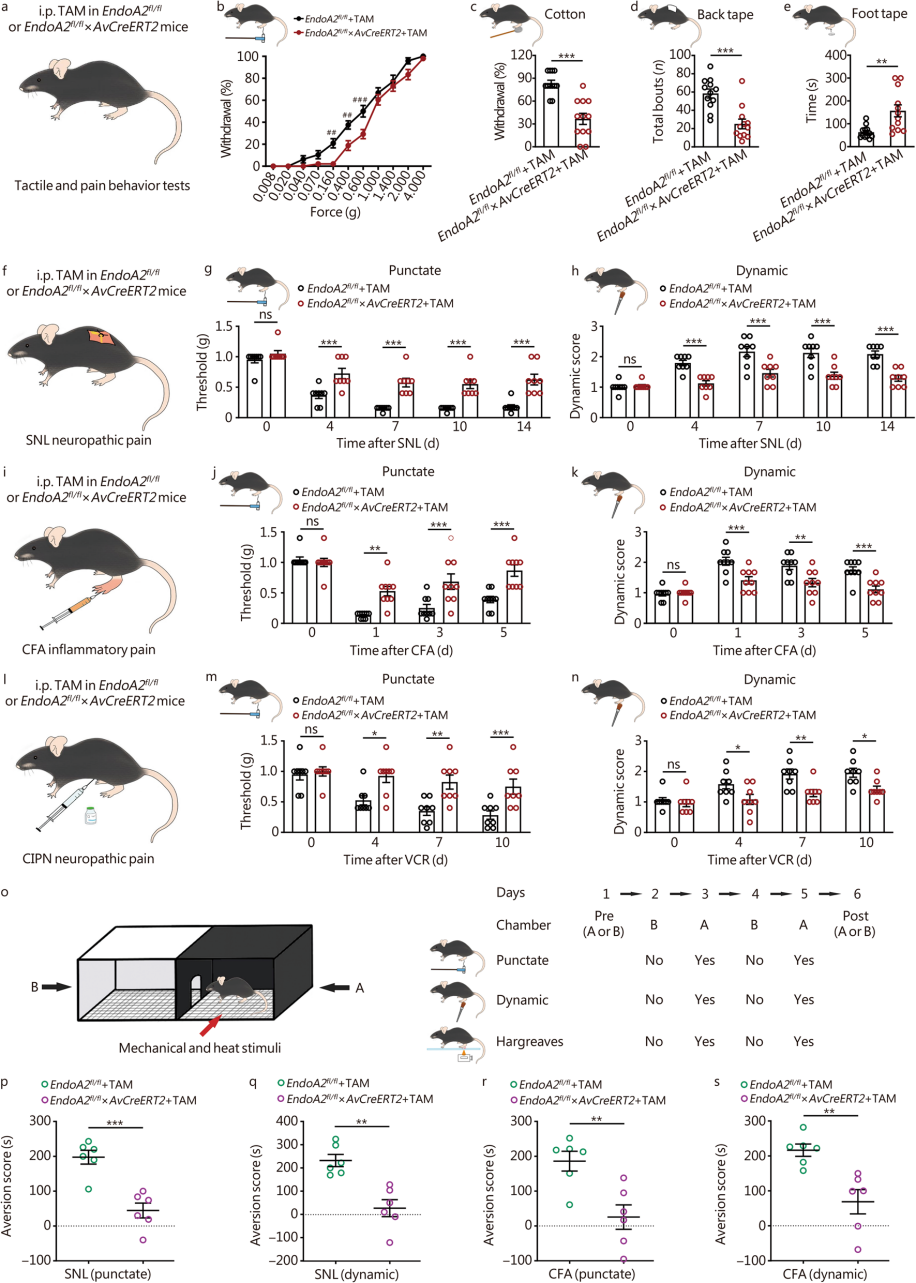
Loss of EndoA2 in sensory neurons inhibits touch and mechanical allodynia. **a** Tamoxifen (TAM)-inducible *EndoA2* knockout in sensory neurons was generated by crossing *EndoA2*^*fl/fl*^ mice to *AvCreERT2* mice. The *EndoA2*^*fl/fl*^ and *EndoA2*^*fl/fl*^ × *AvCreERT2* mice received 150 mg/kg TAM intraperitoneal injection (i.p.) for 5 d. Somatosensory behavioral assays were performed on mice 7–21 d after TAM injections. **b** The percentage of withdrawal for von Frey filaments under various forces in *EndoA2*^*fl/fl*^ and *EndoA2*^*fl/fl*^ × *AvCreERT2* mice after TAM injection. *n* = 12. ^##^*P* < 0.01, ^###^*P* < 0.001 compared with *EndoA2*^*fl/fl*^ group. The behaviors of *EndoA2*^*fl/fl*^ and *EndoA2*^*fl/fl*^ × *AvCreERT2* mice were evaluated by cotton (**c**), back tape (**d**) and foot tape (**e**) tests after TAM injection. *n* = 12. The effects of *EndoA2* knockout in sensory neurons on the punctate and dynamic mechanical allodynia induced by SNL (**f–h**), CFA (**i–k**) and VCR (**l–n**). *n* = 8–9. **o** Schematic of the CPA device and experimental design. The dark chamber is labeled A, and the bright chamber is labeled B. The amounts of time that mice spent in the dark A chamber during 15-min periods on Day 1 and Day 6 were measured. On Days 2–5, the mouse was placed in the indicated chamber for 20 min, with or without punctate, dynamic, and Hargreaves stimulation. **p** The effect of deletion of EndoA2 in DRG neurons on the CPA scores induced by punctate training in SNL mice (the CPA score was defined as the difference in the amount of time that the mice stayed in the dark A chamber before and after training: pre–post). *n* = 6. **q** The effect of deletion of EndoA2 in DRG neurons on the CPA scores induced by dynamic training in SNL mice. *n* = 6. The effect of deleting EndoA2 in DRG neurons on the CPA scores induced by punctate (**r**) and dynamic (**s**) training in CFA mice. *n* = 6. Two-tailed independent Student’s* t* test (**c**–**e**, **p**–**s**); Two-way ANOVA followed by Bonferroni’s multiple comparisons test (**b**, **g**, **h**, **j**, **k**, **m**, **n**). ^*^P < 0.05, ^**^P < 0.01, ^***^P < 0.001, ns non-significant. The error bars indicate the SEMs. EndoA2 endophilin A2, TAM tamoxifen, SNL spinal nerve ligation, CFA complete Freund’s adjuvant, VCR vincristine, CIPN chemotherapy-induced peripheral neuropathy

To determine the role of EndoA2 in sensory regulation, we generated a conditional, TAM-inducible *EndoA2* knockout mouse by crossing EndoA2-flox (*EndoA2*^*fl/fl*^) mice to *AvCreERT2* mice. The *AvCreERT2* mouse line allowed for TAM-induced activation of Cre recombinase under the Advillin promoter, leading to the specific deletion of the target gene in sensory neurons [19, 46]. The injection of TAM had no effect on the expression of EndoA2 in the DRG of *EndoA2*^*fl/fl*^ mice (Additional file 2: Fig. S2a, b). However, TAM administration significantly decreased the fluorescence intensity of EndoA2 and the proportion of EndoA2^+^ neurons in the DRG of *EndoA2*^*fl/fl*^ × *AvCreERT2* mice (Additional file 2: Fig. S2a, b). This indicates that TAM injection selectively eliminates the expression of EndoA2 in *EndoA2*^*fl/fl*^ × *AvCreERT2* mice but not in *EndoA2*^*fl/fl*^ mice. Although *AvCreERT2* ablates EndoA2 in most DRG neurons, a few remaining EndoA2^+^ neurons may still be intact, which is consistent with previous studies [19, 20]. Then, the experiments were performed in *EndoA2*^*fl/fl*^ and *EndoA2*^*fl/fl*^ × *AvCreERT2* mice after TAM injection (Fig. 2a). We first compared the mechanosensitivity of *EndoA2*^*fl/fl*^ and *EndoA2*^*fl/fl*^ × *AvCreERT2* mice by a range of punctate mechanical forces using von Frey filaments. Compared with *EndoA2*^*fl/fl*^ mice, *EndoA2*^*fl/fl*^ × *AvCreERT2* mice showed impaired responses at low filament strengths (0.160–0.600 g; Fig. 2b). Deletion of EndoA2 in DRG neurons also decreased mouse responses to innocuous touch stimulation induced by cotton (Fig. 2c), back tape (Fig. 2d) and foot tape (Fig. 2e). *EndoA2*^*fl/fl*^ × *AvCreERT2* mice had a normal withdrawal threshold to von Frey filament-evoked punctate mechanical stimulation (Additional file 2: Fig. S2c) and brush-evoked dynamic mechanical stimulation (Additional file 2: Fig. S2d). Deletion of EndoA2 in DRG neurons had no significant influence on the sensitivity of mice to intense mechanical stimulation in the tail clip (Additional file 2: Fig. S2e) or pinprick (Additional file 2: Fig. S2f) tests. Compared with *EndoA2*^*fl/fl*^ mice, *EndoA2*^*fl/fl*^ × *AvCreERT2* mice had comparable sensitivity to noxious heat stimulation in the Hargreaves test (Additional file 2: Fig. S2g) and the tail-flick (Additional file 2: Fig. S2h–j) tests. In addition, no difference in responses to the evaporative cooling test was detected between the *EndoA2*^*fl/fl*^ and *EndoA2*^*fl/fl*^ × *AvCreERT2* mice (Additional file 2: Fig. S2k). Furthermore, we investigated the role of EndoA2 in pathologic pain. Behavioral data showed that loss of EndoA2 in DRG neurons significantly relieved punctate mechanical allodynia and dynamic mechanical allodynia but not heat hyperalgesia induced by SNL (Fig. 2f–h, Additional file 2: Fig. S2l). Consistently, deletion of EndoA2 in sensory neurons alleviated punctate mechanical allodynia and dynamic mechanical allodynia but not heat hyperalgesia induced by CFA (an inflammatory pain model; Fig. 2i–k, Additional file 2: Fig. S2m) and VCR (a chemotherapeutic pain model; Fig. 2l–n). Thus, this suggests that EndoA2 mediates low-threshold touch perception and mechanical allodynia.

To assess the emotional and cognitive aspects of mechanical hypersensitivity, we performed a CPA assay (Fig. 2o). We measured the amounts of time mice spent in the dark A chamber on Day 1 (t1) of preconditioning and Day 6 (t2) of postconditioning, and the difference (∆t = t1 − t2) was used to evaluate the degree of CPA. We found that punctate mechanical (0.4 g; Fig. 2p, Additional file 2: Fig. S3a), dynamic mechanical (Fig. 2q, Additional file 2: Fig. S3b) and Hargreaves heat (cutoff time 10 s; Additional file 2: Fig. S3c, d) evoked prominent CPA in *EndoA2*^*fl/fl*^ mice with SNL treatment. Importantly, deletion of EndoA2 in DRG neurons largely abolished punctate (Fig. 2p, Additional file 2: Fig. S3a) and dynamic (Fig. 2q, Additional file 2: Fig. S3b) evoked CPA but did not alter Hargreaves (Additional file 2: Fig. S3c, d) induced CPA in SNL mice. Similarly, punctate mechanical (Fig. 2r, Additional file 2: Fig. S3e), dynamic mechanical (Fig. 2s, Additional file 2: Fig. S3f) and Hargreaves heat (Additional file 2: Fig. S3g, h) also induced significant CPA in *EndoA2*^*fl/fl*^ mice with CFA treatment. Mutation of EndoA2 in DRG neurons alleviated punctate (Fig. 2r, Additional file 2: Fig. S3e) and dynamic (Fig. 2s, Additional file 2: Fig. S3f) evoked CPA and had no effect on Hargreaves (Additional file 2: Fig. S3g, h) induced CPA in CFA mice. The punctate (Additional file 2: Fig. S3i, j) and dynamic (Additional file 2: Fig. S3k, l) induced CPA in VCR mice were also blocked after EndoA2 deficiency. Thus, EndoA2 may be involved in the processing of the affective and/or cognitive aspects of neuropathic mechanical pain.

### EndoA2 in NF200^+^ medium-to-large diameter sensory neurons is necessary for tactus and mechanical allodynia

**Fig. 3:**
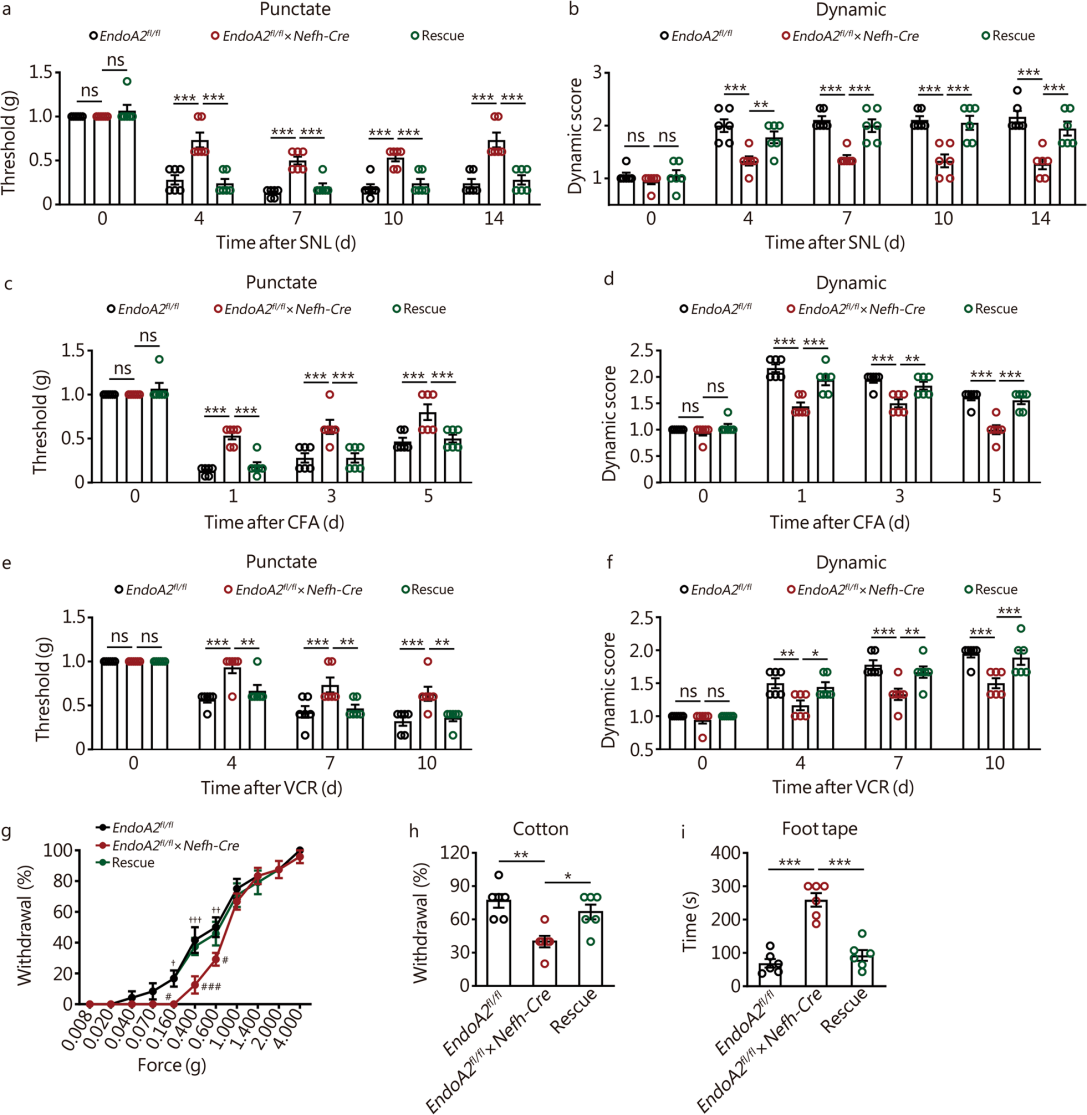
EndoA2 in NF200-positive (NF200^+^) medium-to-large diameter sensory neurons is necessary for tactus and mechanical allodynia. Conditional knockout and re-expression of *EndoA2* in NF200^+^ DRG neurons impaired and rescued SNL-induced punctate mechanical hypersensitivity (**a**) and dynamic mechanical hypersensitivity (**b**), respectively. *n* = 6. **c–f** Conditional knockout and re-expression of *EndoA2* in NF200^+^ DRG neurons impaired and rescued CFA- and VCR-induced punctate mechanical allodynia and dynamic mechanical allodynia, respectively. *n* = 6.** g** The percentage of withdrawal for von Frey filaments under a range of forces in* EndoA2*^*fl/fl*^,* EndoA2*^*fl/fl*^ ×* Nefh-Cre* and rescue mice.* n* = 6. ^†^*P* < 0.05, ^††^*P* < 0.01, ^†††^*P* < 0.001 indicates* EndoA2*^*fl/fl*^ vs.* EndoA2*^*fl/fl*^ ×* Nefh-Cre*; ^#^*P* < 0.05, ^###^*P* < 0.001 indicates* EndoA2*^*fl/fl*^ ×* Nefh-Cre* vs. rescue. The behaviors of* EndoA2*^*fl/fl*^,* EndoA2*^*fl/fl*^ ×* Nefh-Cre* and rescue mice were evaluated by cotton (**h**) and foot tape (**i**) tests.* n* = 6. One-way ANOVA followed by Tukey’s multiple comparisons test (**h**, **i**); Two-way ANOVA followed by Bonferroni’s multiple comparisons test (**a**–**g**). ^*^P < 0.05, ^**^P < 0.01, ^***^P < 0.001, ns non-significant. The error bars indicate the SEMs. EndoA2 endophilin A2, SNL spinal nerve ligation, CFA complete Freund’s adjuvant, VCR vincristine

To explore the specific role of EndoA2 expressed by NF200^+^ medium-to-large diameter neurons in pain regulation, we generated *EndoA2* conditional knockout mice by crossing *EndoA2*^*fl/fl*^ mice with *Nefh-Cre* mice (*EndoA2*^*fl/fl*^ × *Nefh-Cre*; *Nefh* is the gene of NF200; Additional file 2: Fig. S4a), leading to deletion of EndoA2 in NF200^+^ DRG neurons (Additional file 2: Fig. S4b–d). Notably, *EndoA2*^*fl/fl*^ × *Nefh-Cre* mice exhibited no changes in sensory neurons or their central innervations (Additional file 2: Fig. S4e–h). *EndoA2*^*fl/fl*^ × *Nefh-Cre* mice displayed impaired responses to low threshold innocuous and touch mechanical stimulation (Additional file 2: Fig. S3a–c) and had a normal withdrawal threshold to punctate (Additional file 2: Fig. S5a) and dynamic (Additional file 2: Fig. S5b) mechanical stimulation. Deletion of EndoA2 in NF200^+^ cells did not alter the responses to noxious mechanical (Additional file 2: Fig. S5c) and heat (Additional file 2: Fig. S5d) pain stimulation in mice. *EndoA2*^*fl/fl*^ × *Nefh-Cre* mice also showed normal evaporative cooling sensitivity (Additional file 2: Fig. S5e). Loss of EndoA2 in NF200^+^ neurons significantly remitted punctate (Fig. 3a) and dynamic (Fig. 3b) mechanical allodynia but not heat hyperalgesia (Additional file 2: Fig. S5f) induced by SNL. Consistently, the punctate (Fig. 3c) and dynamic (Fig. 3d) mechanical allodynia, but not heat hyperalgesia (Additional file 2: Fig. S5g), induced by CFA was alleviated in *EndoA2*^*fl/fl*^ × *Nefh-Cre* mice. Deletion of EndoA2 in NF200^+^ neurons also markedly mitigated chemotherapeutic drug VCR-evoked mechanical hypersensitivity (Fig. 3e, f). Therefore, *EndoA2* knockout in NF200^+^ cells results in touch perception and mechanical allodynia deficits in mice.

Since NF200 is also expressed in the brain [47] and spinal cord [48], we next determined the specific role of EndoA2 in NF200^+^ DRG neurons. To achieve this, we locally injected DRGs (L_4_ and L_5_) with AAV encoding EndoA2 under the control of the neuronal promoter Syn and the DIO Cre-on system (rAAV-Syn-DIO-EndoA2-2A-EGFP) in *EndoA2*^*fl/fl*^ × *Nefh-Cre* mice to rescue the expression of EndoA2 in NF200^+^ DRG neurons (rescue mice; Additional file 2: Fig. S5h). EGFP was observed in DRG NF200^+^ neurons of AAV-injected mice (Additional file 2: Fig. S5i), confirming transgene expression in NF200^+^ DRG neurons. The expression of EndoA2 was rescued in the DRG of *EndoA2*^*fl/fl*^ × *Nefh-Cre* mice after injection with AAV encoding EndoA2 solution (Additional file 2: Fig. S4b–d). The behavioral data showed that re-expression of EndoA2 rescued innocuous mechanical sensitivity in *EndoA2*^*fl/fl*^ × *Nefh-Cre* mice (Fig. 3g–i). EndoA2 rescue mice had a normal withdrawal threshold to punctate (Additional file 2: Fig. S5a) and dynamic (Additional file 2: Fig. S5b) mechanical stimulation. Rescuing the expression of EndoA2 did not change the response to noxious mechanical and heat stimulation (Additional file 2: Fig. S5c, d). Re-expression of EndoA2 also did not alter evaporative cooling sensitivity (Additional file 2: Fig. S5e). Importantly, SNL-, CFA- and VCR-induced punctate and dynamic mechanical allodynia were rescued in *EndoA2*^*fl/fl*^ × *Nefh-Cre* mice after re-expression of EndoA2 (Fig. 3a–f). Re-expression of EndoA2 did not change the heat hyperalgesia induced by SNL (Additional file 2: Fig. S5f) or CFA (Additional file 2: Fig. S5g) in *EndoA2*^*fl/fl*^ × *Nefh-Cre* mice. Thus, rescue of EndoA2 in NF200^+^ DRG neurons can restore touch sensitivity and mechanical allodynia in *EndoA2*^*fl/fl*^ × *Nefh-Cre* mice, indicating that the expression of EndoA2 in NF200^+^ DRG neurons is necessary for touch and mechanical allodynia.

To further determine the role of EndoA2 in small diameter DRG neurons, we crossed *EndoA2*^*fl/fl*^ mice with *Trpv1-Cre* mice (*EndoA2*^*fl/fl*^ × *Trpv1-Cre*) to delete the expression of EndoA2 in TRPV1-positive small diameter DRG neurons. The behavioral tests were undertaken on *EndoA2*^*fl/fl*^ × *Trpv1-Cre* mice. Compared with *EndoA2*^*fl/fl*^ mice, *EndoA2*^*fl/fl*^ × *Trpv1-Cre* mice showed comparable touch sensitivity (Additional file 2: Fig. S6a–c). *EndoA2*^*fl/fl*^ × *Trpv1-Cre* mice had a normal withdrawal threshold to punctate (Additional file 2: Fig. S6d) and dynamic (Additional file 2: Fig. S6e) stimulation. Deletion of EndoA2 in small-diameter DRG neurons did not alter the sensitivity response to pinprick (Additional file 2: Fig. S6f), Hargreaves (Additional file 2: Fig. S6g), tail flicking (Additional file 2: Fig. S6h–j) or cooling (Additional file 2: Fig. S6k) tests. Furthermore, we explored the effect of *EndoA2* knockout in small-diameter DRG neurons on pathologic pain. The behavioral data showed that *EndoA2*^*fl/fl*^ × *Trpv1-Cre* mice exhibited mechanical and thermal hypersensitivity similar to *EndoA2*^*fl/fl*^ mice after CFA treatment at different time points (Additional file 2: Fig. S6l–n). These data suggest that EndoA2 in small-diameter DRG neurons is not associated with touch and pain sensitivity in mice.

### EndoA2 interacts with Piezo2 in sensory neurons

**Fig. 4:**
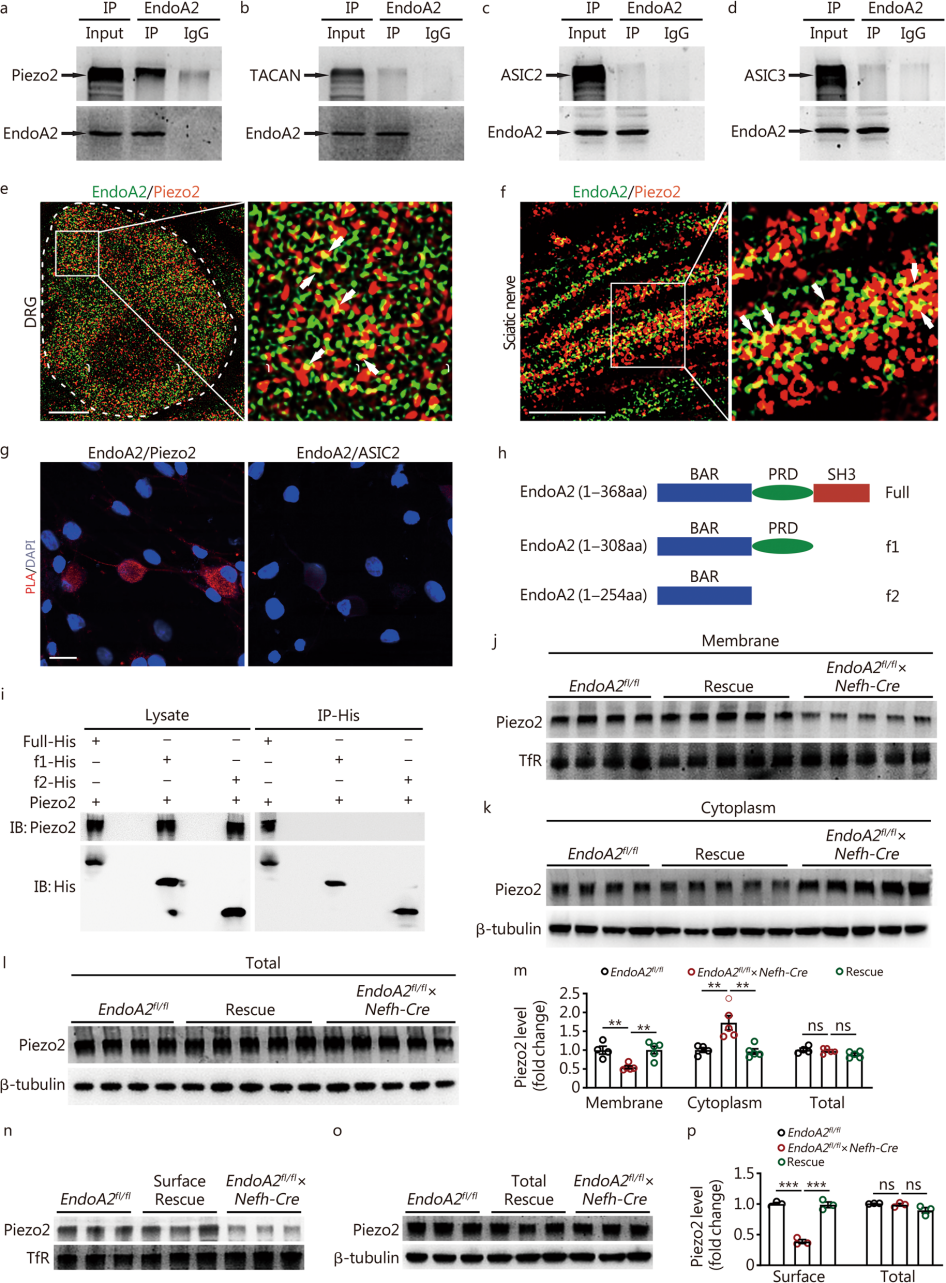
EndoA2 interacts with Piezo2 and regulates Piezo2 trafficking in sensory neurons. **a–d** Co-immunoprecipitation (Co-IP) results showed that EndoA2 potentially interacted with Piezo2 in DRGs. DRG lysates were immunoprecipitated with EndoA2 antibody and immunoblotted with Piezo2, TACAN, ASIC2, ASIC3 and EndoA2 antibody as indicated. This experiment was repeated 3 times. High-resolution images showing the colocalization of EndoA2 and Piezo2 in DRG neurons (**e**) and sciatic nerves (**f**) of mice. Arrows indicate colocalization. Scale bar = 5 μm. **g** Proximity ligation assay (PLA) showed positive signals of EndoA2/Piezo2 interaction in cultured DRG neurons, and staining was absent with incubation of EndoA2 and ASIC2 antibody (6 images from two repeats). Scale bar = 30 μm. **h** Diagram of full-length EndoA2 and EndoA2 mutant proteins. **i** Interaction between Piezo2 and EndoA2 mutants. The EndoA2 mutants were transiently coexpressed with Piezo2, and the cell lysates were immunoprecipitated with antibodies as indicated. This experiment was repeated 3 times. **j–l** Piezo2 expression in the DRG neuron membrane fraction (**j**), cytoplasmic fraction (**k**) and total lysate (**l**) from *EndoA2*^*fl/fl*^, *EndoA2*^*fl/fl*^ × *Nefh-Cre* and rescue mice. *n* = 4–5. **m** Statistical diagram of Piezo2 expression in the DRG neuron membrane fraction (**j**), cytoplasmic fraction (**k**) and total lysate (**l**) from *EndoA2*^*fl/fl*^, *EndoA2*^*fl/fl*^ × *Nefh-Cre* and rescue mice. *n* = 4–5. **n–p** Piezo2 surface levels were measured in cultured DRG neurons prepared from *EndoA2*^*fl/fl*^, *EndoA2*^*fl/fl*^ × *Nefh-Cre* and rescue mice using a surface biotinylation assay. *n* = 3 repeats per group. One-way ANOVA followed by Tukey’s multiple comparisons test (**m**, **n, o, p**). ^**^P < 0.01, ^***^P < 0.001, ns non-significant. The error bars indicate the SEMs. EndoA2 endophilin A2, DRG dorsal root ganglion, PLA proximity ligation assay, TfR transferrin receptor

To determine the molecular mechanism by which EndoA2 regulates touch and mechanical allodynia, we tested the interaction between EndoA2 and some channels (Piezo2, TACAN/Tmem120a, ASIC2 and ASIC3) expressed in sensory neurons and involved in the regulation of mechanical sensation by the Co-IP method [19, 49–52]. The data showed that EndoA2 had a potential interaction with the Piezo2 channel (Fig. 4a) and barely with the TACAN, ASIC2 or ASIC3 channels (Fig. 4b–d) in the DRG protein extract of mice. Furthermore, we performed high-resolution imaging by structured illumination microscopy (SIM) and found that (33.15 ± 4.21)% and (54.92 ± 5.69)% of EndoA2 was colocalized with Piezo2 in DRG neurons and sciatic nerves (Fig. 4e, f; Additional file 2: Fig. S7a), respectively. The colocalization rate of Piezo2 with EndoA2 in DRG neurons was (38.39 ± 2.86)%, and that in sciatic nerves was (38.68 ± 3.68)% (Fig. 4e, f; Additional file 2: Fig. S7a). The specificity of the Piezo2 antibodies was validated by loss of Piezo2 immunostaining in the DRG neurons of *Piezo2*^*CKO*^ mice and further confirmed by the absence of staining in the DRG after coincubation with blocking peptides (Additional file 2: Fig. S7b). To further confirm the potential EndoA2/Piezo2 interaction, we performed a PLA in dissociated L_4_–L_6_ DRG neurons [42]. PLA analysis revealed positive fluorescence signals on cell bodies and axons in cultured mouse DRG neurons after incubation with EndoA2 and Piezo2 primary antibodies, and staining was absent after incubation with EndoA2 and ASIC2 antibodies (Fig. 4g). These results indicate the close proximity of EndoA2 and Piezo2 and the possible interaction between these two molecules in DRG neurons. A study showed that EndoA2 consists of multiple domains for protein‒protein interactions: C-terminal Src homology 3 (SH3) domain, N-terminal Bin-amphiphysin-Rvs (BAR) domain and proline-rich domain (PRD) [53]. To determine how EndoA2 interacts with Piezo2, we constructed EndoA2 deletion mutants, which failed to induce the function of the SH3 domain (f1) and SH3 and PRD domains (f2) (Fig. 4h). Co-IP analysis showed that EndoA2-full interacted with Piezo2, however, EndoA2-f1 and EndoA2-f2 exhibited no association with Piezo2 (Fig. 4i). Thus, these results indicate that EndoA2 interacts with Piezo2 through the C-terminal SH3 domain.

### EndoA2 regulates Piezo2 trafficking in sensory neurons

**Fig. 5:**
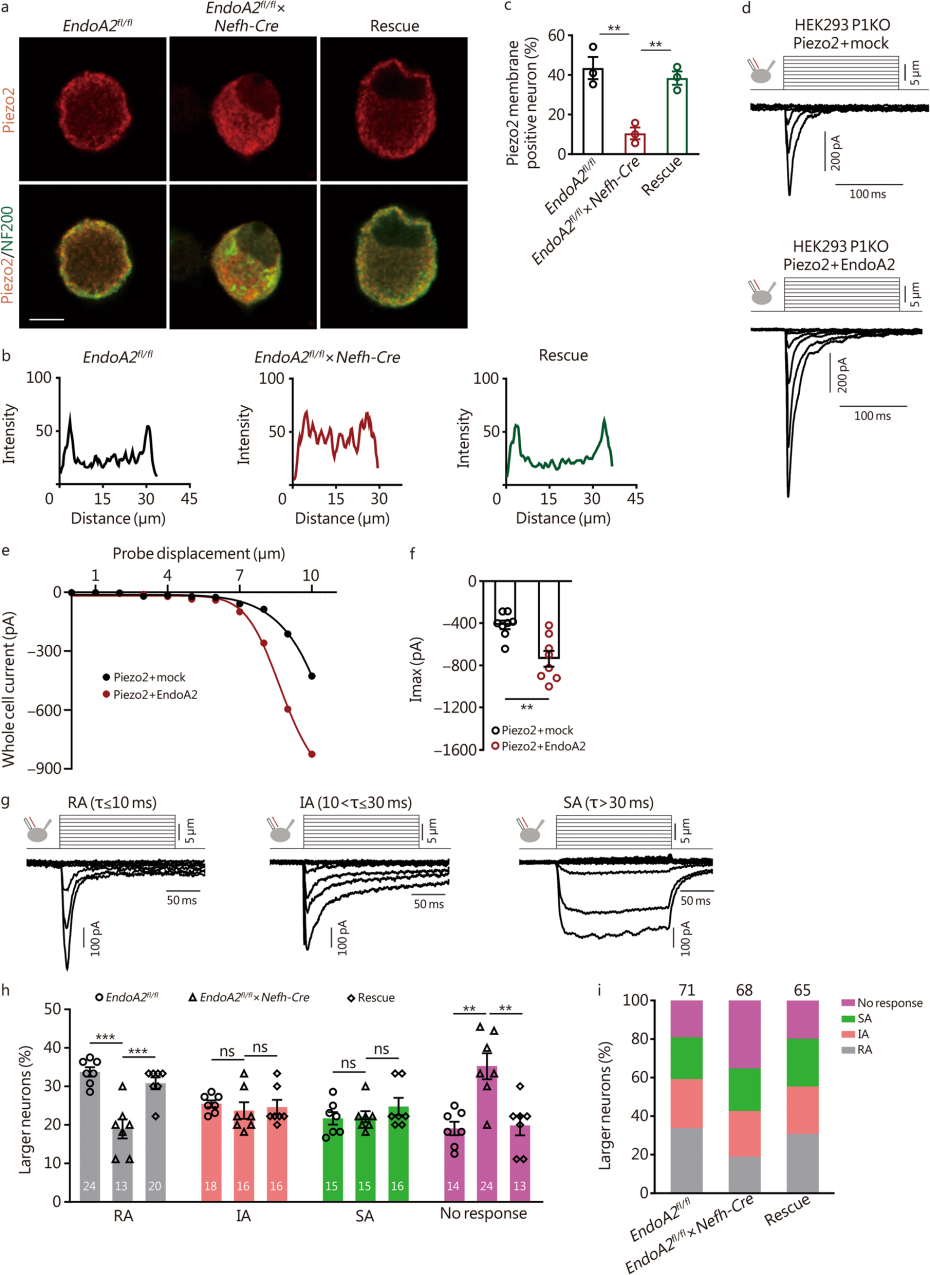
EndoA2 increases Piezo2-mediated mechanically activated (MA) currents. **a** Representative images showing Piezo2 membrane and cytoplasm staining in DRG neurons from *EndoA2*^*fl/fl*^, *EndoA2*^*fl/fl*^ × *Nefh-Cre* and rescue mice. Scale bar = 15 μm. **b** Profile plots were used to define membrane staining. A ratio of membrane to cytoplasm was calculated, and those cells with a ratio greater than 1.5 times were defined as Piezo2 membrane positive. **c** The proportion of Piezo2 membrane positive neurons in NF200^+^ cultured DRG neurons from *EndoA2*^*fl/fl*^, *EndoA2*^*fl/fl*^ × *Nefh-Cre* and rescue mice. *n* = 3 repeats. Eighty, 57 and 74 NF200^+^ neurons were analyzed in the *EndoA2*^*fl/fl*^, *EndoA2*^*fl/fl*^ × *Nefh-Cre* and rescue groups, respectively. **d, e** MA inward currents recorded in HEK293 *Piezo1* knockout (P1KO) cells transiently transfected with Piezo2 + mock and Piezo2 + EndoA2. Cells were subjected to a series of mechanical steps of 1-μm movements of a stimulation pipette (inset illustration, arrow) in the whole-cell patch configuration at a holding potential of − 80 mV. **f** The maximal amplitude of MA inward currents elicited at a holding potential of − 80 mV in Piezo2 + mock- and Piezo2 + EndoA2-transfected HEK293 P1KO cells. *n* = 8. **g** The representative traces of RA, IA, and SA MA inward currents expressed in DRG neurons are characterized by distinct inactivation kinetics. Neurons were subjected to a series of mechanical steps in 1-μm increments at a holding potential of − 80 mV. Frequency histograms indicating the proportion of larger DRG neurons (> 30 μm) from *EndoA2*^*fl/fl*^, *EndoA2*^*fl/fl*^ × *Nefh-Cre* and rescue mice that respond to mechanical stimulation, with MA currents characterized by their inactivation kinetics. The proportion of neurons from 7 separate experiments (**h**) (*n* = 7 to 13 neurons per condition and per experiment) or the proportion from all neurons pooled from all 7 experiments (**i**). *n* = 71, 68 and 65 neurons in the *EndoA2*^*fl/fl*^, *EndoA2*^*fl/fl*^ × *Nefh-Cre* and rescue groups, respectively. One-way ANOVA followed by Tukey’s multiple comparisons test (**c**,** h**); Two-tailed independent Student’s* t* test (**f**). ^**^P < 0.01, ^***^P < 0.001, ns non-significant. The error bars indicate the SEMs. EndoA2 endophilin A2, NF200 neurofilament-200, P1KO Piezo1 knockout, RA rapidly adapting, IA intermediately adapting, SA slowly adapting

Next, we examined the role of EndoA2 in regulating the membrane, cytoplasm, and total expression of Piezo2 in the DRGs of mice. The data showed that compared with *EndoA2*^*fl/fl*^ mice, the membrane expression of Piezo2 was decreased in the DRGs of *EndoA2*^*fl/fl*^ × *Nefh-Cre* mice (Fig. 4j, m). Interestingly, conditional knockout of *EndoA2* in NF200^+^ neurons increased the expression of Piezo2 in the cytoplasm (Fig. 4k, m) but did not change the total (Fig. 4l, m) expression of Piezo2 in the DRGs of mice. Importantly, re-expression of EndoA2 in DRG neurons normalized the membrane and cytoplasm expression of Piezo2 but did not change the total expression of Piezo2 in *EndoA2*^*fl/fl*^ × *Nefh-Cre* mice (Fig. 4j–m). The trafficking ratio of Piezo2 significantly decreased in the DRGs of *EndoA2*^*fl/fl*^ × *Nefh-Cre* mice and returned to normal after re-expression of EndoA2 (Additional file 2: Fig. S7c). Surface biotinylation analysis revealed a reduction in Piezo2 surface expression in DRG neurons of *EndoA2*^*fl/fl*^ × *Nefh-Cre* mice, and re-expression of EndoA2 rescued the surface expression of Piezo2 (Fig. 4n–p). We assessed the expression of Piezo2 in cultured DRG neurons and found clear membrane staining of Piezo2 in DRG neurons from *EndoA2*^*fl/fl*^ mice that was lost following deletion of EndoA2, and re-expression of EndoA2 rescued the surface expression of Piezo2 (Fig. 5a). Profile plots showed the intensity of Piezo2 immunoreactivity across the cell body, and the ratio of membrane to cytoplasm was calculated. Cells with a ratio greater than 1.5 times were defined as Piezo2 membrane positive (Fig. 5b). The proportion of Piezo2 membrane positive neurons was decreased in NF200^+^ DRG neurons of *EndoA2*^*fl/fl*^ × *Nefh-Cre* mice compared to *EndoA2*^*fl/fl*^ mice and returned to normal after re-expression of EndoA2 (Fig. 5c). Consistently, in the sciatic nerves of *EndoA2*^*fl/fl*^ × *Nefh-Cre* mice, Piezo2 expression decreased in the membrane (Additional file 2: Fig. S7d) and increased in the cytoplasm (Additional file 2: Fig. S7e), but Piezo2 expression remained unchanged in the total amount (Additional file 2: Fig. S7f). Notably, the membrane and total expression of Piezo2 were not altered in DRG tissues of *EndoA2*^*fl/fl*^ × *Trpv1-Cre* mice (Additional file 2: Fig. S7g, h). Thus, EndoA2 participates in the regulation of Piezo2 membrane trafficking in NF200^+^ medium-to-large diameter sensory neurons. Research shows that broad and constitutive Piezo2 loss in DRG neurons results in proprioceptive defects in mice [54]. Thus, we detected the effects of EndoA2 on proprioception in mice. We evaluated proprioception in mice by measuring motor coordination in a rotarod experiment. Compared with *EndoA2*^*fl/fl*^ mice, motor coordination was decreased in *EndoA2*^*fl/fl*^ × *Nefh-Cre* mice (Additional file 2: Fig. S7i), suggesting that EndoA2 is involved in regulating proprioception by reducing the membrane trafficking of Piezo2 in DRG neurons.

### EndoA2 modulates Piezo2-evoked mechanically sensitive currents

Furthermore, we tested whether the activity of Piezo2 could be modulated by EndoA2. We elicited Piezo2-mediated currents by poking transiently transfected HEK293 cells with P1KO with a blunt glass probe [37, 55, 56]. We cotransfected recombinant Piezo2 and EndoA2 together in P1KO HEK293 cells and found that EndoA2 robustly increased the Piezo2-mediated MA currents (Fig. 5d–f). Although physiological mechanotransduction occurs at the nerve terminals, many MA channels are also expressed in and can be recorded from DRG cell bodies [57]. Furthermore, we detected the role of EndoA2 in the MA currents of DRG neurons. According to inactivation kinetics, MA currents are divided into three categories (Fig. 5g): rapidly adapting (RA, τ ≤ 10 ms), intermediately adapting (IA, 10 < τ ≤ 30 ms) and slowly adapting (SA, τ > 30 ms) currents [19, 37, 39]. Piezo2 is an RA MA ion channel expressed in DRG neurons of all sizes [37, 58]. Since EndoA2 is primarily expressed in large-diameter DRG neurons, we first recorded MA currents in large-diameter DRG neurons [59] (> 30 μm) of *EndoA2*^*fl/fl*^ and *EndoA2*^*fl/fl*^ × *Nefh-Cre* mice. DRGs from *EndoA2*^*fl/fl*^ × *Nefh-Cre* mice had specifically and significantly fewer neurons with RA MA currents compared to controls and a corresponding increase in the proportion of mechanically nonresponsive neurons (Fig. 5h, i). There were no significant effects on IA or SA MA currents (Fig. 5h, i). Re-expression of EndoA2 normalized the proportion of RA MA currents and no-response neurons [Fig. 5h, i; *EndoA2*^*fl/fl*^ = (33.99 ± 3.62) μm vs. *EndoA2*^*fl/fl*^ × *Nefh-Cre* = (33.38 ± 2.96) μm vs. rescue = (32.80 ± 2.50) μm]. Furthermore, we recorded MA currents in small-diameter DRG neurons [59] (< 20 μm). Compared with those from *EndoA2*^*fl/fl*^ mice, small-diameter DRG neurons from *EndoA2*^*fl/fl*^ × *Nefh-Cre* mice had normal RA, IA and SA MA currents, and the proportion of mechanically nonresponsive neurons was also comparable [Additional file 2: Fig. S7j, k; *EndoA2*^*fl/fl*^ = (17.68 ± 1.71) μm vs. *EndoA2*^*fl/fl*^ × *Nefh-Cre* = (18.56 ± 1.33) μm]. These data suggest that EndoA2 is involved in regulating Piezo2-evoked mechanically sensitive currents in large DRG neurons.

### EndoA2 controls Piezo2 trafficking and mechanical sensitivity via the SH3 domain

**Fig. 6:**
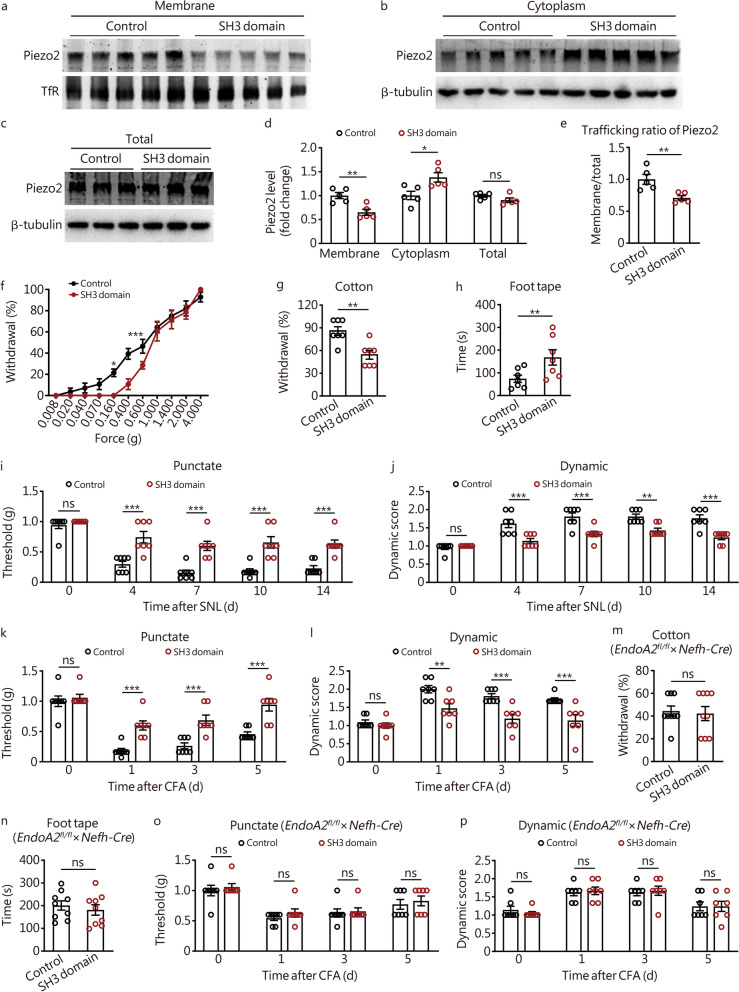
EndoA2 controls Piezo2 trafficking and mechanical sensitivity via the SH3 domain. **a–d** The effects of overexpressing the SH3 domain in NF200-positive (NF200^+^) DRG neurons on the expression of Piezo2 in the DRG membrane fraction, cytoplasmic fraction and total lysate. *n* = 5. **e** The effects of overexpressing the SH3 domain in NF200^+^ DRG neurons on the trafficking ratio of Piezo2 in DRGs.* n* = 5. The influence of overexpressing the SH3 domain in NF200^+^ DRG neurons on mechanical sensitivity was evaluated by various von Frey filament (**f**), cotton (**g**) and foot tape (**h**) tests. *n* = 7. **i–l** The effects of overexpressing the SH3 domain in NF200^+^ DRG neurons on the punctate and dynamic mechanical allodynia induced by SNL and CFA. *n* = 7. **m, n** Overexpression of the SH3 domain in NF200^+^ DRG neurons did not alter the touch sensitivity of *EndoA2*^*fl/fl*^ × *Nefh-Cre* mice. *n* = 9. **o, p** The effects of overexpressing the SH3 domain in NF200^+^ DRG neurons on CFA-induced mechanical allodynia in *EndoA2*^*fl/fl*^ × *Nefh-Cre* mice. *n* = 7. Two-tailed independent Student’s *t* test (**d**, **e**, **g**, **h**, **m**, **n**); Two-way ANOVA followed by Bonferroni’s multiple comparisons test (**f**, **i**–**l**, **o**, **p**). ^*^P < 0.05, ^**^P < 0.01, ^***^P < 0.001, ns non-significant. The error bars indicate the SEMs. TfR transferrin receptor, SNL spinal nerve ligation, CFA complete Freund’s adjuvant, EndoA2 endophilin A2

Since EndoA2 interacts with Piezo2 through the C-terminal SH3 domain (Fig. 4h, i), we further investigated the function of the EndoA2 C-terminal SH3 domain. We injected AAV encoding the SH3 domain into the DRG (L_4_ and L_5_) under the control of the neuronal promoter Syn and the DIO Cre-on system (rAAV-Syn-DIO-EndoA2-SH3-domain-Flag) in *Nefh-Cre* mice to overexpress the SH3 domain in NF200^+^ DRG neurons. Flag was observed in DRG NF200^+^ neurons of AAV solution-injected mice (Additional file 2: Fig. S8a), confirming transgene expression in DRG neurons. The data showed that overexpression of the SH3 domain significantly decreased the membrane and increased the cytoplasm but did not change the total expression of Piezo2 in DRG neurons (Fig. 6a–d). The trafficking ratio of Piezo2 was decreased in DRG neurons after overexpression of the SH3 domain (Fig. 6e). The behavioral data showed that overexpression of the SH3 domain significantly decreased the touch sensitivity of mice (Fig. 6f–h). The punctate and dynamic mechanical hypersensitivity induced by SNL (Fig. 6i, j) and CFA (Fig. 6k, l) were also inhibited after overexpression of the SH3 domain. Moreover, we detected the effects of overexpressing the SH3 domain on mechanical sensitivity in *EndoA2* conditional knockout mice (*EndoA2*^*fl/fl*^ × *Nefh-Cre* mice). The data revealed that overexpression of the SH3 domain did not alter the touch sensitivity of *EndoA2*^*fl/fl*^ × *Nefh-Cre* mice (Fig. 6m, n). The effects of overexpressing the SH3 domain on mechanical allodynia were also disabled in *EndoA2*^*fl/fl*^ × *Nefh-Cre* mice (Fig. 6o, p), indicating that the SH3 domain regulates mechanical sensitivity by competitively inhibiting the effect of EndoA2 on Piezo2. These data suggest that EndoA2 controls Piezo2 trafficking and mechanical sensitivity through the SH3 domain.

### KIF5B is involved in EndoA2-mediated Piezo2 membrane trafficking

**Fig. 7:**
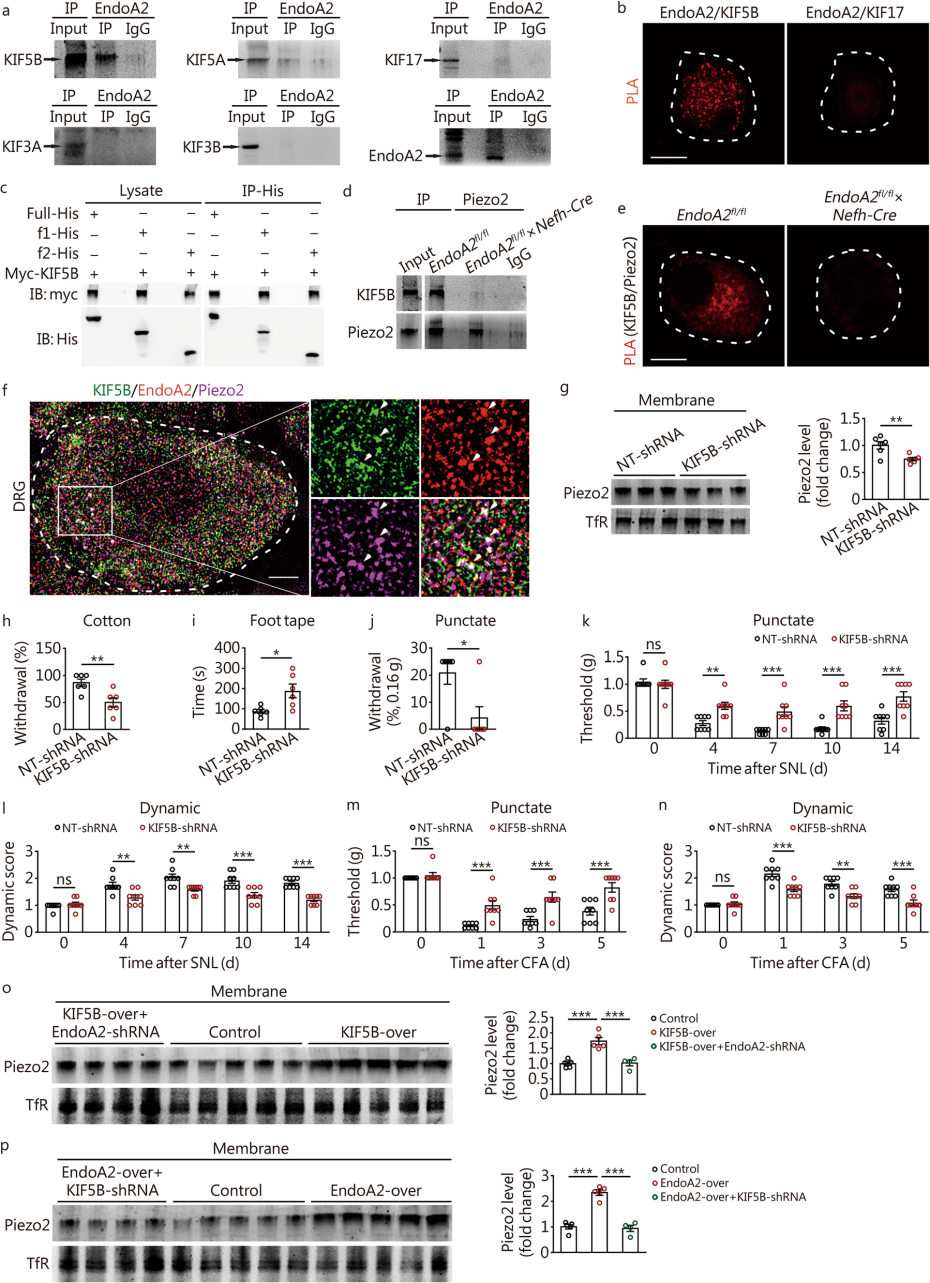
KIF5B is involved in EndoA2-mediated Piezo2 membrane trafficking. **a** Co-IP showing the EndoA2/KIF interaction in the DRG. DRG lysates were immunoprecipitated with an EndoA2 antibody and immunoblotted with KIF5B, KIF5A, KIF17, KIF3A, KIF3B and EndoA2 antibodies as indicated. This experiment was repeated 3 times. **b** Proximity ligation assay (PLA) shows positive signals of EndoA2/KIF5B interaction in cultured DRG neurons, and staining was absent with incubation of EndoA2 and KIF17 antibody (7 images from two repeats). Scale bar = 15 μm. **c** Interaction between KIF5B and EndoA2 mutants. The EndoA2 mutants were transiently coexpressed with KIF5B, and the cell lysates were immunoprecipitated with antibodies as indicated. This experiment was repeated 3 times. **d** The interaction level between Piezo2 and KIF5B was examined by Co-IP in *EndoA2*^*fl/fl*^ and *EndoA2*^*fl/fl*^ × *Nefh-Cre* mouse DRG lysates. DRG lysates were immunoprecipitated with Piezo2 antibody and immunoblotted with KIF5B and Piezo2 antibody as indicated. This experiment was repeated 3 times. **e** PLA shows positive signals of KIF5B/Piezo2 interaction in cultured DRG neurons of *EndoA2*^*fl/fl*^ mice, and staining was absent in cultured DRG neurons of *EndoA2*^*fl/fl*^ × *Nefh-Cre* mice (5 images from two repeats). Scale bar = 15 μm. **f** High-resolution images show the colocalization of KIF5B, EndoA2 and Piezo2 in DRG neurons. Arrows indicate colocalization. Scale bar = 5 μm. **g** Changes in the membrane expression of Piezo2 after *KIF5B* knockdown in the DRG. *n* = 6. The behaviors of NT-shRNA- and KIF5B-shRNA-injected mice were evaluated by cotton (**h**), foot tape (**i**) and 0.16 g punctate (**j**) tests. The same groups of mice were used for each pain behavioral test. The inter-test interval was at least 2 h. *n* = 6. **k–n** The effects of *KIF5B* knockdown on the punctate and dynamic mechanical allodynia induced by SNL and CFA. *n* = 8. **o** *EndoA2* knockdown inhibited the increased expression of Piezo2 in the membrane of DRGs induced by KIF5B overexpression. *n* = 4–5. **p** *KIF5B* knockdown repressed the increased expression of Piezo2 in the membrane of DRGs induced by EndoA2 overexpression. *n* = 4–5. Two-tailed independent Student’s *t* test (**g**–**j**); Two-way ANOVA followed by Bonferroni’s multiple comparisons test (**k**–**n**); One-way ANOVA followed by Tukey’s multiple comparisons test (**o**, **p**). ^*^P < 0.05, ^**^P < 0.01, ^***^P < 0.001, ns non-significant. The error bars indicate the SEMs. EndoA2 endophilin A2, DRG dorsal root ganglion, TfR transferrin receptor, NT nontargeting, SNL spinal nerve ligation, CFA complete Freund’s adjuvant

Then, we investigated the underlying mechanism of EndoA2-mediated membrane trafficking of Piezo2. Study has shown that motor protein kinesins play a crucial role in neuronal cargo trafficking [22]. To determine whether motor protein kinesins are involved in the trafficking of Piezo2 mediated by EndoA2, we first examined the interaction between EndoA2 and several motor proteins by the Co-IP method. The results showed that EndoA2 had a potential interaction with KIF5B and barely with KIF5A, KIF17, KIF3A or KIF3B in mouse DRG protein extracts (Fig. 7a). PLA analysis confirmed the close proximity of EndoA2 and KIF5B in DRG neurons (Fig. 7b). To define how EndoA2 interacts with KIF5B, we constructed EndoA2 deletion mutants, which failed to induce the function of the SH3 domain (f1) and SH3 and PRD domains (f2) (Fig. 4h). Co-IP data showed that EndoA2-full, -f1 and -f2 all potentially interacted with KIF5B (Fig. 7c). These results indicate that EndoA2 may interact with KIF5B through the N-terminal BAR domain. Next, we explored whether KIF5B participated in EndoA2-mediated Piezo2 membrane trafficking. Co-IP results showed that KIF5B potentially interacted with Piezo2 in the DRGs of *EndoA2*^*fl/fl*^ mice, whereas the interaction between KIF5B and Piezo2 was lost in *EndoA2*^*fl/fl*^ × *Nefh-Cre* mice (Fig. 7d). PLA analysis confirmed the close proximity of KIF5B and Piezo2 in DRG neurons of *EndoA2*^*fl/fl*^ mice but not in DRG neurons of *EndoA2*^*fl/fl*^ × *Nefh-Cre* mice (Fig. 7e). This suggests that EndoA2 may function as an adaptor protein that mediates the interaction between KIF5B and Piezo2 in DRG neurons. Considering the potential interaction among KIF5B, EndoA2 and Piezo2, we performed triple immunostaining, and high-resolution images were captured by SIM. The data showed that (19.33 ± 2.96)% of EndoA2 colocalized with Piezo2 and KIF5B, (23.33 ± 2.60)% of Piezo2 colocalized with EndoA2 and KIF5B, and (14.00 ± 2.08)% of KIF5B colocalized with EndoA2 and Piezo2 in DRG neurons (Fig. 7f, Additional file 2: Fig. S8b). KIF5B, EndoA2 and Piezo2 were also colocalized in the sciatic nerves of mice (Additional file 2: Fig. S8c). We further injected AAV encoding KIF5B-shRNA into the DRG of mice to knockdown *KIF5B* in DRG tissues (Additional file 2: Fig. S8d). The data showed that the surface accumulation of Piezo2 in mouse DRGs was decreased after injection of KIF5B-shRNA (Fig. 7g). Behavioral data showed that knockdown of *KIF5B* significantly decreased the touch sensitivity of mice (Fig. 7h–j). The punctate and dynamic mechanical allodynia induced by SNL and CFA were also inhibited by KIF5B-shRNA (Fig. 7k–n). Additionally, we injected AAV encoding KIF5B into the DRG to overexpress KIF5B in DRG tissues (Additional file 2: Fig. S8e) and found that overexpression of KIF5B markedly increased the membrane expression of Piezo2 in DRG neurons (Fig. 7o). Interestingly, EndoA2-shRNA suppressed the increase in Piezo2 membrane expression induced by KIF5B overexpression (Fig. 7o). Furthermore, we injected AAV encoding EndoA2 into the DRG to overexpress EndoA2 in DRGs (Additional file 2: Fig. S8f) and found that overexpression of EndoA2 significantly increased the membrane expression of Piezo2 in DRGs (Fig. 7p). Importantly, KIF5B-shRNA inhibited the increased expression of Pizeo2 on the cell surface induced by EndoA2 overexpression (Fig. 7p). These results suggest that the motor protein KIF5B plays a critical role in EndoA2-mediated Piezo2 membrane trafficking.

### EndoA2 interference impairs tactile sense and mechanical hypersensitivity in nonhuman primates

**Fig. 8:**
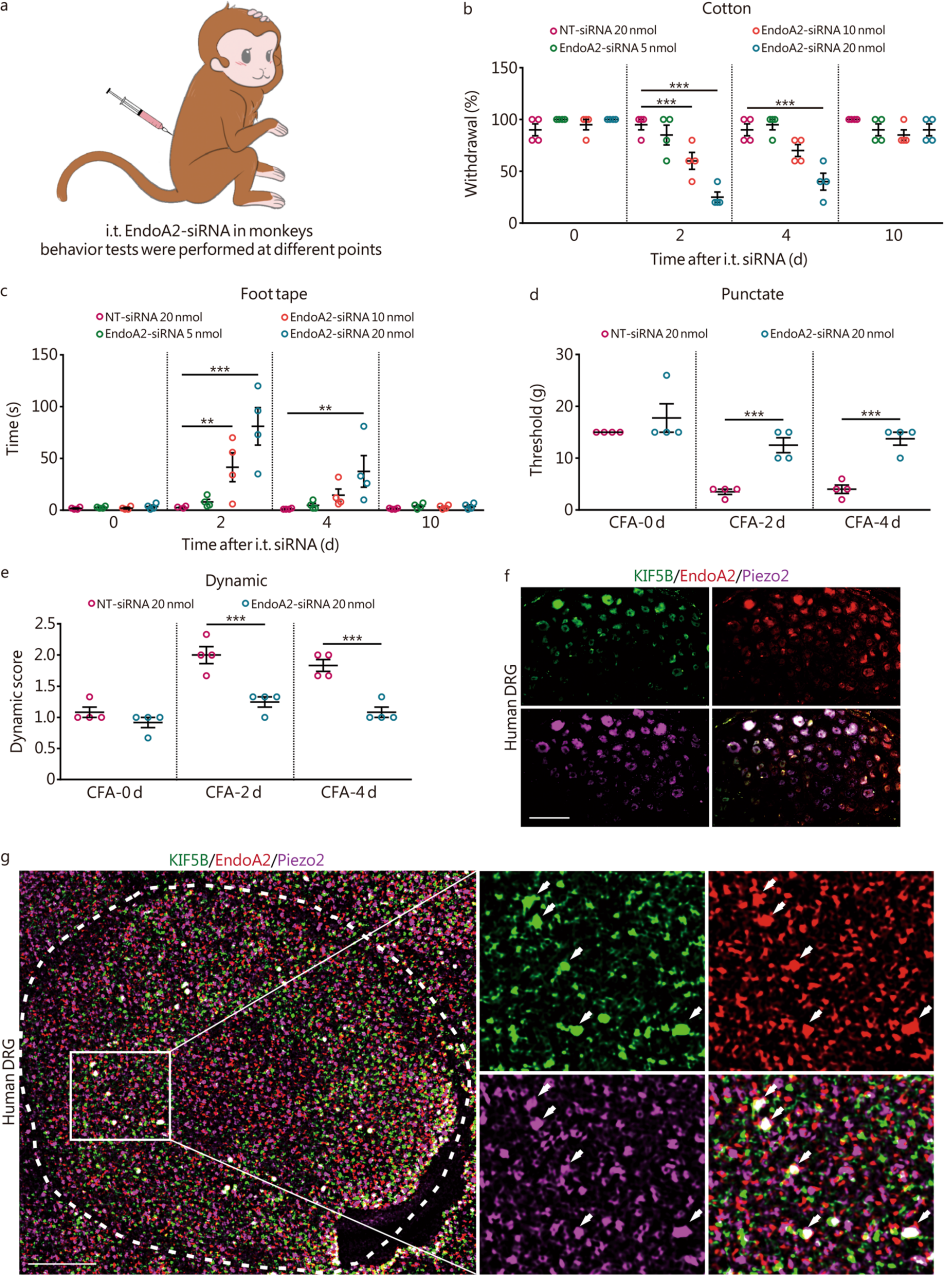
EndoA2-siRNA impairs tactile sense and mechanical hypersensitivity in nonhuman primates. **a** Monkeys were administered EndoA2-siRNA via intrathecal injection (i.t.), and then the behaviors were tested at different time points. Touch sensitivity changes in monkeys after i.t. of EndoA2-siRNA (5, 10, and 20 nmol) in the cotton (**b**) and foot tape (**c**) tests. *n* = 4. The effects of EndoA2-siRNA (20 nmol) on the punctate mechanical hypersensitivity (**d**) and dynamic mechanical hypersensitivity (**e**) induced by CFA in monkeys. *n* = 4. **f** Colocalization of KIF5B, EndoA2 and Piezo2 in human thoracic DRG sections. Scale bar = 200 μm. **g** High-resolution images showing the colocalization of KIF5B, EndoA2 and Piezo2 in human thoracic DRG neurons. Arrows indicate colocalization. Scale bar = 10 μm. Two-way ANOVA followed by Bonferroni’s multiple comparisons test (**b**–**e**). ^**^P < 0.01, ^***^P < 0.001. The error bars indicate the SEMs. EndoA2 endophilin A2, NT nontargeting, CFA complete Freund’s adjuvant, DRG dorsal root ganglion

Nonhuman primates have been tested for pain research because of their phylogenetic proximity to humans [44]. We tested whether i.t. of EndoA2-siRNA suppressed touch sensitivity and mechanical hypersensitivity in nonhuman primates (Fig. 8a). The knockdown effect of EndoA2-siRNA was verified in monkey Vero cells (Additional file 2: Fig. S9a). The behavioral data showed that EndoA2-siRNA dose-dependently decreased monkey touch sensitivity induced by cotton (Fig. 8b) and foot tape (Fig. 8c, Additional file 3: Movie S1). The reduced tactile response began in 2 d and returned to normal 10 d after injection of EndoA2-siRNA (Fig. 8b, c), indicating that siRNA was metabolically cleared on Day 10 in vivo and suggesting that EndoA2-siRNA does work in vivo. EndoA2-siRNA-injected monkeys had a normal withdrawal threshold to von Frey filament-evoked punctate mechanical stimulation and brush-evoked dynamic mechanical stimulation (Additional file 2: Fig. S9b, c). The sensitivity of monkeys to heat stimulation was not altered by EndoA2-siRNA in the tail-flick test (Additional file 2: Fig. S9d). Notably, EndoA2-siRNA significantly relieved punctate mechanical hypersensitivity and dynamic mechanical hypersensitivity induced by CFA in nonhuman primates (Fig. 8d, e). Thus, this suggests that EndoA2 participates in touch and mechanical allodynia perception in nonhuman primates.

### KIF5B, EndoA2 and Piezo2 are coexpressed in human sensory neurons

To evaluate the translational potential of this study, we further tested the distribution pattern of EndoA2 in human DRG neurons. Immunostaining showed that EndoA2 was colocalized with NF200^+^ neurons in human DRG sections (Additional file 2: Fig. S10a). Size frequency analysis showed overlapping distribution patterns of EndoA2^+^ and NF200^+^ neurons in human DRG sections (Additional file 2: Fig. S10b). EndoA2 was also expressed in NF200^+^ A fibers in the human sciatic nerves (Additional file 2: Fig. S10c). Considering the potential interaction among KIF5B, EndoA2 and Piezo2 in DRG neurons of mice, we performed triple immunostaining of KIF5B, EndoA2 and Piezo2 in human DRG and sciatic nerve sections. The data showed that KIF5B, EndoA2 and Piezo2 were colocalized in human DRG neurons (Fig. 8f) and sciatic nerves (Additional file 2: Fig. S10d). High-resolution images confirmed the colocalization of KIF5B, EndoA2 and Piezo2 in human DRG neurons (Fig. 8g) and indicated that the three proteins were very close together. Colocalization analysis showed that (11.67 ± 2.03)% of EndoA2 colocalized with Piezo2 and KIF5B, (10.00 ± 2.31)% of Piezo2 colocalized with EndoA2 and KIF5B, and (14.00 ± 2.65)% of KIF5B colocalized with EndoA2 and Piezo2 in human DRG neurons (Fig. 8g, Additional file 2: Fig. S10e). Therefore, KIF5B, EndoA2 and Piezo2 are co-expressed in human sensory neurons.

## Discussion

**Fig. 9:**
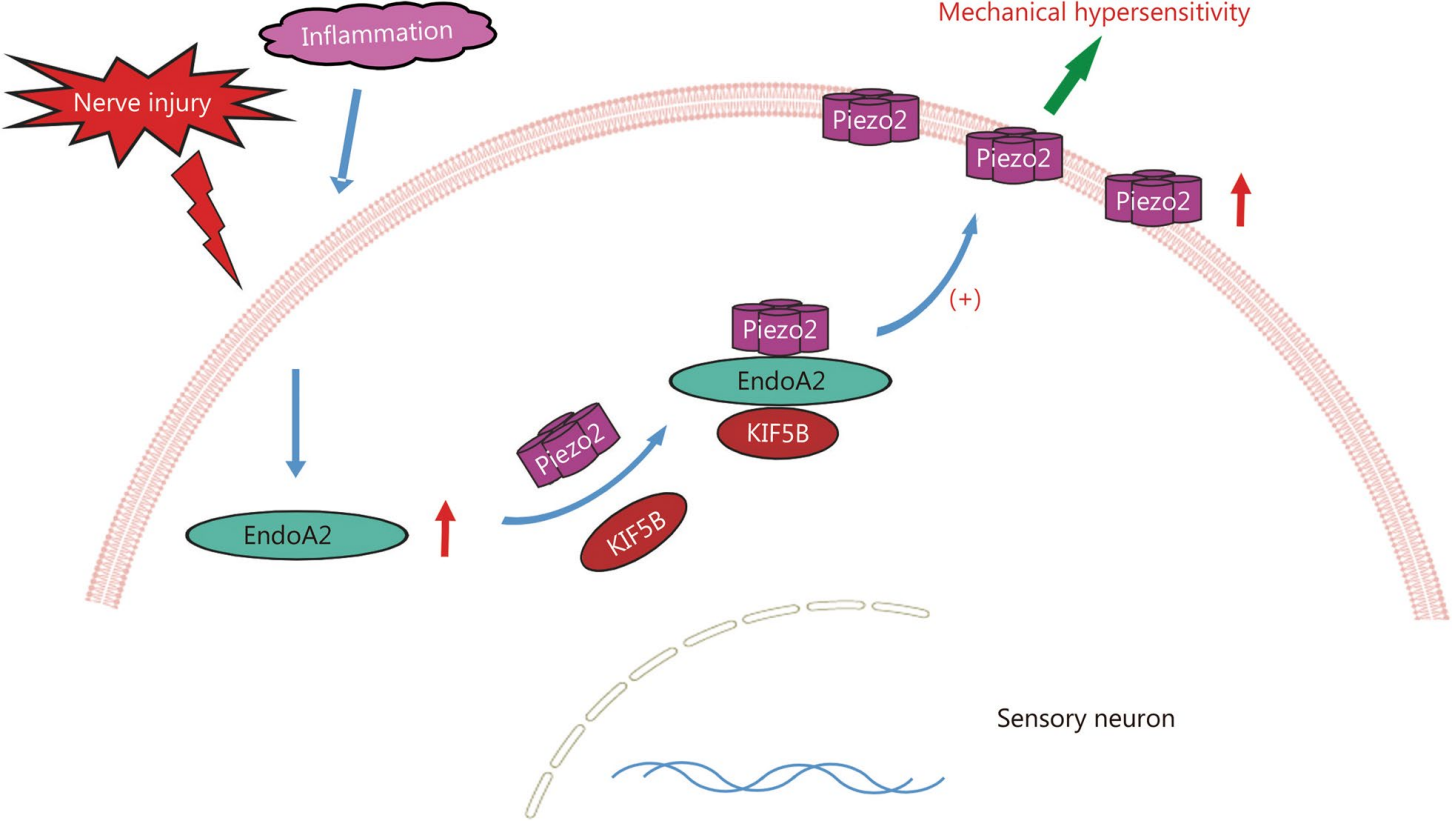
Hypothetical model illustrating that endophilin A2 (EndoA2) interacts with Piezo2 and KIF5B to form a complex to regulate the membrane trafficking of Piezo2 in sensory neurons and thus contributes to mechanical hypersensitivity

In the present study, we revealed that EndoA2 is distributed in NF200^+^ medium-to-large diameter sensory neurons and controls touch, punctate mechanical allodynia and dynamic mechanical allodynia in mice and nonhuman primates. Moreover, EndoA2 interacts with the mechanically sensitive ion channel Piezo2 through the C-terminal SH3 domain and combines with the motor protein KIF5B through the N-terminal BAR domain, promoting the membrane trafficking of Pizeo2 in sensory neurons under the forward transport of KIF5B and thus contributing to mechanical hypersensitivity (Fig. 9).

EndoA2 is an evolutionarily conserved protein that is widely expressed in neural and nonneural tissues and is involved in the regulation of various physiological and pathological processes [12, 13]. In the cardiovascular system, EndoA2 decreases the expression of the calcium-activated chloride channel TMEM16A by regulating its ubiquitination and autophagy and participates in hypertension-induced vascular remodeling [15]. In the central nervous system, EndoA2 is involved in regulating synaptic transmission, which may be related to nerve defects, including epilepsy and neurodegeneration [17, 18]. However, the expression pattern of EndoA2 in the peripheral sensory nervous system and its role in sensory regulation remain unclear. Our data show that EndoA2 is highly expressed in the peripheral nervous system and is mainly distributed in NF200^+^ medium-to-large DRG neurons in mice and humans. Losing the expression of EndoA2 in sensory neurons significantly impairs tactile sensation, punctate mechanical allodynia and dynamic mechanical allodynia in mice and nonhuman primates. This suggests that EndoA2 may be a potential molecular target for the clinical treatment of mechanical allodynia. The development of small molecule compounds for EndoA2 is an important direction of future research. Previous scRNA-seq datasets showed that *EndoA2* mRNA is distributed in most DRG neurons, including small and medium-to-large diameter neurons (https://kleintools.hms.harvard.edu/tools/springViewer_1_6_dev.html?datasets/Sharma2019/all, and http://mousebrain.org/adolescent/genes.html). Our data show that EndoA2 protein is mainly expressed in medium-to-large diameter neurons, with a small amount expressed in small-diameter neurons. This may suggest that there is a difference in the expression pattern between the protein and mRNA of EndoA2. The underlying mechanism of this difference is unclear and requires further study.

The mechanosensitive ion channel Piezo2 is widely distributed in human and mouse sensory neurons, which sense mechanical force signals and convert mechanical force signals into electrical signals to detect touch and mechanical pain [19, 20]. Piezo2 is a nonselective cation channel that can mediate the influx of calcium ions, and mutation of Piezo2 in DRG neurons can significantly reduce tactile sensitivity and mechanical allodynia in mice and humans [19, 20, 37, 60]. In addition to sensing touch and mechanical pain, Piezo2 is also involved in regulating proprioception, airway stretch, ultrasonic hearing and mechanical itch [54, 61–63]. The trafficking of ion channels from the cytoplasm to the cell membrane is an important basis for their physiological functions. Our data show that EndoA2 interacts with Piezo2 through the C-terminal SH3 domain in sensory neurons. Loss of EndoA2 significantly reduced the surface area and increased the cytoplasm but did not change the total expression of Piezo2 in DRG neurons. Deletion of EndoA2 also decreases Piezo2-mediated MA currents. This indicates that EndoA2 is the key regulator of Piezo2 trafficking from the cytoplasm to the plasma membrane in sensory neurons. It is traditionally believed that EndoA2 is mainly involved in the regulation of endocytosis and exocytosis [64]. However, our findings show that EndoA2 also regulates the membrane trafficking of ion channels in the somatosensory nervous system, suggesting a novel function of EndoA2, but more research is needed to support this conclusion. We know that changes in ion channel current depend on two factors: the membrane expression abundance of the channel and the characteristics of the individual channel. Our data show that loss of EndoA2 significantly reduces the membrane abundance of Piezo2 and decreases Piezo2-mediated MA currents. These results indicate that the effect of EndoA2 on Piezo2 membrane expression may be one of the mechanisms by which EndoA2 regulates Piezo2-mediated MA currents. However, whether EndoA2 also regulates the Piezo2 current by altering the characteristics of individual Piezo2 channels is unclear. Previous work shows that constitutive deletion of Piezo2 led to perinatal lethality [19], which suggests that Piezo2’s function is crucial, so blocking Piezo2 directly in humans could have serious side effects. Our data reveal that EndoA2 interacts with Piezo2 through the C-terminal SH3 domain and that overexpression of the C-terminal SH3 domain in sensory neurons significantly alleviates mechanical allodynia in mice. The next steps are to find the key peptide in the SH3 domain that regulates Piezo2 and to conduct clinical studies to further develop small-molecule peptide drugs with few side effects for the treatment of mechanical pain.

The trafficking of ion channels toward the cell membrane is an essential step in regulating neuronal functions, such as the response to mechanical or thermal stimulation [23, 24, 65]. Study shows that motor protein kinesins play crucial roles in the trafficking of neuronal cargos such as ion channels and receptors [22]. Our previous research has shown that KIF3A is involved in the regulation of sodium channel Nav1.6 membrane trafficking in DRG neurons, which contributes to the pathological pain induced by nerve injury or the chemotherapeutic drug oxaliplatin [23]. Kinesin KIF17 mediates the membrane trafficking of TRPM3, a thermosensitive ion channel in DRG neurons, and then participates in the regulation of heat hyperalgesia evoked by chronic inflammation and nerve injury [24]. Peer research demonstrates that the kinesin protein KIF13B contributes to the membrane trafficking of TRPV1 in DRG neurons [66]. Subcellular fractionation from the striatum shows that deletion of KIF5B reduces the amount of dopamine D2 receptor in synaptic plasma membranes [26]. Suppressing the function of KIF5B significantly reduces the axonal targeting and forward trafficking of Kv3.1 channels [27]. Here, we found that EndoA2 is an adaptor protein that mediates the interaction between KIF5B and Piezo2 in DRG neurons. EndoA2, KIF5B and Piezo2 are coexpressed in DRG neurons of mice and humans. Knockdown of *KIF5B* decreases the membrane trafficking of Piezo2 and reduces the tactile sensitivity and mechanical allodynia of mice. Interestingly, the increase in Piezo2 membrane expression induced by EndoA2 overexpression was reversed by KIF5B shRNA in DRG neurons. These results suggest that the motor protein KIF5B plays a critical role in EndoA2-mediated Piezo2 membrane trafficking in sensory neurons. The motor protein kinesin KIF5B converts the chemical energy stored in ATP into mechanical kinetic energy that powers movement along microtubules and protein Piezo2 trafficking. However, the underlying mechanism by which KIF5B converts chemical energy into mechanical energy is unknown and requires further investigation.

## Conclusions

In summary, we reveal a previously unknown role of EndoA2 in mechanical sensitivity. EndoA2 is primarily expressed in NF200^+^ medium-to-large diameter sensory neurons and controls touch and mechanical allodynia in mice and nonhuman primates. Furthermore, EndoA2 interacts with Piezo2 and promotes the membrane trafficking of Pizeo2 in sensory neurons. Additionally, as an adaptor protein, EndoA2 also binds to KIF5B, and KIF5B is involved in the EndoA2-mediated membrane trafficking process of Piezo2. Ultimately, our work reveals a novel cellular mechanism essential for mechanical sensitivity and identifies a potential new strategy for the treatment of mechanical allodynia.

### Supplementary Information

**Additional file 1: Table S1** The complete list of genotypes, treatment groups, animal sex, and total animal numbers for each figure. **Table S2** The specific primer sequences of endophilins.

**Additional file 2: Fig. S1** SNL increases the expression of EndoA2 in sensory neurons. **Fig. S2** Deletion of EndoA2 in DRG neurons did not change the heat hyperalgesia induced by SNL and CFA. **Fig. S3** Loss of EndoA2 in DRG neurons suppresses punctate- and brush-evoked CPA in mice with SNL, CFA and VCR treatment. **Fig. S4** *EndoA2*^*fl/fl*^ × *Nefh-Cre* mice display normal sensory neurons and their central innervations. **Fig. S5** Loss or rescue of EndoA2 in NF200-positive (NF200^+^) DRG neurons did not alter heat hyperalgesia induced by SNL and CFA. **Fig. S6** Deletion of EndoA2 in TRPV1-positive small-diameter DRG neurons did not change the touch and pain behaviors. **Fig. S7** Loss of EndoA2 decreases the membrane trafficking of Piezo2 in sciatic nerves. **Fig. S8** KIF5B, EndoA2 and Piezo2 are coexpressed in the sciatic nerves of mice. **Fig. S9** EndoA2-siRNA does not change the punctate, dynamic and heat threshold of nonhuman primates. **Fig. S10** The distribution of EndoA2 in the DRG of humans.

**Additional file 3: Movie S1** Foot tape behavior in nonhuman primates.

## Data Availability

All data generated or analyzed during this study are included in this published article.

## Abbreviations

AAV: Adeno-associated virus
ASIC2: Acid sensing ion channel 2
ASIC3: Acid sensing ion channel 3
BAR: Bin-amphiphysin-Rvs
CFA: Complete Freund’s adjuvant
CGRP: Calcitonin gene-related peptide
Co-IP: Co-immunoprecipitation
CPA: Conditional place aversion
DRG: Dorsal root ganglion
EndoA2: Endophilin A2
IA: Intermediately adapting
IB4: Isolectin B4
i.p.: Intraperitoneal injection
i.t.: Intrathecal injection
KIF: Kinesin superfamily protein
KIF5B: Kinesin family member 5B
LTMRs: Low-threshold mechanoreceptors
MA: Mechanically activated
NF200: Neurofilament-200
NMDAR: N-methyl-D-aspartate receptor
P1KO: Piezo1 knockout
PFA: Paraformaldehyde
PLA: Proximity ligation assay
PRD: Proline-rich domain
qPCR: Quantitative real-time polymerase chain reaction
RA: Rapidly adapting
RT: Room temperature
SA: Slowly adapting
SH3: Src homology 3
SIM: Structured illumination microscopy
SNL: Spinal nerve ligation
TAM: Tamoxifen
TRPM3: Transient receptor potential M3 cation channels
VCR: Vincristine
VEGFR: Vascular endothelial growth factor receptor
