# Supplementary material for: Endophilin A2 controls touch and mechanical allodynia via kinesin-mediated Piezo2 trafficking
Source: Mil Med Res. 2024 Mar 12;11:17. doi: 10.1186/s40779-024-00520-z (PMC10929226; doi:10.1186/s40779-024-00520-z)
Supplement: Supplementary file 1 — Additional file 1: Table S1 The complete list of genotypes, treatment groups, animal sex, and total animal numbers for each figure. Table S2 The specific primer sequences of endophilins. [file 40779_2024_520_MOESM1_ESM.pdf]

Table S1 The complete list of genotypes, treatment groups, animal sex, and total animal numbers for each figure

Type of experiment	Group	Total animals
Behavioral testing		
Fig. 2b	*EndoA2*$^{fl/fl}$ + TAM (*n* = 12; 6M/6F)<br>*EndoA2*$^{fl/fl}$ × *AvCreERT2* + TAM (*n* = 12; 6M/6F)	*n* = 24 mice
Fig. 2c	*EndoA2*$^{fl/fl}$ + TAM (*n* = 12; 6M/6F)<br>*EndoA2*$^{fl/fl}$ × *AvCreERT2* + TAM (*n* = 12; 6M/6F)	Same mice as in Fig. 2b
Fig. 2d	*EndoA2*$^{fl/fl}$ + TAM (*n* = 12; 6M/6F)<br>*EndoA2*$^{fl/fl}$ × *AvCreERT2* + TAM (*n* = 12; 6M/6F)	Same mice as in Fig. 2b
Fig. 2e	*EndoA2*$^{fl/fl}$ + TAM (*n* = 12; 6M/6F)<br>*EndoA2*$^{fl/fl}$ × *AvCreERT2* + TAM (*n* = 12; 6M/6F)	Same mice as in Fig. 2b
Fig. 2g	*EndoA2*$^{fl/fl}$ + TAM (*n* = 8; 4M/4F)<br>*EndoA2*$^{fl/fl}$ × *AvCreERT2* + TAM (*n* = 8; 4M/4F)	Same mice as in Fig. 2b
Fig. 2h	*EndoA2*$^{fl/fl}$ + TAM (*n* = 8; 4M/4F)<br>*EndoA2*$^{fl/fl}$ × *AvCreERT2* + TAM (*n* = 8; 4M/4F)	Same mice as in Fig. 2b
Fig. 2j	*EndoA2*$^{fl/fl}$ + TAM (*n* = 9; 5M/4F)<br>*EndoA2*$^{fl/fl}$ × *AvCreERT2* + TAM (*n* = 9; 5M/4F)	*n* = 18 mice
Fig. 2k	*EndoA2*$^{fl/fl}$ + TAM (*n* = 9; 5M/4F)<br>*EndoA2*$^{fl/fl}$ × *AvCreERT2* + TAM (*n* = 9; 5M/4F)	Same mice as in Fig. 2j
Fig. 2m	*EndoA2*$^{fl/fl}$ + TAM (*n* = 8; 4M/4F)<br>*EndoA2*$^{fl/fl}$ × *AvCreERT2* + TAM (*n* = 8; 4M/4F)	*n* = 16 mice
Fig. 2n	*EndoA2*$^{fl/fl}$ + TAM (*n* = 8; 4M/4F)<br>*EndoA2*$^{fl/fl}$ × *AvCreERT2* + TAM (*n* = 8; 4M/4F)	Same mice as in Fig. 2m
Fig. 2p	*EndoA2*$^{fl/fl}$ + TAM (*n* = 6; 3M/3F)<br>*EndoA2*$^{fl/fl}$ × *AvCreERT2* + TAM (*n* = 6; 3M/3F)	*n* = 12 mice

Fig. 2q	*EndoA2*$^{fl/fl}$ + TAM (*n* = 6; 4M/2F)<br>*EndoA2*$^{fl/fl}$ × *AvCreERT2* + TAM (*n* = 6; 4M/2F)	*n* = 12 mice
Fig. 2r	*EndoA2*$^{fl/fl}$ + TAM (*n* = 6; 3M/3F)<br>*EndoA2*$^{fl/fl}$ × *AvCreERT2* + TAM (*n* = 6; 3M/3F)	*n* = 12 mice
Fig. 2s	*EndoA2*$^{fl/fl}$ + TAM (*n* = 6; 2M/4F)<br>*EndoA2*$^{fl/fl}$ × *AvCreERT2* + TAM (*n* = 6; 3M/3F)	*n* = 12 mice
Fig. 3b	*EndoA2*$^{fl/fl}$ (*n* = 6; 5M/1F)<br>*EndoA2*$^{fl/fl}$ × *Nefh-Cre* (*n* = 6; 3M/3F)<br>Rescue (*n* = 6; 3M/3F)	*n* = 18 mice
Fig. 3c	*EndoA2*$^{fl/fl}$ (*n* = 6; 5M/1F)<br>*EndoA2*$^{fl/fl}$ × *Nefh-Cre* (*n* = 6; 3M/3F)<br>Rescue (*n* = 6; 3M/3F)	Same mice as in Fig. 3b
Fig. 3d	*EndoA2*$^{fl/fl}$ (*n* = 6; 5M/1F)<br>*EndoA2*$^{fl/fl}$ × *Nefh-Cre* (*n* = 6; 3M/3F)<br>Rescue (*n* = 6; 3M/3F)	Same mice as in Fig. 3b
Fig. 3e	*EndoA2*$^{fl/fl}$ (*n* = 6; 5M/1F)<br>*EndoA2*$^{fl/fl}$ × *Nefh-Cre* (*n* = 6; 3M/3F)<br>Rescue (*n* = 6; 3M/3F)	Same mice as in Fig. 3b
Fig. 3f	*EndoA2*$^{fl/fl}$ (*n* = 6; 5M/1F)<br>*EndoA2*$^{fl/fl}$ × *Nefh-Cre* (*n* = 6; 3M/3F)<br>Rescue (*n* = 6; 3M/3F)	Same mice as in Fig. 3b
Fig. 3g	*EndoA2*$^{fl/fl}$ (*n* = 6; 6M)<br>*EndoA2*$^{fl/fl}$ × *Nefh-Cre* (*n* = 6; 6M)<br>Rescue (*n* = 6; 6M)	*n* = 18 mice
Fig. 3h	*EndoA2*$^{fl/fl}$ (*n* = 6; 6M)<br>*EndoA2*$^{fl/fl}$ × *Nefh-Cre* (*n* = 6; 6M)<br>Rescue (*n* = 6; 6M)	Same mice as in Fig. 3g
Fig. 3i	*EndoA2*$^{fl/fl}$ (*n* = 6; 6F)<br>*EndoA2*$^{fl/fl}$ × *Nefh-Cre* (*n* = 6; 6F)<br>Rescue (*n* = 6; 3M/3F)	*n* = 18 mice
Fig. 3j	*EndoA2*$^{fl/fl}$ (*n* = 6; 6F)<br>*EndoA2*$^{fl/fl}$ × *Nefh-Cre* (*n* = 6; 6F)<br>Rescue (*n* = 6; 3M/3F)	Same mice as in Fig. 3i
Fig. 6f	Control (*n* = 7; 2M/5F)<br>SH3 domain (*n* = 7; 3M/4F)	*n* = 14 mice

Fig. 6g	Control (*n* = 7; 2M/5F)<br>SH3 domain (*n* = 7; 3M/4F)	Same mice as in Fig. 6f
Fig. 6h	Control (*n* = 7; 2M/5F)<br>SH3 domain (*n* = 7; 3M/4F)	Same mice as in Fig. 6f
Fig. 6i	Control (*n* = 7; 2M/5F)<br>SH3 domain (*n* = 7; 3M/4F)	Same mice as in Fig. 6f
Fig. 6j	Control (*n* = 7; 2M/5F)<br>SH3 domain (*n* = 7; 3M/4F)	Same mice as in Fig. 6f
Fig. 6k	Control (*n* = 7; 4M/3F)<br>SH3 domain (*n* = 7; 3M/4F)	*n* = 14 mice
Fig. 6l	Control (*n* = 7; 4M/3F)<br>SH3 domain (*n* = 7; 3M/4F)	Same mice as in Fig. 6k
Fig. 6m	Control (*n* = 9; 5M/4F)<br>SH3 domain (*n* = 9; 2M/7F)	*n* = 18 mice
Fig. 6n	Control (*n* = 9; 5M/4F)<br>SH3 domain (*n* = 9; 2M/7F)	Same mice as in Fig. 6m
Fig. 6o	Control (*n* = 7; 4M/3F)<br>SH3 domain (*n* = 7; 2M/5F)	Same mice as in Fig. 6m
Fig. 6p	Control (*n* = 7; 4M/3F)<br>SH3 domain (*n* = 7; 2M/5F)	Same mice as in Fig. 6m
Fig. 7h	NT-shRNA (*n* = 6; 6M)<br>KIF5B-shRNA (*n* = 6; 6M)	*n* = 12 mice
Fig. 7i	NT-shRNA (*n* = 6; 6M)<br>KIF5B-shRNA (*n* = 6; 6M)	Same mice as in Fig. 7h
Fig. 7j	NT-shRNA (*n* = 6; 6M)<br>KIF5B-shRNA (*n* = 6; 6M)	Same mice as in Fig. 7h

Fig. 7k	NT-shRNA (*n* = 8; 8M)<br>KIF5B-shRNA (*n* = 8; 8M)	*n* = 16 mice
Fig. 7l	NT-shRNA (*n* = 8; 8M)<br>KIF5B-shRNA (*n* = 8; 8M)	Same mice as in Fig. 7k
Fig. 7m	NT-shRNA (*n* = 8; 8M)<br>KIF5B-shRNA (*n* = 8; 8M)	*n* = 16 mice
Fig. 7n	NT-shRNA (*n* = 8; 8M)<br>KIF5B-shRNA (*n* = 8; 8M)	Same mice as in Fig. 7m
Fig. 8b	NT-siRNA (*n* = 4; 4M)<br>EndoA2-siRNA 5 nmol (*n* = 4; 4M)<br>EndoA2-siRNA 10 nmol (*n* = 4; 4M)<br>EndoA2-siRNA 20 nmol (*n* = 4; 4M)	*n* = 4 NHPs;<br>> 1 week washout between doses
Fig. 8c	NT-siRNA (*n* = 4; 4M)<br>EndoA2-siRNA 5 nmol (*n* = 4; 4M)<br>EndoA2-siRNA 10 nmol (*n* = 4; 4M)<br>EndoA2-siRNA 20 nmol (*n* = 4; 4M)	Same NHPs as in Fig. 8b;<br>> 1 week washout between doses
Fig. 8d	NT-siRNA (*n* = 4; 4M; left foot CFA)<br>EndoA2-siRNA 20 nmol (*n* = 4; 4M; right foot CFA)	Same NHPs as in Fig. 8b;<br>> 1 week washout between doses
Fig. 8e	NT-siRNA (*n* = 4; 4M; left foot CFA)<br>EndoA2-siRNA 20 nmol (*n* = 4; 4M; right foot CFA)	Same NHPs as in Fig. 8b;<br>> 1 week washout between doses
Fig. S2c	*EndoA2*$^{fl/fl}$ + TAM (*n* = 12; 6M/6F)<br>*EndoA2*$^{fl/fl}$ × *AvCreERT2* + TAM (*n* = 12; 6M/6F)	Same mice as in Fig. 2b
Fig. S2d	*EndoA2*$^{fl/fl}$ + TAM (*n* = 12; 6M/6F)<br>*EndoA2*$^{fl/fl}$ × *AvCreERT2* + TAM (*n* = 12; 6M/6F)	Same mice as in Fig. 2b
Fig. S2e	*EndoA2*$^{fl/fl}$ + TAM (*n* = 6; 3M/3F)<br>*EndoA2*$^{fl/fl}$ × *AvCreERT2* + TAM (*n* = 6; 3M/3F)	Same mice as in Fig. 2b

Fig. S2f	*EndoA2*$^{fl/fl}$ + TAM (*n* = 12; 6M/6F)<br>*EndoA2*$^{fl/fl}$ × *AvCreERT2* + TAM (*n* = 12; 6M/6F)	Same mice as in Fig. 2b
Fig. S2g	*EndoA2*$^{fl/fl}$ + TAM (*n* = 12; 6M/6F)<br>*EndoA2*$^{fl/fl}$ × *AvCreERT2* + TAM (*n* = 12; 6M/6F)	Same mice as in Fig. 2b
Fig. S2h	*EndoA2*$^{fl/fl}$ + TAM (*n* = 12; 6M/6F)<br>*EndoA2*$^{fl/fl}$ × *AvCreERT2* + TAM (*n* = 12; 6M/6F)	Same mice as in Fig. 2b
Fig. S2i	*EndoA2*$^{fl/fl}$ + TAM (*n* = 12; 6M/6F)<br>*EndoA2*$^{fl/fl}$ × *AvCreERT2* + TAM (*n* = 12; 6M/6F)	Same mice as in Fig. 2b
Fig. S2j	*EndoA2*$^{fl/fl}$ + TAM (*n* = 12; 6M/6F)<br>*EndoA2*$^{fl/fl}$ × *AvCreERT2* + TAM (*n* = 12; 6M/6F)	Same mice as in Fig. 2b
Fig. S2k	*EndoA2*$^{fl/fl}$ + TAM (*n* = 12; 6M/6F)<br>*EndoA2*$^{fl/fl}$ × *AvCreERT2* + TAM (*n* = 12; 6M/6F)	Same mice as in Fig. 2b
Fig. S2l	*EndoA2*$^{fl/fl}$ + TAM (*n* = 6; 3M/3F)<br>*EndoA2*$^{fl/fl}$ × *AvCreERT2* + TAM (*n* = 6; 4M/2F)	Same mice as in Fig. 2b
Fig. S2m	*EndoA2*$^{fl/fl}$ + TAM (*n* = 6; 3M/3F)<br>*EndoA2*$^{fl/fl}$ × *AvCreERT2* + TAM (*n* = 6; 3M/3F)	Same mice as in Fig. 2j
Fig. S3a	*EndoA2*$^{fl/fl}$ + TAM, pre (*n* = 6; 3M/3F)<br>*EndoA2*$^{fl/fl}$ + TAM, post (*n* = 6; 3M/3F)<br>*EndoA2*$^{fl/fl}$ × *AvCreERT2* + TAM, pre (*n* = 6; 3M/3F)<br>*EndoA2*$^{fl/fl}$ × *AvCreERT2* + TAM, post (*n* = 6; 3M/3F)	Same mice as in Fig. 2p
Fig. S3b	*EndoA2*$^{fl/fl}$ + TAM, pre (*n* = 6; 4M/2F)<br>*EndoA2*$^{fl/fl}$ + TAM, post (*n* = 6; 4M/2F)<br>*EndoA2*$^{fl/fl}$ × *AvCreERT2* + TAM, pre (*n* = 6; 4M/2F)<br>*EndoA2*$^{fl/fl}$ × *AvCreERT2* + TAM, post (*n* = 6; 4M/2F)	Same mice as in Fig. 2q
Fig. S3c	*EndoA2*$^{fl/fl}$ + TAM, pre (*n* = 6; 2M/4F)<br>*EndoA2*$^{fl/fl}$ + TAM, post (*n* = 6; 2M/4F)<br>*EndoA2*$^{fl/fl}$ × *AvCreERT2* + TAM, pre (*n* = 6; 3M/3F)<br>*EndoA2*$^{fl/fl}$ × *AvCreERT2* + TAM, post (*n* = 6; 3M/3F)	*n* = 12 mice
Fig. S3d	*EndoA2*$^{fl/fl}$ + TAM, pre (*n* = 6; 2M/4F)<br>*EndoA2*$^{fl/fl}$ + TAM, post (*n* = 6; 2M/4F)<br>*EndoA2*$^{fl/fl}$ × *AvCreERT2* + TAM, pre (*n* = 6; 3M/3F)<br>*EndoA2*$^{fl/fl}$ × *AvCreERT2* + TAM, post (*n* = 6; 3M/3F)	Same mice as in Fig. S3c

Fig. S3e	*EndoA2*$^{fl/fl}$ + TAM, pre (*n* = 6; 3M/3F)<br>*EndoA2*$^{fl/fl}$ + TAM, post (*n* = 6; 3M/3F)<br>*EndoA2*$^{fl/fl}$ × *AvCreERT2* + TAM, pre (*n* = 6; 3M/3F)<br>*EndoA2*$^{fl/fl}$ × *AvCreERT2* + TAM, post (*n* = 6; 3M/3F)	Same mice as in Fig. 2r
Fig. S3f	*EndoA2*$^{fl/fl}$ + TAM, pre (*n* = 6; 2M/4F)<br>*EndoA2*$^{fl/fl}$ + TAM, post (*n* = 6; 2M/4F)<br>*EndoA2*$^{fl/fl}$ × *AvCreERT2* + TAM, pre (*n* = 6; 3M/3F)<br>*EndoA2*$^{fl/fl}$ × *AvCreERT2* + TAM, post (*n* = 6; 3M/3F)	Same mice as in Fig. 2s
Fig. S3g	*EndoA2*$^{fl/fl}$ + TAM, pre (*n* = 6; 3M/3F)<br>*EndoA2*$^{fl/fl}$ + TAM, post (*n* = 6; 3M/3F)<br>*EndoA2*$^{fl/fl}$ × *AvCreERT2* + TAM, pre (*n* = 6; 2M/4F)<br>*EndoA2*$^{fl/fl}$ × *AvCreERT2* + TAM, post (*n* = 6; 2M/4F)	*n* = 12 mice
Fig. S3h	*EndoA2*$^{fl/fl}$ + TAM, pre (*n* = 6; 3M/3F)<br>*EndoA2*$^{fl/fl}$ + TAM, post (*n* = 6; 3M/3F)<br>*EndoA2*$^{fl/fl}$ × *AvCreERT2* + TAM, pre (*n* = 6; 2M/4F)<br>*EndoA2*$^{fl/fl}$ × *AvCreERT2* + TAM, post (*n* = 6; 2M/4F)	Same mice as in Fig. S3g
Fig. S3i	*EndoA2*$^{fl/fl}$ + TAM, pre (*n* = 6; 3M/3F)<br>*EndoA2*$^{fl/fl}$ + TAM, post (*n* = 6; 3M/3F)<br>*EndoA2*$^{fl/fl}$ × *AvCreERT2* + TAM, pre (*n* = 6; 3M/3F)<br>*EndoA2*$^{fl/fl}$ × *AvCreERT2* + TAM, post (*n* = 6; 3M/3F)	*n* = 12 mice
Fig. S3j	*EndoA2*$^{fl/fl}$ + TAM, pre (*n* = 6; 3M/3F)<br>*EndoA2*$^{fl/fl}$ + TAM, post (*n* = 6; 3M/3F)<br>*EndoA2*$^{fl/fl}$ × *AvCreERT2* + TAM, pre (*n* = 6; 3M/3F)<br>*EndoA2*$^{fl/fl}$ × *AvCreERT2* + TAM, post (*n* = 6; 3M/3F)	Same mice as in Fig. S3i
Fig. S3k	*EndoA2*$^{fl/fl}$ + TAM, pre (*n* = 6; 3M/3F)<br>*EndoA2*$^{fl/fl}$ + TAM, post (*n* = 6; 3M/3F)<br>*EndoA2*$^{fl/fl}$ × *AvCreERT2* + TAM, pre (*n* = 6; 3M/3F)<br>*EndoA2*$^{fl/fl}$ × *AvCreERT2* + TAM, post (*n* = 6; 3M/3F)	*n* = 12 mice
Fig. S3l	*EndoA2*$^{fl/fl}$ + TAM, pre (*n* = 6; 3M/3F)<br>*EndoA2*$^{fl/fl}$ + TAM, post (*n* = 6; 3M/3F)<br>*EndoA2*$^{fl/fl}$ × *AvCreERT2* + TAM, pre (*n* = 6; 3M/3F)<br>*EndoA2*$^{fl/fl}$ × *AvCreERT2* + TAM, post (*n* = 6; 3M/3F)	Same mice as in Fig. S3k
Fig. S5a	*EndoA2*$^{fl/fl}$ (*n* = 6; 5M/1F)<br>*EndoA2*$^{fl/fl}$ × *Nefh-Cre* (*n* = 6; 3M/3F)<br>Rescue (*n* = 6; 3M/3F)	Same mice as in Fig. 3b
Fig. S5b	*EndoA2*$^{fl/fl}$ (*n* = 6; 5M/1F)<br>*EndoA2*$^{fl/fl}$ × *Nefh-Cre* (*n* = 6; 3M/3F)<br>Rescue (*n* = 6; 3M/3F)	Same mice as in Fig. 3b
Fig. S5c	*EndoA2*$^{fl/fl}$ (*n* = 6; 5M/1F)<br>*EndoA2*$^{fl/fl}$ × *Nefh-Cre* (*n* = 6; 3M/3F)<br>Rescue (*n* = 6; 3M/3F)	Same mice as in Fig. 3b

Fig. S5d	*EndoA2*$^{fl/fl}$ (n = 6; 5M/1F)<br>*EndoA2*$^{fl/fl}$ × *Nefh-Cre* (n = 6; 3M/3F)<br>Rescue (n = 6; 3M/3F)	Same mice as in Fig. 3b
Fig. S5e	*EndoA2*$^{fl/fl}$ (n = 6; 5M/1F)<br>*EndoA2*$^{fl/fl}$ × *Nefh-Cre* (n = 6; 3M/3F)<br>Rescue (n = 6; 3M/3F)	Same mice as in Fig. 3b
Fig. S5f	*EndoA2*$^{fl/fl}$ (n = 6; 5M/1F)<br>*EndoA2*$^{fl/fl}$ × *Nefh-Cre* (n = 6; 3M/3F)<br>Rescue (n = 6; 3M/3F)	Same mice as in Fig. 3b
Fig. S5g	*EndoA2*$^{fl/fl}$ (n = 6; 6M)<br>*EndoA2*$^{fl/fl}$ × *Nefh-Cre* (n = 6; 6M)<br>Rescue (n = 6; 6M)	Same mice as in Fig. 3g
Fig. S6a	*EndoA2*$^{fl/fl}$ (n = 8; 4M/4F)<br>*EndoA2*$^{fl/fl}$ × *Trpv1-Cre* (n = 9; 5M/4F)	n = 17 mice
Fig. S6b	*EndoA2*$^{fl/fl}$ (n = 8; 4M/4F)<br>*EndoA2*$^{fl/fl}$ × *Trpv1-Cre* (n = 9; 5M/4F)	Same mice as in Fig. S6a
Fig. S6c	*EndoA2*$^{fl/fl}$ (n = 8; 4M/4F)<br>*EndoA2*$^{fl/fl}$ × *Trpv1-Cre* (n = 9; 5M/4F)	Same mice as in Fig. S6a
Fig. S6d	*EndoA2*$^{fl/fl}$ (n = 8; 4M/4F)<br>*EndoA2*$^{fl/fl}$ × *Trpv1-Cre* (n = 9; 5M/4F)	Same mice as in Fig. S6a
Fig. S6e	*EndoA2*$^{fl/fl}$ (n = 8; 4M/4F)<br>*EndoA2*$^{fl/fl}$ × *Trpv1-Cre* (n = 9; 5M/4F)	Same mice as in Fig. S6a
Fig. S6f	*EndoA2*$^{fl/fl}$ (n = 8; 4M/4F)<br>*EndoA2*$^{fl/fl}$ × *Trpv1-Cre* (n = 9; 5M/4F)	Same mice as in Fig. S6a
Fig. S6g	*EndoA2*$^{fl/fl}$ (n = 8; 4M/4F)<br>*EndoA2*$^{fl/fl}$ × *Trpv1-Cre* (n = 9; 5M/4F)	Same mice as in Fig. S6a
Fig. S6h	*EndoA2*$^{fl/fl}$ (n = 8; 4M/4F)<br>*EndoA2*$^{fl/fl}$ × *Trpv1-Cre* (n = 9; 5M/4F)	Same mice as in Fig. S6a
Fig. S6i	*EndoA2*$^{fl/fl}$ (n = 8; 4M/4F)<br>*EndoA2*$^{fl/fl}$ × *Trpv1-Cre* (n = 9; 5M/4F)	Same mice as in Fig. S6a

Fig. S6j	*EndoA2*$^{fl/fl}$ (n = 8; 4M/4F)<br>*EndoA2*$^{fl/fl}$ × *Trpv1-Cre* (n = 9; 5M/4F)	Same mice as in Fig. S6a
Fig. S6k	*EndoA2*$^{fl/fl}$ (n = 8; 4M/4F)<br>*EndoA2*$^{fl/fl}$ × *Trpv1-Cre* (n = 9; 5M/4F)	Same mice as in Fig. S6a
Fig. S6l	*EndoA2*$^{fl/fl}$ (n = 8; 4M/4F)<br>*EndoA2*$^{fl/fl}$ × *Trpv1-Cre* (n = 9; 5M/4F)	Same mice as in Fig. S6a
Fig. S6m	*EndoA2*$^{fl/fl}$ (n = 8; 4M/4F)<br>*EndoA2*$^{fl/fl}$ × *Trpv1-Cre* (n = 9; 5M/4F)	Same mice as in Fig. S6a
Fig. S6n	*EndoA2*$^{fl/fl}$ (n = 8; 4M/4F)<br>*EndoA2*$^{fl/fl}$ × *Trpv1-Cre* (n = 9; 5M/4F)	Same mice as in Fig. S6a
Fig. S7i	*EndoA2*$^{fl/fl}$ (n = 8; 4M/4F)<br>*EndoA2*$^{fl/fl}$ × *Nefh-Cre* (n = 8; 4M/4F)	Same mice as in Fig. S7d, f
Fig. S9b	NT-siRNA (n = 4; 4M)<br>EndoA2-siRNA 5 nmol (n = 4; 4M)<br>EndoA2-siRNA 10 nmol (n = 4; 4M)<br>EndoA2-siRNA 20 nmol (n = 4; 4M)	Same NHPs as in Fig. 8b; > 1 week washout between doses
Fig. S9c	NT-siRNA (n = 4; 4M)<br>EndoA2-siRNA 5 nmol (n = 4; 4M)<br>EndoA2-siRNA 10 nmol (n = 4; 4M)<br>EndoA2-siRNA 20 nmol (n = 4; 4M)	Same NHPs as in Fig. 8b; > 1 week washout between doses
Fig. S9d	NT-siRNA (n = 4; 4M)<br>EndoA2-siRNA 5 nmol (n = 4; 4M)<br>EndoA2-siRNA 10 nmol (n = 4; 4M)<br>EndoA2-siRNA 20 nmol (n = 4; 4M)	Same NHPs as in Fig. 8b; > 1 week washout between doses
Molecular biology and electrophysiological experiments		
Fig. 1a	n = 4; 4M	n = 4 mice
Fig. 1b	n = 4; 4M	Same mice as in Fig. 1a
Fig. 1c	Same mice were used in IB4, CGRP and NF200 group (n = 4; 4M)	Same mice as in Fig. 1a
Fig. 1d	Same mice were used in IB4, CGRP and NF200 group (n = 4; 4M)	Same mice as in Fig. 1a
Fig. 1e	n = 4; 4M	Same mice as in

		Fig. 1a
Fig. 1f	n = 4; 4M	Same mice as in Fig. 1a
Fig. 4a	n = 18; 18M	n = 18 mice
Fig. 4b	n = 18; 18M	Same mice as in Fig. 4a
Fig. 4c	n = 18; 18M	Same mice as in Fig. 4a
Fig. 4d	n = 18; 18M	Same mice as in Fig. 4a
Fig. 4e	n = 3; 3M	n = 3 mice
Fig. 4f	n = 3; 3M	Same mice as in Fig. 4e
Fig. 4g	n = 3; 2M/1F	n = 3 mice
Fig. 4j	*EndoA2$^{fl/fl}$* (n = 4; 2M/2F)<br>*EndoA2$^{fl/fl}$* × *Nefh-Cre* (n = 5; 3M/2F)<br>Rescue (n = 5 samples/10 mice; 5M/5F)	n = 19 mice
Fig. 4k	*EndoA2$^{fl/fl}$* (n = 4; 2M/2F)<br>*EndoA2$^{fl/fl}$* × *Nefh-Cre* (n = 5; 3M/2F)<br>Rescue (n = 5 samples/10 mice; 5M/5F)	Same mice as in Fig. 4j
Fig. 4l	*EndoA2$^{fl/fl}$* (n = 4; 2M/2F)<br>*EndoA2$^{fl/fl}$* × *Nefh-Cre* (n = 5; 3M/2F)<br>Rescue (n = 5 samples/10 mice; 5M/5F)	Same mice as in Fig. S4b
Fig. 4m	Statistical charts of Fig. 4J-4L	Same mice as in Fig. 4j and S4b
Fig. 4n-p	*EndoA2$^{fl/fl}$* (n = 3 samples/6 mice; 3M/3F)<br>*EndoA2$^{fl/fl}$* × *Nefh-Cre* (n = 3 samples/6 mice; 6F)<br>Rescue (n = 3 samples/9 mice; 9M)	n = 21 mice
Fig. 5a	*EndoA2$^{fl/fl}$* (n = 3; 3F)<br>*EndoA2$^{fl/fl}$* × *Nefh-Cre* (n = 3; 3F)<br>Rescue (n = 3; 3M)	n = 9 mice
Fig. 5b	*EndoA2$^{fl/fl}$* (n = 3; 3F)<br>*EndoA2$^{fl/fl}$* × *Nefh-Cre* (n = 3; 3F)<br>Rescue (n = 3; 3M)	Same mice as in Fig. 5a
Fig. 5c	*EndoA2$^{fl/fl}$* (n = 3; 3F)<br>*EndoA2$^{fl/fl}$* × *Nefh-Cre* (n = 3; 3F)<br>Rescue (n = 3; 3M)	Same mice as in Fig. 5a
Fig. 5d	Piezo2 + mock (n = 8 cells)<br>Piezo2 + EndoA2 (n = 8 cells)	Cell line; no mice
Fig. 5e	Piezo2 + mock (n = 8 cells)<br>Piezo2 + EndoA2 (n = 8 cells)	Cell line; no mice; same cells as in Fig. 5d
Fig. 5f	Piezo2 + mock (n = 8 cells)<br>Piezo2 + EndoA2 (n = 8 cells)	Cell line; no mice; same cells

		as in Fig. 5d
Fig. 5h	*EndoA2*$^{fl/fl}$ (*n* = 71 neurons/14 mice; 11M/3F)<br>*EndoA2*$^{fl/fl}$ × *Nefh-Cre* (*n* = 68 neurons/14 mice; 10M/4F)<br>Rescue (*n* = 65 neurons/16 mice; 10M/6F)	*n* = 44 mice
Fig. 5i	*EndoA2*$^{fl/fl}$ (*n* = 71 neurons/14 mice; 11M/3F)<br>*EndoA2*$^{fl/fl}$ × *Nefh-Cre* (*n* = 68 neurons/14 mice; 10M/4F)<br>Rescue (*n* = 65 neurons/16 mice; 10M/6F)	Same mice as in Fig. 5h
Fig. 6a	Control (*n* = 5 samples/10 mice; 5M/5F)<br>SH3 domain (*n* = 5 samples/10 mice; 5M/5F)	*n* = 20 mice
Fig. 6b	Control (*n* = 5 samples/10 mice; 5M/5F)<br>SH3 domain (*n* = 5 samples/10 mice; 5M/5F)	Same mice as in Fig. 6a
Fig. 6c	Control (*n* = 5 samples/10 mice; 5M/5F)<br>SH3 domain (*n* = 5 samples/10 mice; 5M/5F)	*n* = 20 mice
Fig. 6d	Statistical charts of Fig. 6A-6C	Same mice as in Fig. 6a and 6c
Fig. 6e	Control (*n* = 5 samples/20 mice; 10M/10F)<br>SH3 domain (*n* = 5 samples/20 mice; 10M/10F)	Same mice as in Fig. 6a and 6c
Fig. 7a	*n* = 18; 18M	*n* = 18 mice
Fig. 7b	*n* = 3; 3M	*n* = 3 mice
Fig. 7d	*EndoA2*$^{fl/fl}$ (*n* = 6 mice; 3M/3F)<br>*EndoA2*$^{fl/fl}$ × *Nefh-Cre* (*n* = 6 mice; 3M/3F)<br>IgG (*n* = 6 mice; 6M)	*n* = 18 mice
Fig. 7e	*EndoA2*$^{fl/fl}$ (*n* = 3 mice; 1M/2F)<br>*EndoA2*$^{fl/fl}$ × *Nefh-Cre* (*n* = 3 mice; 2M/1F)	*n* = 6 mice
Fig. 7f	*n* = 3; 3M	*n* = 3 mice
Fig. 7g	NT-shRNA (*n* = 6 samples/12 mice; 12M)<br>KIF5B-shRNA (*n* = 6 samples/12 mice; 12M)	*n* = 24 mice
Fig. 7o	Control (*n* = 5 samples/10 mice; 10M)<br>KIF5B-over (*n* = 5 samples/10 mice; 10M)<br>KIF5B-over + EndoA2-shRNA (*n* = 4 samples/8 mice; 8M)	*n* = 28 mice
Fig. 7p	Control (*n* = 5 samples/10 mice; 5M/5F)<br>EndoA2-over (*n* = 5 samples/10 mice; 5M/5F)<br>EndoA2-over + KIF5B-shRNA (*n* = 4 samples/8 mice; 4M/4F)	*n* = 28 mice
Fig. 8f	*n* = 3 donors; 2M/1F	*n* = 3 human donors
Fig. 8g	*n* = 3 donors; 2M/1F	Same donors as in Fig. 8f
Fig. S1a	*n* = 6; 6M	*n* = 6 mice
Fig. S1b	Sham (*n* = 6; 6M)<br>SNL (*n* = 6; 6M)	*n* = 6 mice; also same mice as in

		Fig. S1a
Fig. S1c	Same mice were used in spinal dorsal cord, dorsal root, DRG soma and sciatic nerve group (*n* = 3; 3M)	*n* = 3 mice
Fig. S1d	*n* = 4; 4M	Same mice as in Fig. 1a
Fig. S1e	*n* = 4; 4M	Same mice as in Fig. 1a
Fig. S2a	*EndoA2*$^{fl/fl}$ - TAM (*n* = 4; 2M/2F)<br>*EndoA2*$^{fl/fl}$ × *AvCreERT2* - TAM (*n* = 4; 2M/2F)<br>*EndoA2*$^{fl/fl}$ + TAM (*n* = 4; 2M/2F)<br>*EndoA2*$^{fl/fl}$ × *AvCreERT2* + TAM (*n* = 4; 2M/2F)	*n* = 8 mice; also same mice as in Fig. 2b
Fig. S2b	*EndoA2*$^{fl/fl}$ - TAM (*n* = 4; 2M/2F)<br>*EndoA2*$^{fl/fl}$ × *AvCreERT2* - TAM (*n* = 4; 2M/2F)<br>*EndoA2*$^{fl/fl}$ + TAM (*n* = 4; 2M/2F)<br>*EndoA2*$^{fl/fl}$ × *AvCreERT2* + TAM (*n* = 4; 2M/2F)	Same mice as in Fig. S2a
Fig. S4b	*EndoA2*$^{fl/fl}$ (*n* = 4; 2M/2F)<br>*EndoA2*$^{fl/fl}$ × *Nefh-Cre* (*n* = 5; 3M/2F)<br>Rescue (*n* = 5 samples/10 mice; 5M/5F)	*n* = 19 mice
Fig. S4c	*EndoA2*$^{fl/fl}$ (*n* = 4; 2M/2F)<br>*EndoA2*$^{fl/fl}$ × *Nefh-Cre* (*n* = 4; 2M/2F)<br>Rescue (*n* = 4; 2M/2F)	*n* = 12 mice
Fig. S4d	*EndoA2*$^{fl/fl}$ (*n* = 4; 2M/2F)<br>*EndoA2*$^{fl/fl}$ × *Nefh-Cre* (*n* = 4; 2M/2F)<br>Rescue (*n* = 4; 2M/2F)	Same mice as in Fig. S4c
Fig. S4e	*EndoA2*$^{fl/fl}$ (*n* = 4; 2M/2F)<br>*EndoA2*$^{fl/fl}$ × *Nefh-Cre* (*n* = 4; 2M/2F)	Same mice as in Fig. S4c
Fig. S4f	*EndoA2*$^{fl/fl}$ (*n* = 4; 2M/2F)<br>*EndoA2*$^{fl/fl}$ × *Nefh-Cre* (*n* = 4; 2M/2F)	Same mice as in Fig. S4c
Fig. S4g	*EndoA2*$^{fl/fl}$ (*n* = 4; 2M/2F)<br>*EndoA2*$^{fl/fl}$ × *Nefh-Cre* (*n* = 4; 2M/2F)	Same mice as in Fig. S4c
Fig. S4h	*EndoA2*$^{fl/fl}$ (*n* = 4; 2M/2F)<br>*EndoA2*$^{fl/fl}$ × *Nefh-Cre* (*n* = 4; 2M/2F)	Same mice as in Fig. S4c
Fig. S5h	*n* = 4; 2M/2F	Same mice as in Fig. S4c
Fig. S7a	Same mice were used in DRG and sciatic nerve (*n* = 3; 3M)	Same mice as in Fig. 4e
Fig. S7b	*Piezo2*^{WT} (*n* = 3; 3M)<br>*Piezo2*^{CKO} (*n* = 3; 3M)	*n* = 6 mice
Fig. S7c	*EndoA2*$^{fl/fl}$ (*n* = 4 samples/8 mice; 4M/4F)<br>*EndoA2*$^{fl/fl}$ × *Nefh-Cre* (*n* = 5 samples/10 mice; 6M/4F)<br>Rescue (*n* = 5 samples/20 mice; 10M/10F)	Same mice as in Fig. 4j and S4b
Fig. S7d	*EndoA2*$^{fl/fl}$ (*n* = 4; 2M/2F)<br>*EndoA2*$^{fl/fl}$ × *Nefh-Cre* (*n* = 4; 2M/2F)	*n* = 8 mice

Fig. S7e	*EndoA2*$^{fl/fl}$ (n = 4; 2M/2F)<br>*EndoA2*$^{fl/fl}$ × *Nefh-Cre* (n = 4; 2M/2F)	Same mice as in Fig. S7d
Fig. S7f	*EndoA2*$^{fl/fl}$ (n = 4; 2M/2F)<br>*EndoA2*$^{fl/fl}$ × *Nefh-Cre* (n = 4; 2M/2F)	n = 8 mice
Fig. S7g	*EndoA2*$^{fl/fl}$ (n = 4; 2M/2F)<br>*EndoA2*$^{fl/fl}$ × *Trpv1-Cre* (n = 4; 4M)	n = 8 mice
Fig. S7h	*EndoA2*$^{fl/fl}$ (n = 4; 2M/2F)<br>*EndoA2*$^{fl/fl}$ × *Trpv1-Cre* (n = 4; 2M/2F)	n = 8 mice
Fig. S7j	*EndoA2*$^{fl/fl}$ (n = 38 neurons/8 mice; 6M/2F)<br>*EndoA2*$^{fl/fl}$ × *Nefh-Cre* (n = 43 neurons/8 mice; 4M/4F)	n = 16 mice
Fig. S7k	*EndoA2*$^{fl/fl}$ (n = 38 neurons/8 mice; 6M/2F)<br>*EndoA2*$^{fl/fl}$ × *Nefh-Cre* (n = 43 neurons/8 mice; 4M/4F)	Same mice as in Fig. S7j
Fig. S8a	n = 3; 2M/1F	n = 3 mice
Fig. S8b	n = 3; 3M	Same mice as in Fig. 7f
Fig. S8c	n = 3; 3M	Same mice as in Fig. 7f
Fig. S8d	NT-shRNA (n = 3 samples/6 mice; 6M)<br>KIF5B-shRNA (n = 3 samples/6 mice; 6M)	Same mice as in Fig. 7h
Fig. S8e	Control (n = 3 samples/6 mice; 6M)<br>KIF5B-over (n = 3 samples/6 mice; 6M)	n = 12 mice
Fig. S8f	Control (n = 3 samples/6 mice; 3M/3F)<br>EndoA2-over (n = 3 samples/6 mice; 3M/3F)	n = 12 mice
Fig. S9a	NT-siRNA (n = 3 repeats)<br>EndoA2-siRNA (n = 3 repeats)	Cell line; no mice
Fig. S10a	n = 3 donors; 2M/1F	Same donors as in Fig. 8f
Fig. S10b	n = 3 donors; 2M/1F	Same donors as in Fig. 8f
Fig. S10c	n = 2 donors; 2M	N = 2 human donors
Fig. S10d	n = 2 donors; 2M	Same donors as in Fig. S10c
Fig. S10e	n = 3 donors; 2M/1F	Same donors as in Fig. 8f
Total		739 mice; 4 NHPs; 5 donors

M male, *F* female, *NHPs* nonhuman primates

Table S2 The specific primer sequences of endophilins

Gene	Primer	Sequence
EndoA1	Forward	5’-TTCCAGACGAAGAACTCCGC-3’
	Reverse	5’-CTCCAGCTGAGCCTGAACAA-3’
EndoA2	Forward	5’-ATCTGCAACCTAACCCAGCC-3’
	Reverse	5’-CATCTAGCAGAGCATCGCCA-3’
EndoA3	Forward	5’-CTAGAGCCAAAGAGAAGCCCG-3’
	Reverse	5’-TCAGCGATGCTTCACACCAA-3’
EndoB1	Forward	5’-ATTACCCGTCTTCTGCTGGAA-3’
	Reverse	5’-GTTGGCAGCGTCATAATCGT-3’
EndoB2	Forward	5’ CCTAACCCCTTCTGGCTGTG-3’
	Reverse	5’-TGCTGCTGAACCCTCAACTT-3’
GAPDH	Forward	5’-CCCATTCTTCCACCTTTGAT-3’
	Reverse	5’-CAACTGAGGGCCTCTCTCTT-3’
