# Supplementary material for: Endophilin A2 controls touch and mechanical allodynia via kinesin-mediated Piezo2 trafficking
Source: Mil Med Res. 2024 Mar 12;11:17. doi: 10.1186/s40779-024-00520-z (PMC10929226; doi:10.1186/s40779-024-00520-z)
Supplement: Supplementary file 2 — Additional file 2: Fig. S1 SNL increases the expression of EndoA2 in sensory neurons. Fig. S2 Deletion of EndoA2 in DRG neurons did not change the heat hyperalgesia induced by SNL and CFA. Fig. S3 Loss of EndoA2 in DRG neurons suppresses punctate- and brush-evoked CPA in mice with SNL, CFA and VCR treatment. Fig. S4 EndoA2fl/fl × Nefh-Cre mice display normal sensory neurons and their central innervations. Fig. S5 Loss or rescue of EndoA2 in NF200-positive (NF200+) DRG neurons did not alter heat hyperalgesia induced by SNL and CFA. Fig. S6 Deletion of EndoA2 in TRPV1-positive small-diameter DRG neurons did not change the touch and pain behaviors. Fig. S7 Loss of EndoA2 decreases the membrane trafficking of Piezo2 in sciatic nerves. Fig. S8 KIF5B, EndoA2 and Piezo2 are coexpressed in the sciatic nerves of mice. Fig. S9 EndoA2-siRNA does not change the punctate, dynamic and heat threshold of nonhuman primates. Fig. S10 The distribution of EndoA2 in the DRG of humans. [file 40779_2024_520_MOESM2_ESM.pdf]

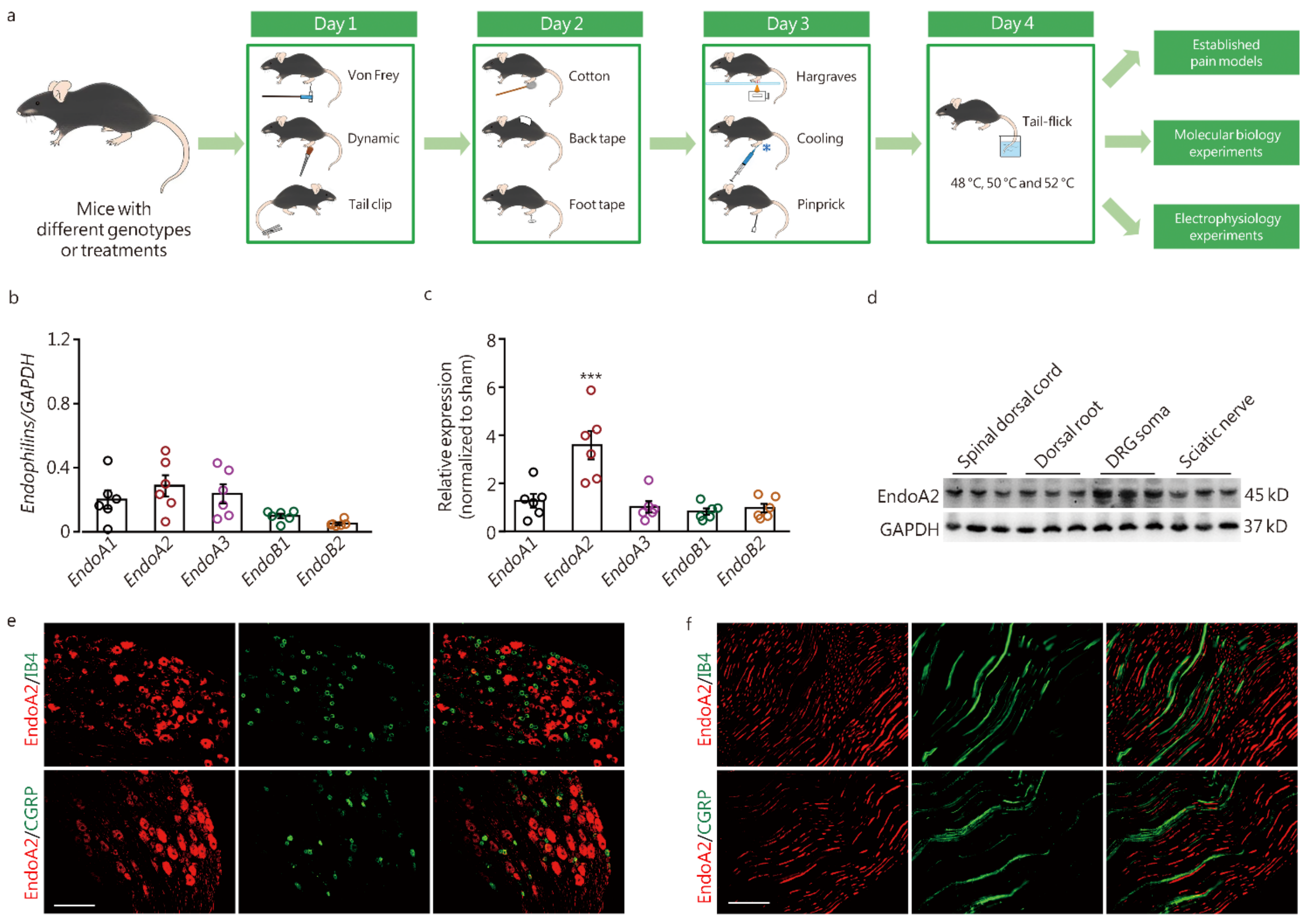

Fig. S1 SNL increases the expression of EndoA2 in sensory neurons. **a** Experimental flow diagrams: the mice with different genotypes or treatments were conducted multiple behavioral tests over a span of 4 d in the following order: von Frey, dynamic and tail clip (Day 1); cotton, back tape and foot tape (Day 2); Hargraves, cooling and pinprick (Day 3); and tail-flick (Day 4). The interval between different tests was at least 2 h. Following behavioral tests, different cohorts of mice were used to perform pain models, molecular biology and electrophysiology experiments. **b** The relative expression abundance of *endophilin* (*A1, A2, A3, B1 and B2*) mRNA in the DRGs of mice. n = 6 mice per group. **c** The relative expression of *endophilin* (*A1, A2, A3, B1 and B2*) mRNA in the DRGs of mice after SNL (Day 7) treatment. n = 6 mice per group. $^{***}P < 0.001$ compared with the corresponding sham group. **d** The expression abundance of EndoA2 in the spinal dorsal cord, dorsal root, DRG soma and sciatic nerve tissues of mice. Double immunostaining of EndoA2 with IB4 and CGRP in the DRG sections (**e**) and

sciatic nerve sections **(f)** of mice. Scale bar = 200 μm (**e**) and 100 μm (**f**). Two-tailed independent Student's *t* test (**c**). The error bars indicate the SEMs. SNL spinal nerve ligation, EndoA1 endophilin A1, EndoA2 endophilin A2, EndoA3 endophilin A3, EndoB1 endophilin B1, EndoB2 endophilin B2, DRG dorsal root ganglion, IB4 isolectin B4, CGRP calcitonin gene-related peptide

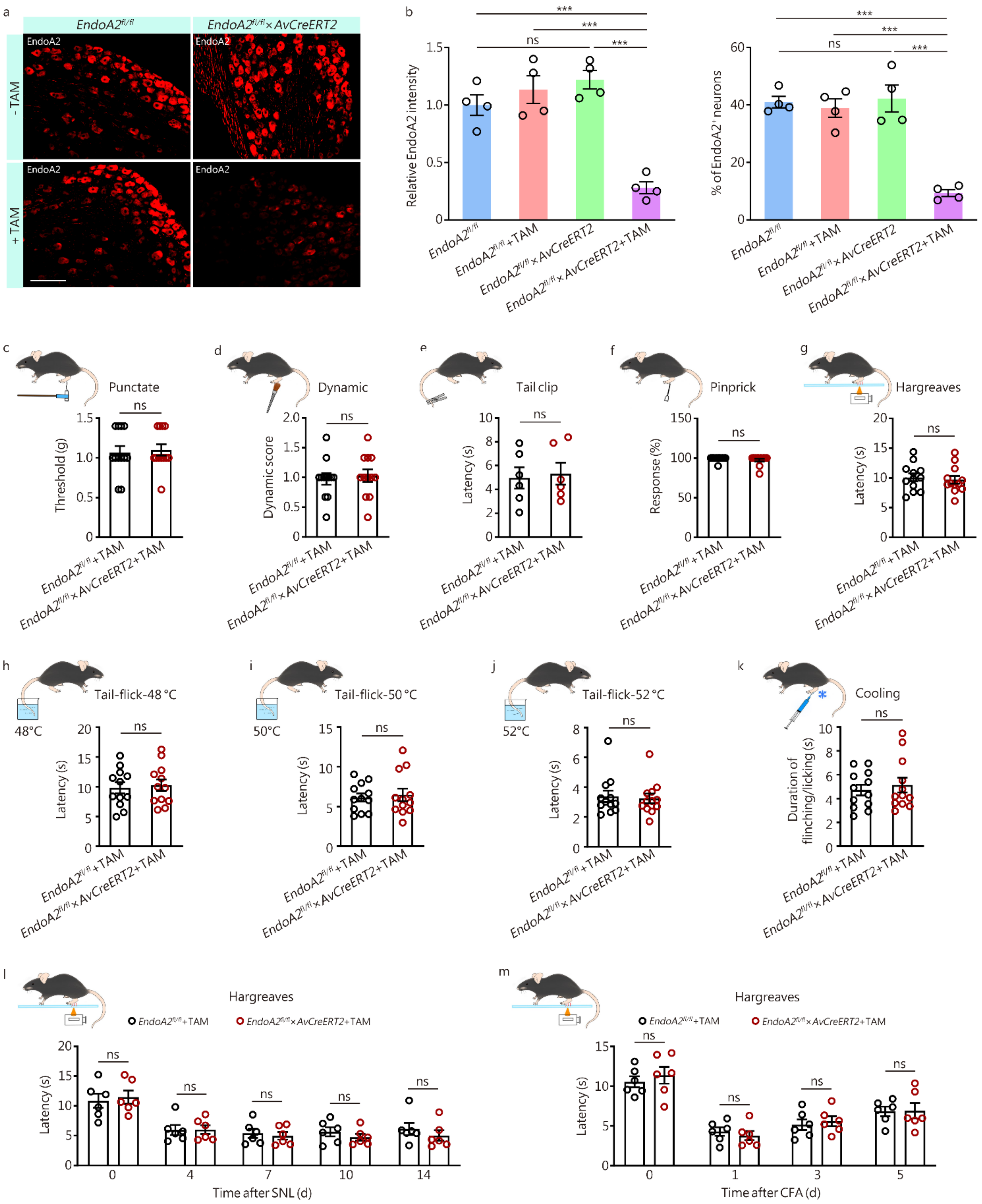

Fig. S2 Deletion of EndoA2 in DRG neurons did not change the heat hyperalgesia induced by SNL and CFA. **a** Immunostaining of EndoA2 in DRG neurons of *EndoA2*$^{fl/fl}$ and *EndoA2*$^{fl/fl}$ × *AvCreERT2-Cre* mice, with or without tamoxifen (TAM) injection. Scale bar = 200 μm. **b** Quantitative analysis

was performed to measure the fluorescence intensity of EndoA2 and the proportion of EndoA2-positive ($EndoA2^+$) neurons in the DRGs of *EndoA2*$^{fl/fl}$ and *EndoA2*$^{fl/fl}$ × *AvCreERT2* mice, with or without TAM injection. $n = 4$. The behaviors of *EndoA2*$^{fl/fl}$ and *EndoA2*$^{fl/fl}$ × *AvCreERT2* mice were evaluated by the punctate (**c**), dynamic (**d**), tail clip (**e**), pinprick (**f**), Hargreaves (**g**), tail-flick (**h-j**) and cooling (**k**) tests after TAM injection. $n = 6 – 12$. The effects of deleting EndoA2 in DRG neurons on the heat hyperalgesia induced by SNL (**l**) and CFA (**m**). $n = 6$. One-way ANOVA followed by Tukey's multiple comparisons test (**b**); Two-tailed independent Student's *t* test (**c**-**k**); Two-way ANOVA followed by Bonferroni's multiple comparisons test (**l**, **m**). $^{***}P < 0.001$, ns non-significant. The error bars indicate the SEMs. EndoA2 endophilin A2, SNL spinal nerve ligation, CFA complete Freund's adjuvant

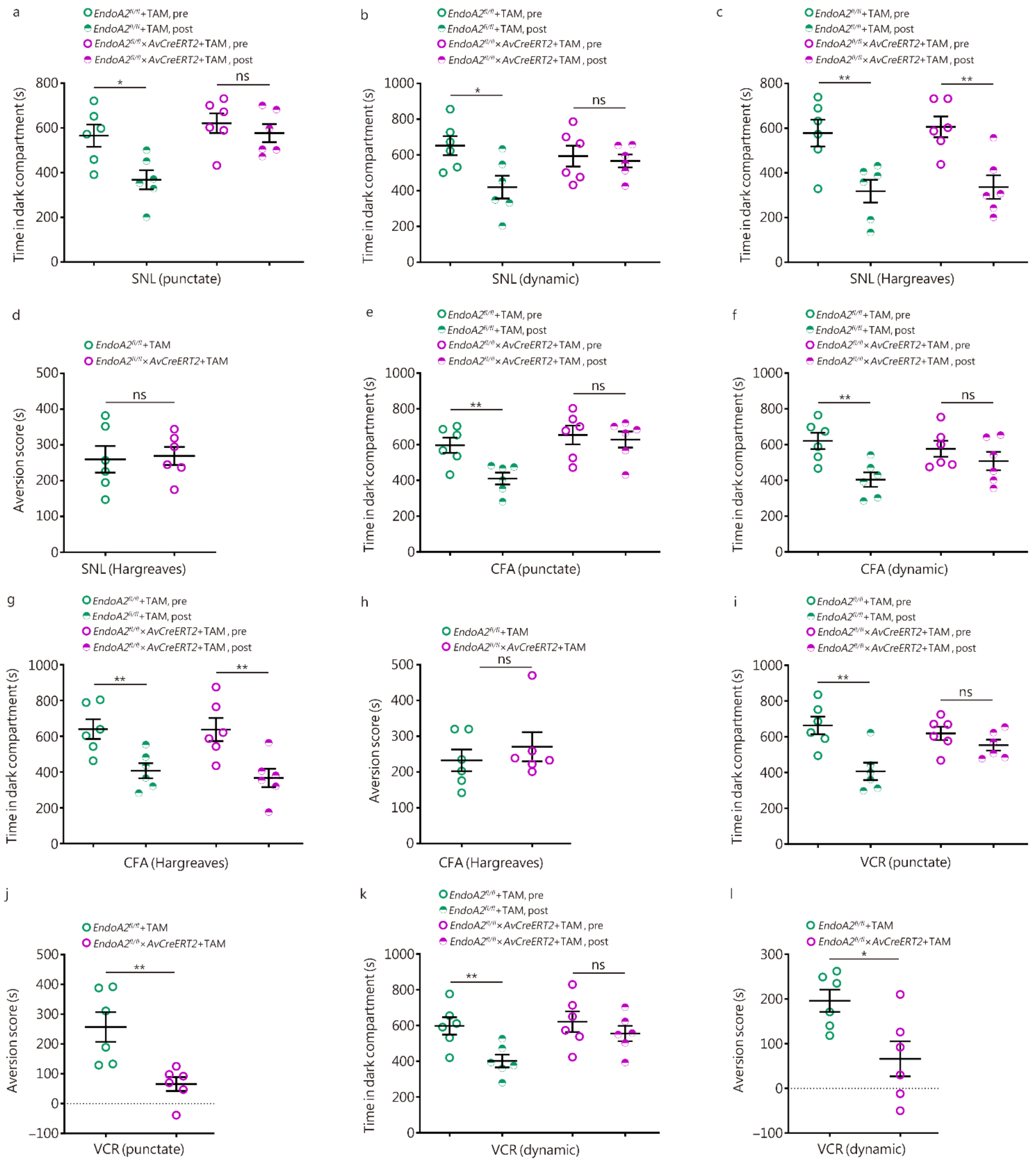

Fig. S3 Loss of EndoA2 in DRG neurons suppresses punctate- and brush-evoked CPA in mice with SNL, CFA and VCR treatment. Absolute time spent in the dark A chamber before (pre) vs. after (post) punctate (**a**), dynamic (**b**) and Hargreaves (**c**) conditioning in *EndoA2*$^{fl/fl}$ and *EndoA2*$^{fl/fl}$ × *AvCreERT2* mice with SNL treatment. $n = 6$. **d** The effect of EndoA2 knockout in DRG neurons on the CPA scores

induced by Hargreaves training in SNL mice (the CPA score was defined by the difference in the amount of time that the mice stayed in the dark A chamber before and after training: pre – post). *n* = 6. Absolute time spent in the dark A chamber before (pre) vs. after (post) punctate (**e**), dynamic (**f**) and Hargreaves (**g**) conditioning in $EndoA2^{fl/fl}$ and $EndoA2^{fl/fl}$ × *AvCreERT2* mice with CFA treatment. *n* = 6. **h** The effect of deleting EndoA2 in DRG neurons on the CPA scores induced by Hargreaves training in CFA mice (the CPA score was defined by the difference in the amount of time that the mice stayed in the dark A chamber before and after training: pre – post). *n* = 6. **i-l** The effect of EndoA2 knockout in DRG neurons on the absolute time spent in the dark A chamber before (pre) vs. after (post) conditioning (punctate, dynamic) and on the CPA scores induced by punctate and dynamic in VCR mice. *n* = 6. Two-tailed independent Student's *t* test (**a-l**). $^{*}P < 0.05$, $^{**}P < 0.01$, ns non-significant. The error bars indicate the SEMs. EndoA2 endophilin A2, TAM tamoxifen, SNL spinal nerve ligation, CFA complete Freund's adjuvant, VCR vincristine

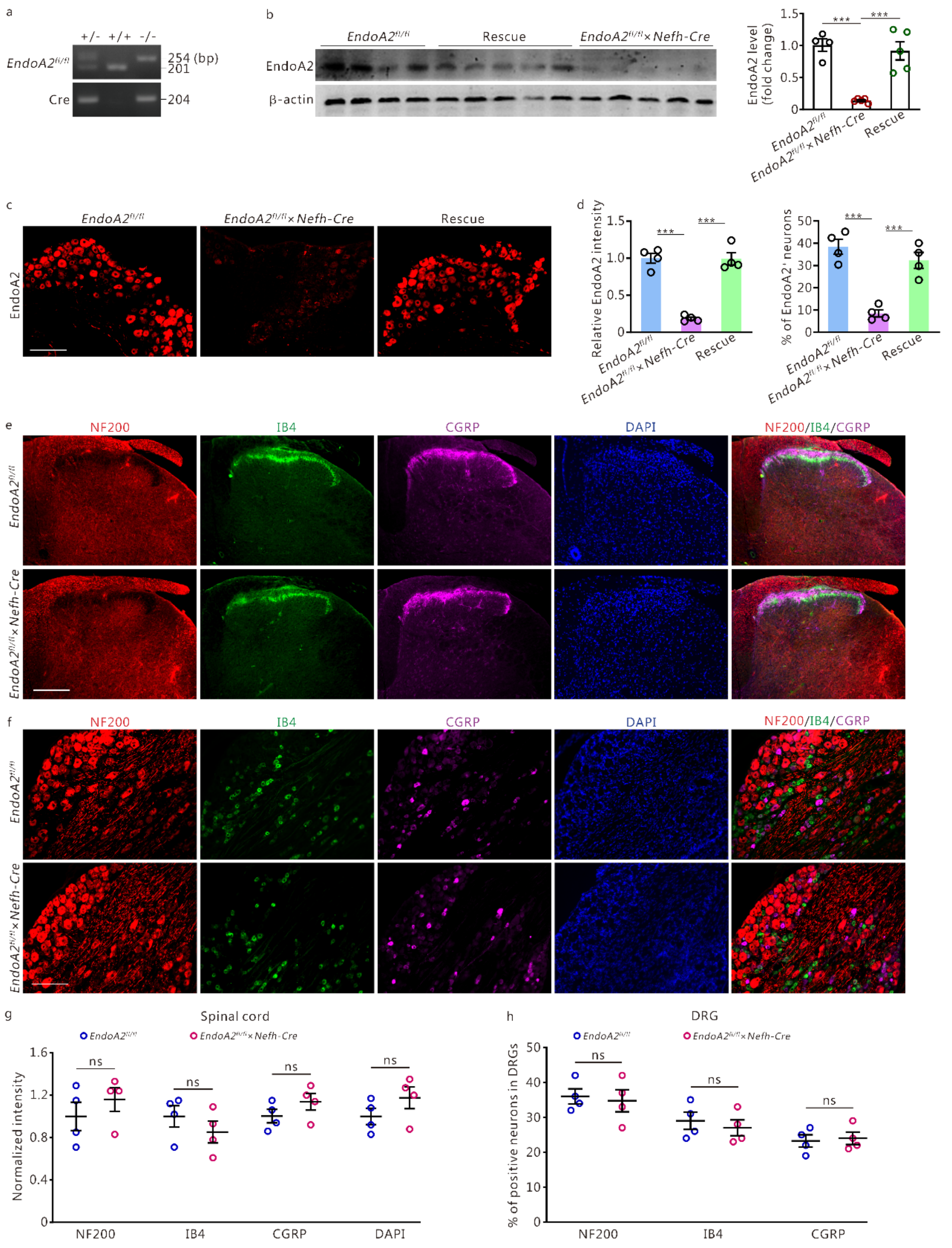

Fig. S4 *EndoA2^fl/fl^* × *Nefh-Cre* mice display normal sensory neurons and their central innervations. **a** Genotyping of EndoA2 conditional knockout mice for *EndoA2^fl/fl^* and *Nefh-Cre* genotypes. **b** The expression of EndoA2 in DRGs of *EndoA2^fl/fl^*, *EndoA2^fl/fl^* × *Nefh-Cre*, and rescue mice was tested by immunoblotting. n = 4 – 5 samples per group. **c** The expression of EndoA2 in DRGs of *EndoA2^fl/fl^*, *EndoA2^fl/fl^* × *Nefh-Cre*, and rescue mice was determined using immunostaining. Scale bar = 200 μm.

d Quantitative analysis was performed to measure the fluorescence intensity of EndoA2 and the proportion of EndoA2-positive ($EndoA2^{+}$) neurons in the DRGs of *EndoA2$^{fl/fl}$*, *EndoA2$^{fl/fl}$* × *Nefh-Cre*, and rescue mice in **c**. $n = 4$ mice per group. **e** Immunostaining for NF200, IB4 and CGRP in lumbar spinal cord sections from *EndoA2$^{fl/fl}$* and *EndoA2$^{fl/fl}$* × *Nefh-Cre* mice. Scale bar = 200 μm. **f** Immunostaining for NF200, IB4 and CGRP in L_4 DRG sections from *EndoA2$^{fl/fl}$* and *EndoA2$^{fl/fl}$* × *Nefh-Cre* mice. Scale bar = 200 μm. **g** Quantification of immunofluorescence of NF200, IB4, CGRP, and DAPI staining in the dorsal horn of *EndoA2$^{fl/fl}$* and *EndoA2$^{fl/fl}$* × *Nefh-Cre* mice in **e**. $n = 4$. **h** Quantification of the percentages of NF200-positive, IB4-positive and CGRP-positive neurons in the DRG of *EndoA2$^{fl/fl}$* and *EndoA2$^{fl/fl}$* × *Nefh-Cre* mice in **f**. $n = 4$. One-way ANOVA followed by Tukey's multiple comparisons test (**b**, **d**); Two-tailed independent Student's *t* test (**g**, **h**). $^{***}P < 0.001$, ns non-significant. The error bars indicate the SEMs. EndoA2 endophilin A2, NF200 neurofilament-200, CGRP calcitonin gene-related peptide, IB4 isolectin B4

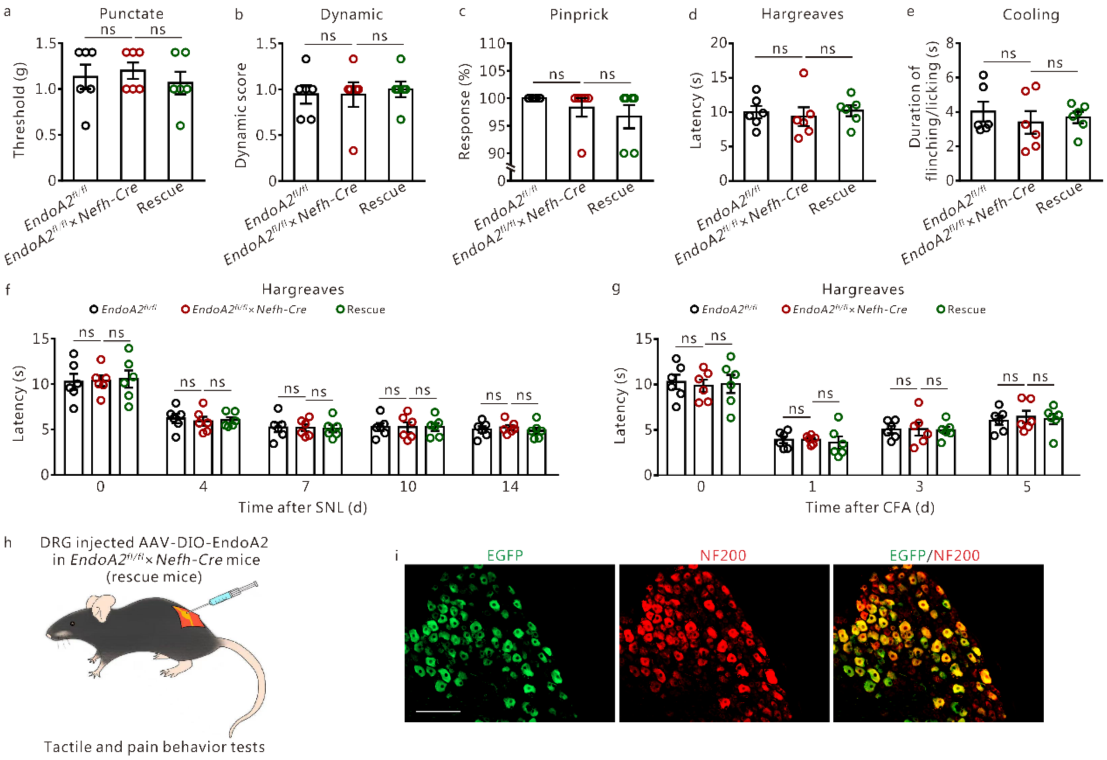

Fig. S5 Loss or rescue of EndoA2 in NF200-positive (NF200^{+}) DRG neurons did not alter heat hyperalgesia induced by SNL and CFA. The behaviors of *EndoA2$^{fl/fl}$*, *EndoA2$^{fl/fl}$* × *Nefh-Cre* and rescued mice were evaluated by the punctate (**a**), dynamic (**b**), pinprick (**c**), Hargreaves (**d**) and cooling (**e**) tests. n = 6. The effects of conditional knockout and rescue of EndoA2 in NF200^{+} DRG neurons on the heat hyperalgesia induced by SNL (**f**) and CFA (**g**). All the SNL and CFA mice underwent punctate, dynamic, and Hargreaves behavioral tests on the same day with 2-h intervals between each test. n = 6. **h** *EndoA2$^{fl/fl}$* × *Nefh-Cre* mice were administered rAAV-Syn-DIO-EndoA2-2A-EGFP via DRG (L_4 and L_5) injection to rescue the expression of EndoA2 in NF200^{+} DRG neurons (rescue mice), and then pain and tactile behavior were tested. **i** Confocal images of EGFP (green) overlapping with NF200 immunoreactivity in DRG sections from *EndoA2$^{fl/fl}$* × *Nefh-Cre* mice after DRG injection of rAAV-Syn-DIO-EndoA2-2A-EGFP. Scale bar = 200 μm. One-way ANOVA followed by Tukey's multiple comparisons test (**a-e**); Two-way ANOVA followed by Bonferroni's multiple comparisons test (**f, g**). ns non-significant. The error bars indicate the SEMs. EndoA2 endophilin A2, SNL spinal

nerve ligation, CFA complete Freund's adjuvant, DRG dorsal root ganglion, NF200 neurofilament-200

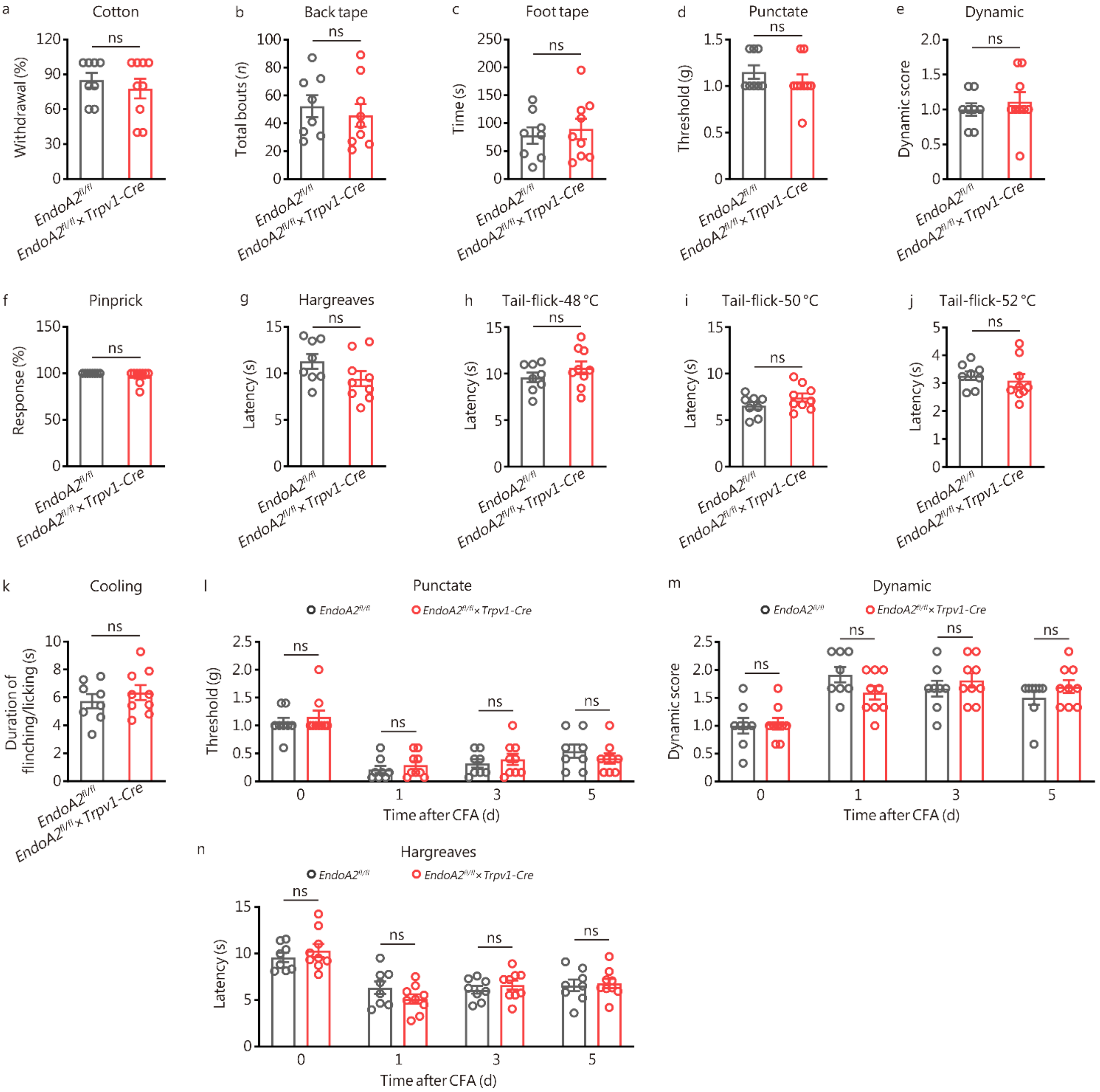

Fig. S6 Deletion of EndoA2 in TRPV1-positive small-diameter DRG neurons did not change the touch and pain behaviors. The touch and pain behaviors of *EndoA2*$^{fl/fl}$ and *EndoA2*$^{fl/fl}$ × *Trpv1-Cre* mice were evaluated by cotton (**a**), back tape (**b**), foot tape (**c**), punctate (**d**), dynamic (**e**), pinprick (**f**), Hargreaves (**g**), tail-flick (**h-j**) and cooling (**k**) tests. The same groups of mice were used for each pain behavioral test. The inter-test interval was at least 2 h. $n = 8 – 9$. **l-n** The effects of conditional knockout of EndoA2 in TRPV1-positive DRG neurons on the mechanical allodynia and heat hyperalgesia induced by CFA. All the mice underwent punctate, dynamic, and Hargreaves behavioral tests on the same day with 2-h

intervals between each test. $n = 8 – 9$. Two-tailed independent Student's t test (**a-k**); Two-way ANOVA followed by Bonferroni's multiple comparisons test (**l-n**). ns non-significant. The error bars indicate the SEMs. EndoA2 endophilin A2, CFA complete Freund's adjuvant

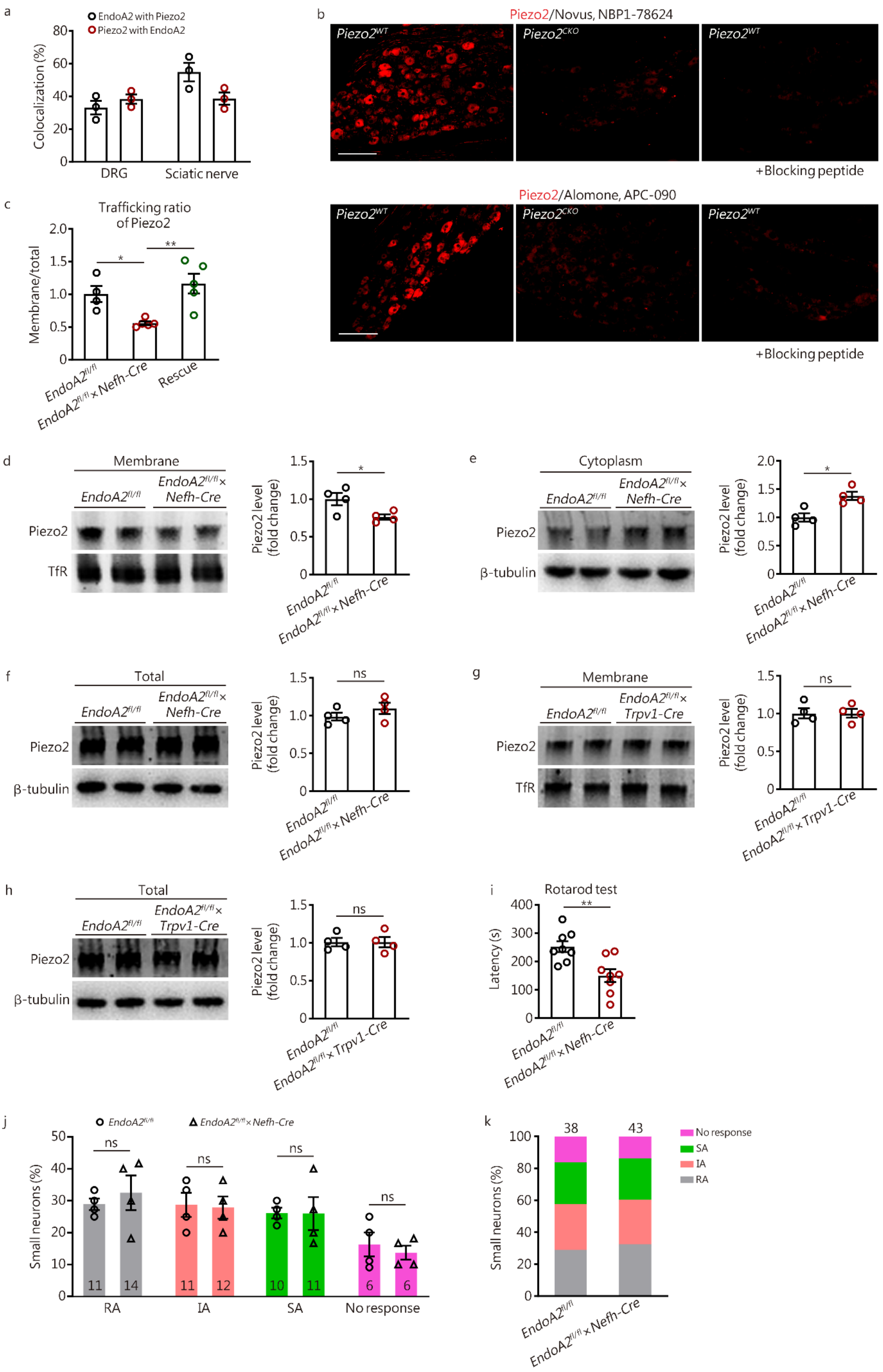

a
EndoA2 with Piezo2
Piezo2 with EndoA2
Colocalization (%)
DRG
Sciatic nerve
b
Piezo2/Novus, NBP1-78624
Piezo2/Alomone, APC-090
+Blocking peptide
c
Trafficking ratio of Piezo2
Membrane/total
Rescue
d
Membrane
Piezo2
TfR
Piezo2 level (fold change)
e
Cytoplasm
β-tubulin
f
Total
g
Membrane
h
Total
i
Rotarod test
Latency (s)
j
Small neurons (%)
RA
IA
SA
No response
k
No response
SA
IA
RA

Fig. S7 Loss of EndoA2 decreases the membrane trafficking of Piezo2 in sciatic nerves. **a** Quantification data show the colocalization rates of EndoA2 with Piezo2 (colocalized yellow spots/total EndoA2-positive spots) and those of Piezo2 with EndoA2 (colocalized yellow spots/total Piezo2-positive spots) in DRG neurons and sciatic nerves in **Fig. 4e** and **f**. $n = 3$. **b** The specificity of Piezo2 antibodies (from two companies) was determined in Piezo2 sensory neuron conditional knockout mice (*Piezo2*$^{fl/fl}$ mice crossed with *AvCreERT2* mice) and with Piezo2 blocking peptide. Scale bar = 200 μm. **c** The trafficking ratio of Piezo2 in DRGs of *EndoA2*$^{fl/fl}$, *EndoA2*$^{fl/fl}$ × *Nefh-Cre* and rescue mice. $n = 4 – 5$ samples per group. Piezo2 expression in the sciatic nerve membrane fraction (**d**), cytoplasmic fraction (**e**) and total lysate (**f**) from *EndoA2*$^{fl/fl}$ and *EndoA2*$^{fl/fl}$ × *Nefh-Cre* mice. $n = 4$. The membrane (**g**) and total (**h**) expression of Piezo2 in the DRG of *EndoA2*$^{fl/fl}$ and *EndoA2*$^{fl/fl}$ × *Trpv1-Cre* mice. $n = 4$. **i** The proprioception behaviors (motor coordination) of *EndoA2*$^{fl/fl}$ and *EndoA2*$^{fl/fl}$ × *Nefh-Cre* mice were evaluated by the rotarod test. $n = 8$. The percentage of small DRG neurons (< 20 μm) from *EndoA2*$^{fl/fl}$ and *EndoA2*$^{fl/fl}$ × *Nefh-Cre* mice that respond to mechanical stimulation, with MA currents characterized by their inactivation kinetics. The percentage of neurons from 4 separate experiments (**j**) ($n = 8 – 12$ neurons per condition and per experiment) or the proportion from all neurons pooled from all 4 experiments (**k**). $n = 38$ and 43 neurons in the *EndoA2*$^{fl/fl}$ and *EndoA2*$^{fl/fl}$ × *Nefh-Cre* groups, respectively. One-way ANOVA followed by Tukey's multiple comparisons test (**c**); Two-tailed independent Student's *t* test (**d-j**). $^{*}P < 0.05$, $^{**}P < 0.01$, ns non-significant. The error bars indicate the SEMs. EndoA2 endophilin A2, DRG dorsal root ganglion, TfR transferrin receptor, RA rapidly adapting, IA intermediately adapting, SA slowly adapting

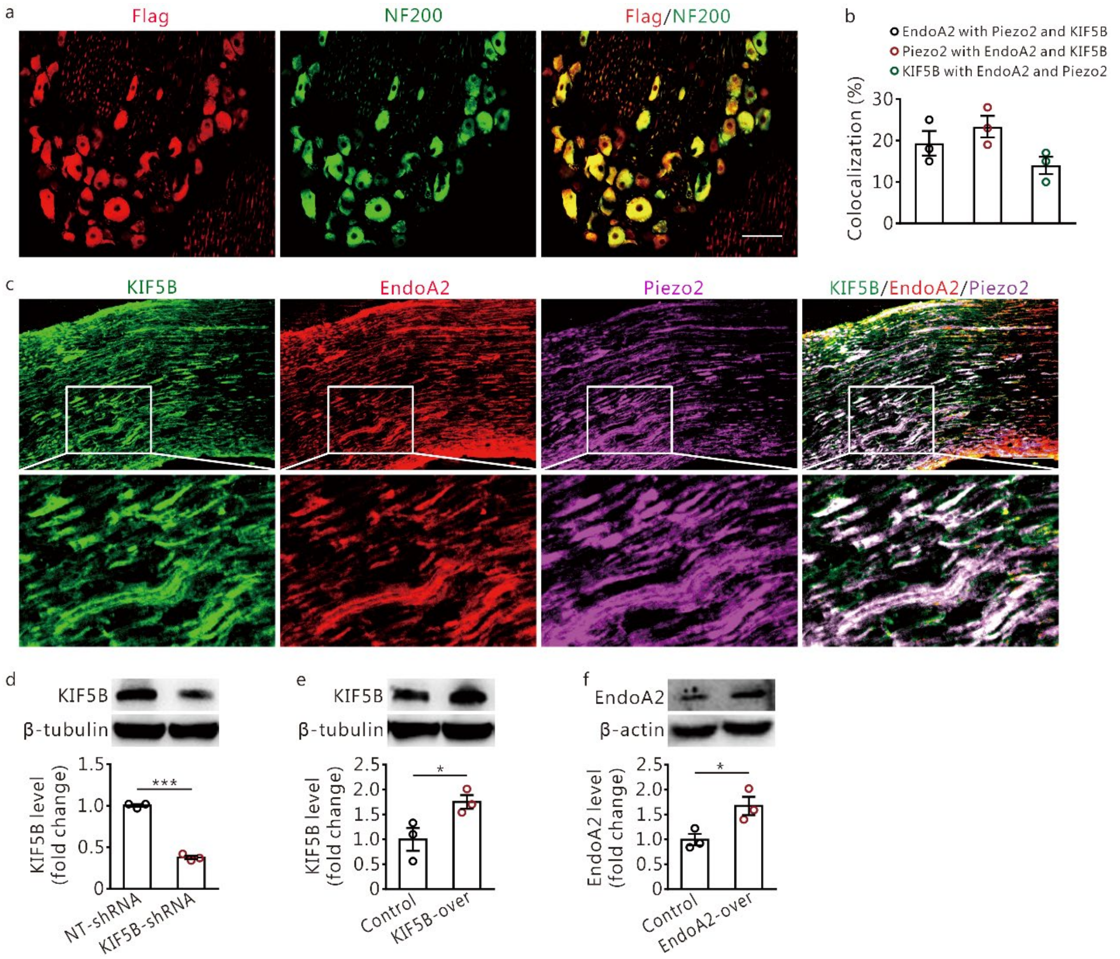

Fig. S8 KIF5B, EndoA2 and Piezo2 are coexpressed in the sciatic nerves of mice. **a** Immunostaining images of Flag overlapping with NF200 in DRG sections from *Nefh-Cre* mice after DRG injection of rAAV-Syn-DIO-EndoA2-SH3-domain-Flag. Scale bar = 100 μm. **b** Quantification data show colocalization rates of EndoA2 with Piezo2 and KIF5B (colocalized white spots/total EndoA2-positive spots), of Piezo2 with EndoA2 and KIF5B (colocalized white spots/total Piezo2-positive spots) and of KIF5B with EndoA2 and Piezo2 (colocalized white spots/total KIF5B-positive spots) in DRG neurons from **Fig. 7f**. $n = 3$. **c** Colocalization of KIF5B, EndoA2 and Piezo2 in mouse sciatic nerves. Scale bar = 100 μm. **d** The expression of KIF5B in DRG tissues 21 d after DRG injection with AAV encoding KIF5B-shRNA. $n = 3$. **e** The expression of KIF5B in DRG tissues 21 d after DRG injection with AAV encoding KIF5B. $n = 3$. **f** The expression of EndoA2 in DRG tissues 21 d after DRG injection with AAV encoding EndoA2. $n = 3$. Two-tailed independent Student's t test (**d-f**). $^{*}P < 0.05$, $^{***}P < 0.001$.

The error bars indicate the SEMs. NF200 neurofilament-200, EndoA2 endophilin A2, NT nontargeting

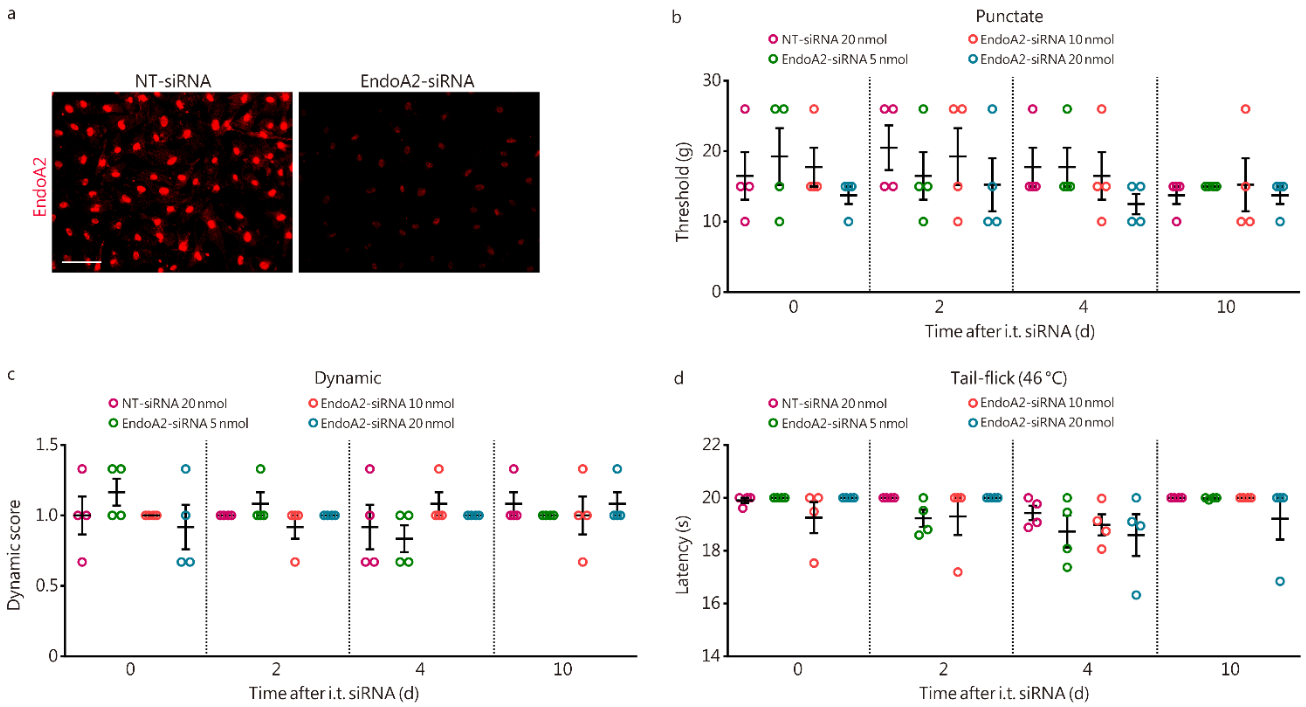

Fig. S9 EndoA2-siRNA does not change the punctate, dynamic and heat threshold of nonhuman primates. **a** Immunostaining results show the expression of EndoA2 by EndoA2-siRNA treatment (50 nmol/L, 48 h) in monkey Vero cells. Scale bar = 100 μm. Punctate (**b**), dynamic (**c**) and tail-flick (**d**) threshold changes in monkeys after intrathecal injection of EndoA2-siRNA (5, 10, and 20 nmol). n = 4. Two-way ANOVA followed by Bonferroni's multiple comparisons test (**b-d**). The error bars indicate the SEMs. NT nontargeting, EndoA2 endophilin A2, i.t. intrathecal injection

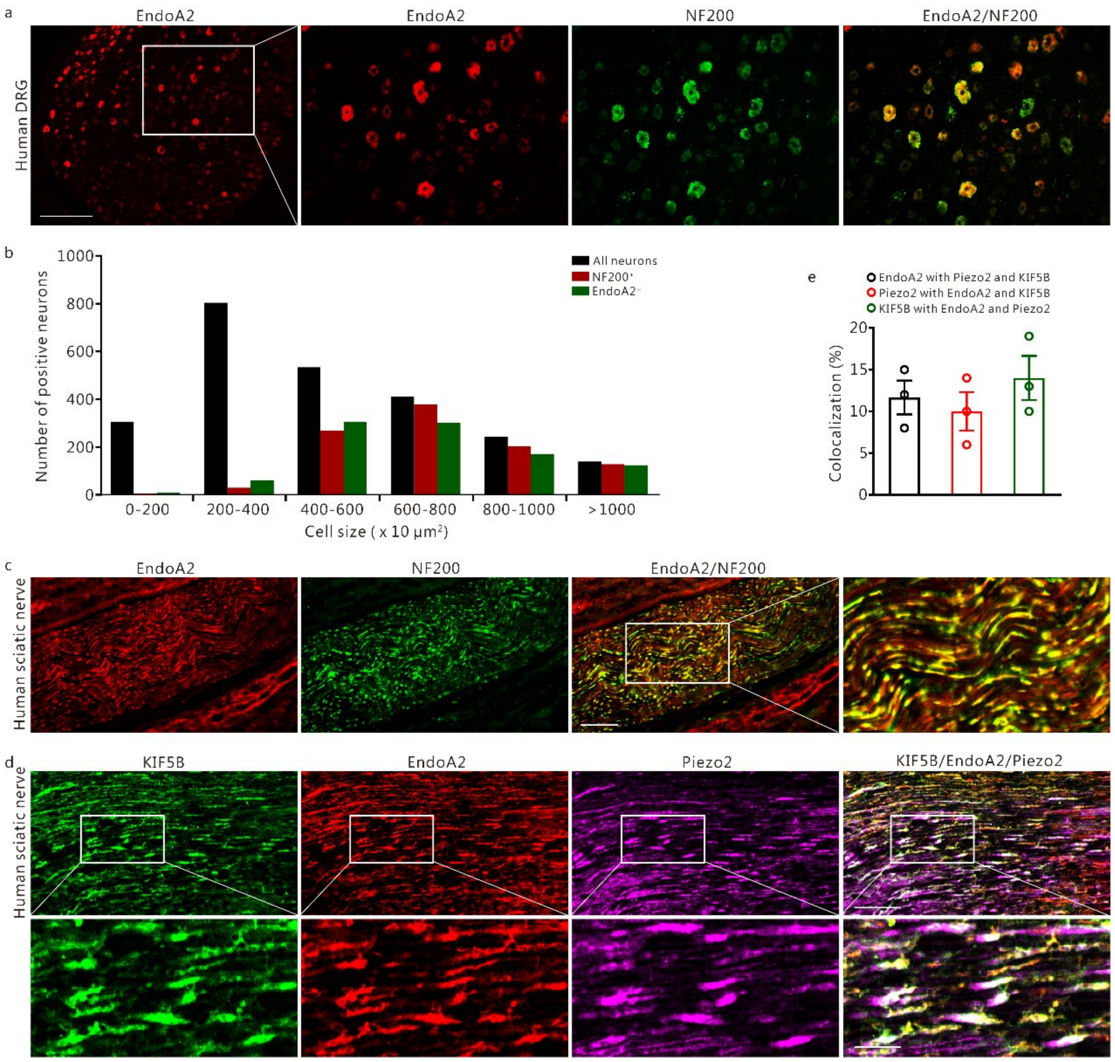

Fig. S10 The distribution of EndoA2 in the DRG of humans. **a** Double labeling of EndoA2 with NF200 in human thoracic DRG sections. Scale bar = 500 μm. **b** Size frequency distribution of EndoA2-positive (EndoA2$^+$), NF200-positive (NF200$^+$) and total neurons in human thoracic DRG sections. A total of 2409 neurons from 3 human DRGs were analyzed. **c** Double immunostaining of EndoA2 with NF200 in human sciatic nerve sections. Scale bar = 100 μm. **d** Colocalization of KIF5B, EndoA2 and Piezo2 in human sciatic nerve sections. Scale bar = 100 μm. **e** Quantification data show colocalization proportion of EndoA2 with Piezo2 and KIF5B (colocalized white spots/total EndoA2 positive spots), of Piezo2 with EndoA2 and KIF5B (colocalized white spots/total Piezo2 positive spots) and those of KIF5B with EndoA2 and Piezo2 (colocalized white spots/total KIF5B positive spots) in human

thoracic DRG neurons of **Fig. 8g**. *n* = 3. EndoA2 endophilin A2, DRG dorsal root ganglion, NF200 neurofilament-200
